# A streamlined workflow for conversion, peer review, and publication of genomics metadata as omics data papers

**DOI:** 10.1093/gigascience/giab034

**Published:** 2021-05-13

**Authors:** Mariya Dimitrova, Raïssa Meyer, Pier Luigi Buttigieg, Teodor Georgiev, Georgi Zhelezov, Seyhan Demirov, Vincent Smith, Lyubomir Penev

**Affiliations:** Pensoft Publishers, Prof. Georgi Zlatarski Street 12, 1700 Sofia, Bulgaria; Institute of Information and Communication Technologies, Bulgarian Academy of Sciences, Acad. G. Bonchev St., Block 25A, 1113 Sofia, Bulgaria; Alfred-Wegener-Institut, Helmholtz-Zentrum für Polar- und Meeresforschung, Am Handelshafen 12, 27570 Bremerhaven, Germany; Alfred-Wegener-Institut, Helmholtz-Zentrum für Polar- und Meeresforschung, Am Handelshafen 12, 27570 Bremerhaven, Germany; Pensoft Publishers, Prof. Georgi Zlatarski Street 12, 1700 Sofia, Bulgaria; Pensoft Publishers, Prof. Georgi Zlatarski Street 12, 1700 Sofia, Bulgaria; The Natural History Museum, Cromwell Rd, South Kensington, SW7 5BD London, UK; Pensoft Publishers, Prof. Georgi Zlatarski Street 12, 1700 Sofia, Bulgaria; Institute of Biodiversity and Ecosystem Research, Bulgarian Academy of Sciences, 2 Gagarin St., 1113 Sofia, Bulgaria

**Keywords:** data, data paper, omics, genomics, metadata, workflow, standards, FAIR principles, MIxS, MINSEQE

## Abstract

**Background:**

Data papers have emerged as a powerful instrument for open data publishing, obtaining credit, and establishing priority for datasets generated in scientific experiments. Academic publishing improves data and metadata quality through peer review and increases the impact of datasets by enhancing their visibility, accessibility, and reusability.

**Objective:**

We aimed to establish a new type of article structure and template for omics studies: the omics data paper. To improve data interoperability and further incentivize researchers to publish well-described datasets, we created a prototype workflow for streamlined import of genomics metadata from the European Nucleotide Archive directly into a data paper manuscript.

**Methods:**

An omics data paper template was designed by defining key article sections that encourage the description of omics datasets and methodologies. A metadata import workflow, based on REpresentational State Transfer services and Xpath, was prototyped to extract information from the European Nucleotide Archive, ArrayExpress, and BioSamples databases.

**Findings:**

The template and workflow for automatic import of standard-compliant metadata into an omics data paper manuscript provide a mechanism for enhancing existing metadata through publishing.

**Conclusion:**

The omics data paper structure and workflow for import of genomics metadata will help to bring genomic and other omics datasets into the spotlight. Promoting enhanced metadata descriptions and enforcing manuscript peer review and data auditing of the underlying datasets brings additional quality to datasets. We hope that streamlined metadata reuse for scholarly publishing encourages authors to create enhanced metadata descriptions in the form of data papers to improve both the quality of their metadata and its findability and accessibility.

## Introduction

The term “omics" refers to the study of biological systems through the examination of different elements of the molecular basis of life. Many fields of molecular biology, thus, derive their name from the suffix “omics" (e.g., genomics, transcriptomics, metabolomics). The genome is examined through the analysis of gene (DNA) sequences, the transcriptome is the collection of all messenger RNA molecules in an organism, and the metabolome is the collection of all metabolites and intermediate substrates participating in the metabolic pathways. Omic studies are generating large quantities of deeply minable data with increasing scale and complexity [1, 2]. Furthermore, omics technologies and approaches have revolutionized biodiversity science [3–5].

Independently from the recent advances in omics technologies and data generation, however, the published omics biodiversity data and their accompanying, standardized metadata are still neither harmonized nor interoperable [6]. Existing infrastructures in omics data science focus on the sequence or molecular data generated from omics studies. Since 1988, the databases of the International Nucleotide Sequence Database Collaboration (INSDC) [7–9] have provided a trusted archive for these data. In parallel, major infrastructures to handle higher-order biodiversity data (e.g., occurrences linked to taxa, specimen records) have emerged and include the Global Biodiversity Information Facility (GBIF) [10], the Integrated Digitized Biocollections (iDigBio) [11], the Distributed System of Scientific Collections (DiSSCo) [12], the Ocean Biogeographic Information System (OBIS) [13], the Global Genome Biodiversity Network (GGBN) [14], DataONE [15], and others. Some of these infrastructures support data repositories that follow community-accepted metadata standards. GBIF uses the Ecological Metadata Language (EML) standard for describing ecological datasets in XML files [16], whereas biodiversity data are recorded by following the Darwin Core Standard (DwC) [17, 18]. Likewise, the GGBN have developed their own GGBN Data Standard, which interoperates with DwC and the Access to Biological Collections Data (ABCD) schema for primary biodiversity data [19–21]. The INSDC cooperates with community standards initiatives such as the Genomic Standards Consortium (GSC) to implement their Minimum Information about any (x) Sequence (MIxS) checklists for genomic, metagenomic, and environmental metadata descriptors, and with the Global Microbial Identifier (GMI) group for pathogen sequence metadata [22, 23]. MIxS consists of 3 checklists, each containing several packages for the description of various environments from which genomic material could be sampled [23]. Other international data repositories such as EBI EMBL's ArrayExpress [24] and the BioSamples [25] database implement standards such as Minimum Information about a high-throughput nucleotide SEQuencing Experiment (MINSEQE) and Minimum Information About a Microarray Experiment (MIAME) and various MIxS environmental checklists [26]. Databases such as the Genomic Observatories Metadatabase (GeOMe) [27] offer integrative solutions for the data management of genomic, geographical, and ecological metadata by providing mechanisms to create standard-compliant templates tailored to specific use cases and linking metadata to dataset records via stable identifiers [28].

A more comprehensive approach towards omics metadata mobilization is undertaken by the ISA Commons community [29], which uses the “extensible, cross-domain format" ISA-Tab for organising metadata [30]. This format focuses on 3 major components of any scientific research: “Investigation," “Study," and “Assay" to help structure the underlying study and assay-specific metadata records [30]. Software for creating and validating ISA-Tab files has also been developed as part of the ISA Tools framework [30]. This framework aims to complement existing omics standards to improve the description of research outputs in the field of omics. Several omics data repositories, such as EMBL-EBL's Metabolights [31] and *GigaScience*’s GigaDB [32], have utilized the ISA model and serializations to integrate experimental metadata.

There are different ways that scientists can publish their data in a FAIR (Findable, Accessible, Interoperable, and Reusable) [33, 34] manner; however, all can be attributed to 2 main routes: (i) data publishing through international trusted data repositories, such as INSDC [7] and GBIF [10]; and (ii) scholarly data publishing in the form of data papers or as data underpinning a research article [3,5–38]. While the first route focuses on data aggregation, standardization, and reuse, the second one augments the quality and reusability of data and metadata through peer review and data auditing in the scholarly publishing process. Scholarly data publishing provides an opportunity to enhance the original metadata in the data paper narrative and to link it to the original dataset via stable identifiers, thus improving the reproducibility and findability of the data [39]. Furthermore, it creates a citable scientific record, enabling the crediting and acknowledgement of the data creators and researchers. Academic publishing involves dissemination of research through additional channels, such as journal distribution networks, and creates increased opportunities for open science collaboration [39].

**Figure 1:**
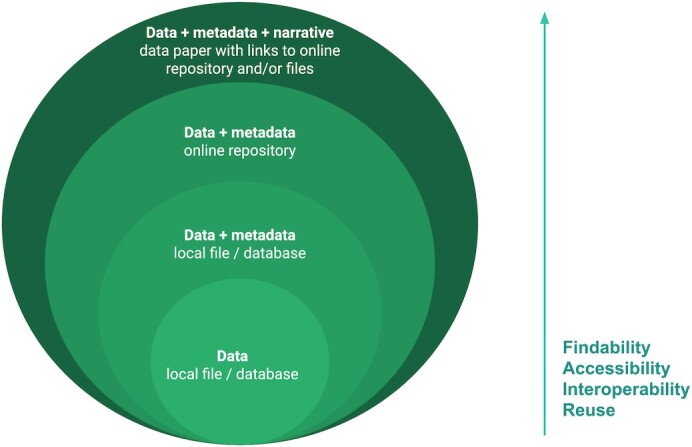
The different layers of FAIRness of data and metadata. Describing data and metadata in a data paper publication helps to enhance their FAIRness through provision of better visibility and accessibility.

While standards and infrastructures are crucial to the advancement of data sharing and reuse within the field of omics, we argue that incentivizing authors to publish their data in the form of peer-reviewed journal articles (data papers) creates the driving force towards a truly FAIR data world (Fig. 1).

As more and more researchers want to deposit and share their datasets, new tools and new approaches are needed to deliver FAIR data. Following the example set by Chavan and Penev [39], who introduced data papers in biodiversity science, we have established a concept for an omics data paper—a type of scholarly paper in which data, generated in genomic or other omic experiments, are described with extended and peer-reviewed metadata, and linked to the corresponding dataset(s) deposited in an INSDC database or other archive. To further incentivize authors to publish omics data papers and to demonstrate the importance of high-quality metadata, we propose a prototype of a streamlined workflow for conversion of European Nucleotide Archive (ENA) genomic metadata directly into a data paper manuscript. We build upon previous work by Pensoft, namely, workflows for automatic import of EML metadata from GBIF, DataONE, and LTER [40] as well as Food Safety Knowledge Markup Language (FSK-ML) metadata [41] into data paper manuscripts.

The aim of the present article is to conceptualize the omics data paper, to create a specific article template for it, and to describe a prototype workflow for automated import of genomic metadata into an omics data paper manuscript. This workflow also accommodates the peer review and publication processes associated with the manuscript.

## Methods

### Approach

We took the following steps to approach the goal of establishing an omics data paper template and workflow:

1. Identify the high-level needs of the omics communities to better describe their datasets
2. Review existing standards, infrastructures [42], and datasets, as well as the existing data paper formats for describing (gen)omic data [43–45].
3. Synthesize the technical solutions and incorporate further functional needs to create the structure of the new type of data paper.

We created a dedicated data paper template, defining article sections and subsections to map the article narrative to metadata associated with the dataset(s) described in an omics data paper.

### Workflow for extracting relevant metadata from ENA XML files

We developed a workflow for automatic import of metadata into omics data paper manuscripts based on ENA's metadata structure, as well as the ArrayExpress [24] and BioSamples [25] databases. The workflow uses REST API requests and Xpath to retrieve segments of information from XML files from ENA, ArrayExpress, and BioSamples [46]. It then imports them into our proposed data paper manuscript structure, filling in the relevant subsections.

For demonstration, testing, and reproducibility purposes, this workflow was implemented in an R Shiny app [47–49], which visualizes metadata extracted from ENA inside the relevant sections of the proposed manuscript template within the application interface. The application also enables import of metadata into a valid JATS XML [50] document. Download of the HTML and XML versions of the metadata, as well as supporting supplementary material, is also enabled via reactive buttons in the user interface.

The R Shiny app was transformed into an installable R package using the golem framework [51], ensuring that it can be installed and run on any computer with R and RStudio. The package can be installed and run with just 3 R commands, which are documented in its Github repository along with all code, released under the Apache 2.0 license [52]. The R version at the time of developing the R Shiny app was R version 4.0.0 (2020–04–24) (Arbor Day) [49].

In addition, the R Shiny app can be run without installation as an interactive web app [53] deployed in an RStudio cloud environment [54] and hosted on a Shinyapps.io server [47, 48]. The code behind the interactive web app is openly available on Github [55] under the Apache 2.0 license.

### Integration of metadata extraction workflow with the ARPHA Writing Tool

After testing the metadata extraction and import workflow in the R shiny app, it was realized as a production-grade workflow integrated via Web service with the Pensoft ARPHA Writing Tool (AWT) [38]. The AWT is a web platform for collaborative authoring, reviewing, and publishing of manuscripts, developed and used by Pensoft in their publication process. It supports the creation of manuscripts by manual entry of text into different templates corresponding to separate article types, such as “Research Article," “Software Description," “Data Paper," and so forth. In addition, there are existing implemented workflows for import of metadata from files or web resources into the templates of some article types (e.g., “Data Paper" or “FSKX [Food Safety Knowledge] Paper") [38, 40]. After acceptance, the data papers consisting of peer-reviewed, corrected, and extended metadata descriptors are published under the Creative Commons CC-BY 4.0 license, which allows free and unlimited distribution and reuse given that the original source is credited. The datasets described in the data paper are made available under a license determined by the authors, which is specified in а dedicated “Usage rights" section of the data paper. Thus, we address 1 of the key aspects of the FAIR principles (R1.1) specifying that “(meta)data are released with a clear and accessible data usage license" to achieve data reusability [33].

Similarly, we established a new publication type, “OMICS Data Paper," and a manuscript template for it (see Additional File 1), following the proposed data paper structure. Essential sections of the omics data paper template were made mandatory in AWT such as the “Methods" section and the “Data resources" section. This means that the system requires the authors to fill them in before they can submit the manuscript for review.

We then replicated the genomic metadata import workflow from the R Shiny app inside the ARPHA Writing Tool. The workflow was designed to automatically populate some of the fields from the omics data paper manuscript template. It must be noted that not all fields from the template would be automatically filled in with metadata records by the workflow because ENA metadata records only cover a limited amount of information. For instance, sections such as “Environmental profile" and “Societal value" would not be populated and the users would have to manually fill them in with information if they wish to keep these sections in the data paper.

An important component of the design and implementation of the omics data papers is the BioSamples Supplementary Table. ENA metadata records that contain links to associated BioSamples metadata (MIxS checklists) [23, 25] are retrieved by the automatic import workflow and are transformed into a narrow format table, which will be attached to the manuscript as a comma-separated value (CSV) file. We restrict editing of Supplementary Tables imported from BioSamples to prevent metadata loss and tampering. Authors can only change the MIxS checklists related to their manuscript if they re-upload them to the long-term, trusted source repository: BioSamples. Synchronization with BioSamples from the manuscript in the ARPHA Writing Tool is enabled through a button labelled “Re-import from BioSamples."

## Findings

### Structure of the OMICS data paper

The omics data paper describes datasets generated in omics research. The described dataset is at the core of the data paper, but the methodology required to obtain it is just as valuable as the data itself. To guide authors in the authoring process and to better inform the readers about the contents of the proposed data paper, we designed a detailed manuscript template. Table 1 outlines each section and associated subsections of the template to be used either for manual population in the AWT or to match the metadata records extracted by the workflow to populate certain sections and subsections of the template. Many data paper sections do not have ENA metadata fields associated with them, and the authors are encouraged to fill in their contents in the data paper manuscript, as well as to update the original ENA record accordingly if possible.

**Table 1:** OMICS data paper sections, their purpose, and ENA metadata fields from which they are populated, if such fields exist

Article section	Purpose	ENA metadata source field
**Abstract**	Summary of the value of the study, the experimental design and the dataset itself	Study/Project XML://abstract
Introduction–Value of the dataset–Scientific value–Societal value	Outline of the reason for the study. Authors should put into perspective its value for the scientific and broader communities. Often sequencing studies are part of large-scale genome sequencing projects and this article section allows authors to explain their role in them	Written by the authors
Methods**–Sampling**–Environmental profile–Geographic range**-**Technologies used–Sample processing**–Technologies used**–Data processing	This section is split into 3 major parts to describe how the physical material was collected, processed, and transformed into a dataset.The “Sampling" section allows authors to outline the environmental and geographic characteristics of the locations where their material was collected. Sampling metadata imported from ENA fills in the “Sampling" section but the “Environmental profile" and “Geographic range" subsections remain to be filled in by the author manually. Authors are encouraged to share as much detail as they can (e.g., geographic coordinates, habitats, seasonal information). The sampling methods should be described in the “Technologies used" subsection. “Sample processing" should explain the laboratory procedures involved in the transition of the physical sample into its digital footprint. Finally, the “Data Processing" subsection should mention the steps taken to transform the raw dataset into the one that was published (e.g., normalization steps). None of the subsections are compulsory and the authors can write the Methods in a form outside these topics, but our template provides a detailed best-practices structure to follow	**ArrayExpress XML>Protocol XMLs:**protocol/typeprotocol/textprotocol/hardwareprotocol/softwareAnd**Experiment XMLs:**//EXPERIMENT/DESIGN/LIBRARY_DESCRIPTOR/LIBRARY_STRATEGYAnd**Experiment XMLs:**//EXPERIMENT/PLATFORM (→ Sample processing/Technologies used)And**Sample XMLs:**//SAMPLE/DESCRIPTION//SAMPLE/SAMPLE_ATTRIBUTES/SAMPLE_ATTRIBUTE
Biodiversity profile–Target–Taxonomic range–Functional range–Traits	This section describes the experimental design of the study. The target refers to the molecular target being studied (i.e., DNA, RNA, protein). The taxonomic range refers to the taxonomy of the studied organism(s) or the taxonomic composition of a metagenomic sample. The authors are encouraged to use a common taxonomy, but they can also provide their own during the authoring process in AWT. Authors can specify a particular range of biological functions that was the subject of their study (e.g., metabolic functions), as well as specific traits (e.g., pathogenicity) if relevant to the study	Written by the authors
**Data resources**	This is the section that contains a link to the dataset(s) (preferably to its permanent resolvable identifier, such as a DOI), as well as any accession numbers and data formats	**Study/Project XML:/**/XREF_LINK/ID[../DB = ‘ENA-FASTQ-FILES’]
Data statistics	Quantitative and qualitative description of the dataset (e.g., read depth, coverage, base ratios). This section helps readers to quickly evaluate the dataset by gauging some of its characteristics without having analysed the dataset themselves. Some of the data statistics can be represented as charts and/or short tables	Written by the authors
Caveats and limitations	A section to discuss what could be improved in the experiment, what future steps could be taken, and what to consider when reusing the published data	Written by the authors
Usage rights	Rights and licenses to use the data. The data paper is open access by default. Authors can read more about Pensoft's recommended data publishing licenses in [56]	Written by the authors
**Supplementary table**	Contains imported MIxS checklists for the imported BioSamples. The checklists are in long format. The table can be downloaded as a separate comma-separated value (CSV) file after publication	**Sample XMLs:**//SAMPLE/IDENTIFIERS/EXTERNAL_ID[@namespace = ‘BioSample’]And**BioSample XMLs:**//Property[@class]

The names of manuscript sections that could be automatically populated by the workflow are boldfaced in the first column. Values in the third column refer to the fields in ENA's XML files that contain the information used to automatically fill in the relevant section of the template. We have pointed to the type of XML (boldface) as well as the Xpath used to extract the information.

The template focuses on the value of the data, the methods used to generate it, and the qualitative and quantitative characteristics of the dataset. We have included a section to describe the biological entities that are the focus of the research: “Biodiversity profile." In addition to filing in the relevant subsections of this section, authors can attach a supplementary EML file or an Appendix table [57] to describe the different dimensions of the research target. Such Appendix tables can be used to record and link taxonomic, genomic, ecology, image, and other types of data using community-agreed vocabularies and ontologies. A spreadsheet template and instructions have been published as part of the Author's Guidelines of the *Biodiversity Data Journal* [58].

### Genomics metadata extraction workflow

Omics data papers can be created via 2 separate routes: (i) manually, by filling in all sections from the omics data paper template relevant to the research experiment inside the ARPHA Writing Tool; and (ii) semi-automatically, by using the genomics metadata extraction workflow with ENA metadata records and later manually enhancing the extracted metadata by filling in missing information inside the ARPHA Writing Tool. Here we outline the second route.

**Figure 2:**
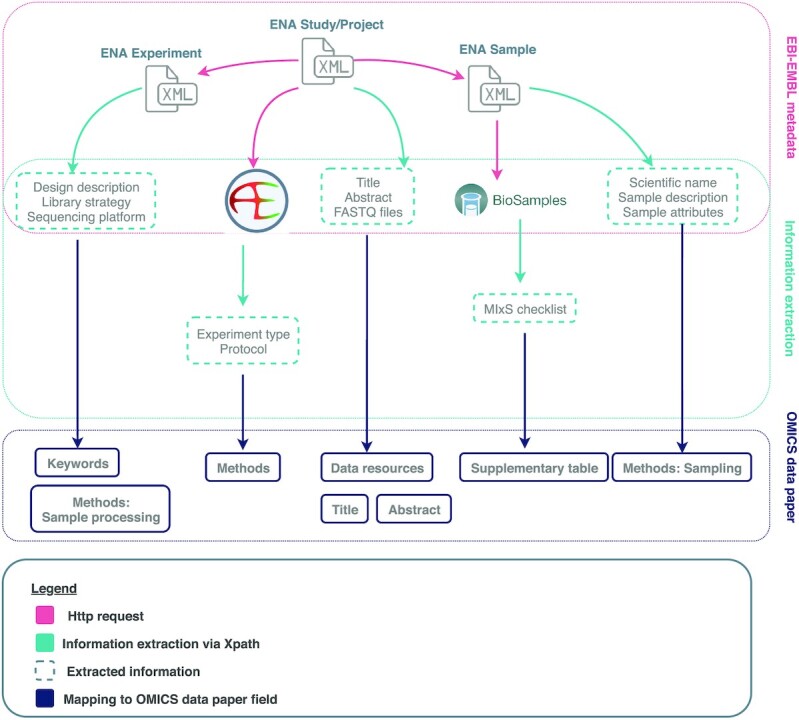
Metadata extraction workflow from ENA, ArrayExpress, and BioSamples

Metadata describing the datasets were used to facilitate creation and authoring of the data paper manuscript. By following ENA's metadata model [42], including its links to the ArrayExpress [24] and BioSamples [25] databases, we designed a workflow that orchestrates the extraction of relevant metadata from the various ENA XML files (Fig. 2). The Study XML and the Project XML are the starting points in the proposed workflow because they integrate all other types of data and metadata available in ENA for a given scientific study. Each metadata object in the ENA metadata model is associated with a unique identifier, which can be used to retrieve its corresponding XML file via the ENA API [42].

As outlined in our proposed workflow (Fig. 2), the Study or Project accession number is used to obtain an XML file that contains the accession numbers for all associated Experiment and Sample metadata objects, and in some cases ArrayExpress and BioSamples metadata objects.

ArrayExpress is a database storing data and metadata from functional genomic microarray or sequencing experiments [24]. ArrayExpress’s own submission platform Annotare and curators ensure that metadata from all sequencing experiments follow the Minimum Information About a Sequencing Experiment (MINSEQE) standard [24, 26].

Raw data from sequencing experiments submitted to ArrayExpress are also automatically deposited in ENA [46] as part of a Study metadata object, linked to Experiment and Sample objects [59]. Provenance of metadata imported from ArrayExpress can be established through a unique ArrayExpress accession number in the ENA Study XML. We integrated the extraction of curated, MINSEQE-compliant metadata from ArrayExpress into the workflow, thus enhancing manuscripts with additional metadata about experimental design and methodologies.

Another database within EMBL-EBI's infrastructure is BioSamples, a database that “stores and supplies descriptions and metadata about biological samples" [25]. Metadata descriptors in BioSamples records follow the MIxS standard [26]. Depending on the type of sample, submission to BioSamples requires different MIxS checklists to be filled in, after which they are publicly available in the form of XML files [25]. Unique identifiers link Sample XMLs from ENA with their associated BioSamples XML records. Thus, we are able to extract BioSamples information for any samples from a given ENA Study or Project. BioSamples records are imported into a table that is attached to the manuscript as a Supplementary CSV file. This supplementary table is a mandatory component of a manuscript, when accompanying BioSamples records are available, and cannot be removed by the authors. In cases when the authors spot a mistake in their submitted metadata, they are encouraged to change it within the BioSamples database. Upload of stand-alone BioSamples MIxS checklists is not permitted in the ARPHA Writing Tool, so that authors perform their corrections in the original metadata repository. After that, they can automatically retrieve them from BioSamples and import them into the manuscript with the click of a button. Thus, we promote the reuse and interoperability of MIxS-compliant metadata sourced from BioSamples.

**Figure 3:**
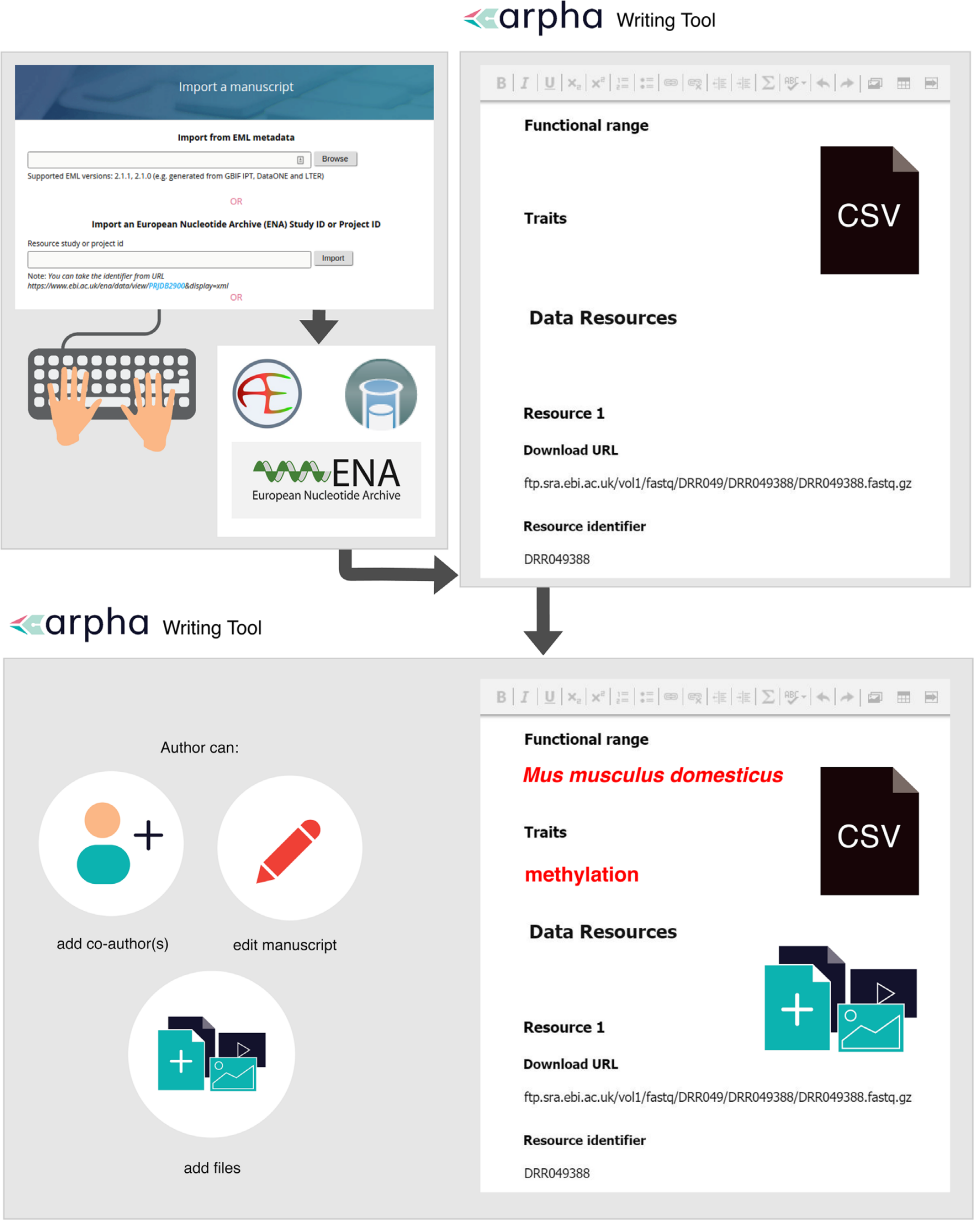
Automatic metadata import from ENA, ArrayExpress, and BioSamples facilitates the creation of omics data paper manuscripts inside the ARPHA Writing Tool.

We implemented the template and workflow into Pensoft's ARPHA Writing Tool [38], enabling import of the extracted ENA metadata records into the omics data paper template (Table 1). Fig. 3 shows a diagram demonstrating the import functionality from the perspective of the user.

### R shiny app—deployment and reproducibility

The template and workflow were first prototyped in an R shiny app [53], the code for which is open source and available on Github under Apache 2.0 license [55, 54], as outlined in the Methodology section of this article. The R shiny app is a web application emulating the functionality of the metadata import workflow in the ARPHA Writing Tool. The application runs in a virtual R environment [47, 54] and is deployed and hosted on the web via Shinyapps.io [48], configured to allow up to 50 concurrent connections.

The interface of the application features a text field for input of ENA Study or Project ID and a “Convert" button controlling the import of metadata and conversion to manuscript. Three buttons to download the outputs appear after the Convert button is clicked. The generated manuscript narrative, along with a data frame containing the BioSamples MIxS checklist, is visualized in the R shiny app interface. The narrative can be downloaded as an HTML file by clicking the “Download HTML" button, whereas the BioSamples checklist can be downloaded as a CSV file by clicking the “Download Supplementary Material" button. This CSV file is identical to the one generated as a supplementary file by the ARPHA Writing Tool.

The R shiny app has 1 additional functionality, which is not present in the workflow implemented in the ARPHA Writing Tool: it transforms the imported metadata into a Journal Article Tag Suite (JATS) XML file [50], which can be downloaded by clicking the “Download XML" button. We validated the XML against the latest JATS DTD version with the JATS4R validator [60]. The JATS XML is structured according to the Pensoft omics data paper template so that most article section nodes are defined with the sec tag and an attribute sec-type is used to define the exact section name (e.g., the Methods section is marked in the XML as <sec sec-type = ‘Methods’>). A basic “skeleton" file of the JATS XML file is available in the Github repository containing the code of the interactive web app [55].

Despite being tailored to the Pensoft omics data paper template, JATS XML files generated via the R shiny app can be used by other publishers or individuals to generate their own omics data paper manuscripts. Together with ENA's documentation about programmatic access to its resources [46], the codebase enables reproducibility of our workflow and creates the potential for it to be deployed by other journals or publishers.

## Discussion

### The data and metadata publishing landscape

The concept of data papers is not new; in fact, they have been in existence for >2 decades already. One of the first journals to implement this concept was Ecological Society of America's *Ecological Archives* [61, 62]. In 2011, Chavan and Penev envisioned metadata as a resource for authoring data papers for primary biodiversity data and identified a lack of clear guidelines and good practices for authoring metadata (the “how") and the incentives for authors to do so (the “why") [39]. They proposed data papers as a “mechanism to incentivise data publishing in biodiversity science" and introduced them to the biodiversity community through Pensoft's journals. To further simplify data paper authoring, Pensoft pioneered an integrated workflow for automatic metadata-to-manuscript conversion of primary biodiversity datasets published through GBIF's Integrated Publishing Toolkit (IPT) [37–63].

This streamlined metadata conversion workflow was first introduced in several of Pensoft's biodiversity journals and then in journals by other publishers, such as Nature's *Scientific Data, PLoS One, BMC Ecology*, and many others [64]. Since 2011, nearly 300 data papers have been published in Pensoft's journals and there is a steady uptake of this type of publication not only among Pensoft's journals but among journals of other publishers too [65]. Data papers are no longer an abstract idea but have already been practically implemented in multiple journals in different disciplines.

Since 2011, Pensoft has developed other integrative ways to streamline metadata authoring and data paper publication by integrating different workflows into its collaborative online authoring tool, AWT, and associated *Biodiversity Data Journal* [66]. For instance, metadata files following the GBIF EML profile used in the IPT can be directly converted and imported into manuscripts in AWT at the click of a button, then edited in the tool and submitted to the *Biodiversity Data Journal* [63, 67]. This workflow closely resembles the workflow described in this article but it is focused on ecological data. The EML workflow accepts a single specimen record identifier and imports information about that record from several infrastructures (GBIF, Barcode of Life Data Systems [BOLD], iDigBio, or PlutoF) into manuscripts [67]. It also enables conversion of an EML-formatted file into a biodiversity data paper [67], a functionality not covered by the present workflow, which only performs API requests.

Generation of extended metadata descriptors has been the focus of other tools, such as the Metadata Shiny Automated Resources and Knowledge (MetaShARK) [68] and Datascriptor [69], which is still under development. MetaShARK aims to facilitate assembly of ecology metadata by providing a user-friendly workflow for metadata packaging [68]. Unlike the workflow described here, it is more focused on primary metadata generation than metadata sharing and reuse [68]. Our workflow uses already generated metadata and provides a template for their extension to create an extended metadata description converted to narrative. Datascriptor is more closely related to our workflow because it aims to transform metadata, generated by following community standards, into a data article [69]. To do so, the developers have envisioned the generation of a JATS XML [69], which is what we have implemented in our R shiny app demonstrating the workflow for import of metadata into omics data paper manuscript.

### Data papers for the field of omics: rationale and benefits

Generation of omic data and metadata is one of the very first outputs of the research cycle, but not all of this is shared via research publications. Even when these data are published, the focus is usually on the interpretation of the data, rather than metadata quality or the FAIR properties of the dataset. Deposition of raw omic data, such as sequencing data, mass spectrometry proteomic data, and RNA-sequencing data, into centralized databases has become a routine practice for studies involving omic experiments [70]. ENA provides the necessary infrastructure to share sequencing data in a structured format and enables machine-readability and interoperability through the use of identifiers, consistent schema models, and APIs [42]. Describing those data, including its limitations and opportunities, inside a human-readable narrative will further improve their interpretation and reusability and increase their impact. With our proposed omics data paper and the automatic import prototype workflow, we encapsulate all metadata about a study into a single piece of narrative, thus completing the scientific process.

Authoring omics data papers, despite being aided by the automated workflow, requires additional effort and time largely because ENA records do not contain all metadata needed to assemble a thorough genomics study description. As the quality, breadth, and depth of metadata records in the sourced repositories improves, that additional effort will decrease. The prototype workflow merely demonstrates the possibility of interoperable metadata sharing and integration inside the publishing process. Here we outline some of the benefits that make the process of creating such manuscripts worthwhile, as well as how data interoperability contributes to the FAIR data and metadata publishing landscape.

1. *Omics data papers and underlying datasets undergo peer review and data auditing*

Prior to peer review of submitted omics data paper manuscripts, all underlying datasets go through mandatory data auditing, cleaning, and quality checks to ensure that they meet the journal standards for publication [71, 72]. This is done by a data auditor, whose role is to technically evaluate the submitted datasets for compliance to a data quality checklist [71] and to provide authors with a detailed report, including recommendations for improving the dataset. Only after the authors change the dataset according to the recommendations can it be approved for peer review. Peer reviewers review not only the narrative but also the datasets according to the Data Review Guidelines [73]. The introduction of data scientists into the publishing process ensures to an extent that submitted data and metadata are FAIR and consistent [72]. This double checking—first of the datasets by the data auditors and then of the whole manuscript by the reviewers—is a meticulous approach to enhancing the quality of datasets and to our knowledge has not been adopted by any other publisher so far.

2. *Publication of data papers improves metadata quality*

Authoring metadata is a necessary step to publish omics data into an open repository; however, there is considerable variability when it comes to the quality of the published metadata [6]. The workflow allows metadata authors, metadata standard creators, and data repository managers to evaluate the quality of metadata files deposited to INSDC databases. Throughout our testing phase we came across many datasets with missing or incorrectly formatted metadata fields. A recent observation by members of the Genomics Standards Consortium found that missing or incomplete metadata records from SARS-CoV-2 genomic and metagenomic studies are of frequent occurrence in INSDC databases and other repositories: such deficits of high-quality, community-standard metadata have become apparent during the COVID-19 healthcare crisis as global scientific efforts have been directed at generating and analysing data related to the novel coronavirus and data sharing and reuse have become crucial [6]. Currently, there is no workflow in place to feed information about such missing or incorrect records back to the INSDC databases, but we are optimistic about future integrative efforts that would help to streamline metadata between the INSDC repositories and other repositories or workflows. For example, such a feedback mechanism is one of the key deliverables of the EU-funded project BiCIKL starting in May 2021.

Some data repositories enforce in-house data or metadata curation practices. Curation of metadata, currently implemented by ArrayExpress via their Annotare tool [74, 75], is an adequate method for high-quality metadata publishing, based on standards. Most important, however, is that metadata authors learn to adopt and correctly use the existing standards in the process of describing their data. By directly observing the role of their metadata in creating the manuscript, they are made aware of its value and should be incentivized to improve the quality and quantity of the metadata that they provide. After importing metadata into their omics data paper manuscript, authors would need to manually correct and fill in the missing information, which defies the main purpose of the workflow: to make the data better described through extended and detailed metadata in the form of peer-reviewed, widely accessible and citable data papers.

3. *High-quality metadata enable data-driven discovery*

Metadata that follow community-accepted standards are vital for data-driven discoveries because they provide the necessary context to characterize the dataset that they describe. Omics data papers not only improve the quality of the metadata but also constitute an enhanced metadata record themselves.

After publication all data papers are publicly available also as JATS XML and submitted as such for archiving and display to PubMed Central [56], in addition to the traditional PDF and HTML formats. Therefore, all identifiers and links to external databases are marked up in the XML version of the article and can then be indexed by Web crawlers, including Pensoft's own RDFization scripts, which transform XML articles to Resource Description Framework (RDF) to ensure machine readability of the text [76]. Thus, the omics dataset metadata are indexed in several locations and become more findable and accessible.

The improved visibility of the omics datasets can open new possibilities for reuse of the datasets. For instance, in pharmaceutical science, old compounds are commonly researched as part of the development of new drugs because they could harbour unexplored biological activities [77]. By giving further visibility to omic datasets through their publication in an omics data paper, indexed in journal-focused databases and search engines, and by enhancing metadata through publication, we stimulate scientific research and data-driven discovery.

4. *Data papers help to establish priority*

Publishing data papers at early stages of the research process can provide an important benefit for authors: the opportunity to get the first scientific record for their effort in assembling a dataset and obtain feedback from the research community. It is well known that many authors are hesitant to publish datasets that they have not yet analysed or used for supporting any research findings for fear of someone else using the data and getting “scooped." By publishing a data paper, the authors are guaranteed that the described data can be reused in accordance with the Open Science principles, following all community-accepted ethical norms for citation, priority, and generating new knowledge through joint publications based on shared data.

5. *Publishing omics data papers is a way to obtain credit for one's work*

Science crediting further incentivizes researchers to publish omics data papers because their work impact can be measured in a way familiar to authors of traditional research papers, adding to their researcher impact metrics. In addition, the data managers and scientists who generate the data are not always among the authors of traditional research articles, which focus on the data analysis and outcomes. Thus, data paper publishing can be a way for all actors involved in the process of gathering, curating, and managing the data—be they early-stage researchers, technicians, or data scientists—to obtain credit for their valuable work.

### Limitations and future outlook

The automated workflow prototype for importing omics metadata into data paper manuscripts currently works only with ENA metadata records. While INSDC metadata are exchanged across all 3 databases in the consortium (ENA, GenBank, and DDBJ) [7], it would be beneficial if users could import metadata from any of the 3 data repositories via their associated identifier. The reason for the current limitation is the requirement for additional integrations, produced by the variation of APIs and the differing metadata schemas even between ENA, GenBank, and DDBJ repositories, which hold identical data and are synchronized. We decided to integrate the prototype workflow with ENA as the first showcase of this novel method of creation of data paper manuscripts because of the more straightforward links between ENA, BioSamples, and ArrayExpress compared to GenBank or DDBJ.

Currently, the streamlined metadata import workflow for the omics data paper is focused mostly on genomic data. In the future, we plan to expand the workflow to include other repositories and data types, such as metagenomics data and operational taxonomic unit tables. This addition will integrate new data science solutions for efficiently and interoperably exchanging and storing sparse and high-dimensional contingency tables along with their associated sample and taxonomic metadata (e.g., the BIOM format [78]). Thus, we support the development away from the fragmentation of data and towards a single quantum of information to exchange, containing interoperable, accessible, and transparent information. Making use of this advancement, future workflows for omics data paper creation may also support BIOM files for data provision, as outlined in an unpublished dissertation by Raïssa Meyer [79].

Integrations between existing infrastructures and data-driven initiatives are key to the FAIRness of data and metadata. The streamlined workflow for import of metadata from ENA, ArrayExpress, and BioSample is another step in this direction. However, to make metadata truly FAIR, there should be a 2-way link between the original data and metadata repository (e.g., ENA) and the enhanced metadata record (e.g., the omics data paper).

## Conclusions

The new omics data paper, implemented in Pensoft's publishing process, provides a mechanism for incentivizing omics data sharing and reuse through scholarly publishing. In addition, the workflow for import of metadata into manuscripts encourages and incentivizes authors to enhance data quality and completeness. The workflow also demonstrates the importance of linking data from different infrastructures using stable identifiers and thus sets an example for future integrations with other metadata and data repositories.

## Availability of Supporting Source Code and Requirements

• Project name: Omics Data Paper Generator

• Project home page: https://github.com/pensoft/omicsdatapaper

• Operating system(s): Platform independent

• Programming language: R

• Other requirements: R version 4.0.0 (2020–04–24) (Arbor Day), R Studio

• License: Apache 2.0

• RRID:  SCR_019809

• biotoolsID: biotools:omics-data-paper-shinyapp-golem

## Data Availability

There are no data set(s) supporting the results of this article. The availability of all code used in this research is listed in the section Availability of Supporting Source Code and Requirements. A snapshot of the code is also available in the *GigaScience* GigaDB repository [80].

## Additional Files

Additional File 1. HTML file demonstrating the structure of the omics data paper template in the ARPHA Writing Tool.

## Abbreviations

ABCD: Access to Biological Collections Data; API: Application Programming Interface; AWT: ARPHA Writing Tool; BOLD: Barcode of Life Data Systems; CSV: comma-separated value; DiSSCo: Distributed System of Scientific Collections; DwC: Darwin Core Standard; ENA: European Nucleotide Archive; FAIR: Findable, Accessible, Interoperable, and Reusable; FSK-M: Food Safety Knowledge Markup Language; GBIF: Global Biodiversity Information Facility; GeOMe: Genomic Observatories Metadatabase; GGBN: Global Genome Biodiversity Network; GMI: Global Microbial Identifier; GSC: Genomics Standards Consortium; iDigBio: Integrated Digitized Biocollections; INSDC: International Nucleotide Sequence Database Collaboration; IPT: Integrated Publishing Toolkit; JATS: Journal Article Tag Suite; LTER: Long Term Ecological Research Network; MetaShARK: Metadata Shiny Automated Resources and Knowledge; MIAME: Minimum Information About a Microarray Experiment; MINSEQE: Minimum Information about a high-throughput nucleotide SEQuencing Experiment; MIxS: Minimum Information about any (x) Sequence; OBIS: Ocean Biogeographic Information System.

## Competing Interests

M.D. is a PhD student at Pensoft through the EU-funded project IGNITE, and one of her key tasks during the PhD project is to develop methods for publication, dissemination, and reuse of biodiversity-related genomics data. T.G., G.Z., S.D., and L.P. are employed by Pensoft, and L.P. also holds a professorship position in the Bulgarian Academy of Sciences. Pensoft's *Biodiversity Data Journal* has implemented the genomics data paper workflow in its routine editorial practices. The authors declare that they have no other competing interests.

## Funding

This research has received funding from the European Union's Horizon 2020 research and innovation programme under the Marie Sklodowska-Curie grant agreement IGNITE (No. 764840) and from Pensoft Publishers.

## Authors' Contributions

M.D. performed the background research, designed the template and workflow, developed the workflow, R Shiny app and installable R package, wrote the original manuscript and reviewed the manuscript.R.M. designed the template and workflow, wrote the original manuscript and reviewed the manuscript.P.L.B. designed the template and workflow, wrote the original manuscript and reviewed the manuscript.T.G. supervised the implementation of the workflow into Pensoft's ARPHA Writing Tool.G.Z. implemented the workflow into Pensoft's ARPHA Writing Tool.S.D. implemented the workflow into Pensoft's ARPHA Writing Tool.V.S. wrote the original manuscript and reviewed the manuscript.L.P. developed the idea and conceptual framework, designed the template and workflow, supervised the implementation of the workflow into Pensoft's ARPHA Writing Tool, wrote the original manuscript and reviewed the manuscript.

## Supplementary Material

giab034_GIGA-D-20-00352_Original_Submission

giab034_GIGA-D-20-00352_Revision_1

giab034_Response_to_Reviewer_Comments_Original_Submission

giab034_Reviewer_1_Report_Original_Submission Philippe Rocca-Serra -- 1/4/2021 Reviewed

giab034_Supplemental_Files
