# Supplementary material for: A streamlined workflow for conversion, peer review, and publication of genomics metadata as omics data papers
Source: Gigascience. 2021 May 13;10(5):giab034. doi: 10.1093/gigascience/giab034 (PMC8117446; doi:10.1093/gigascience/giab034)
Supplement: giab034_GIGA-D-20-00352_Original_Submission [file giab034_giga-d-20-00352_original_submission.pdf]

GigaScience

A streamlined workflow for conversion, peer review and publication of genomics metadata as Omics Data Papers

--Manuscript Draft--

**Manuscript Number:**	GIGA-D-20-00352
**Full Title:**	A streamlined workflow for conversion, peer review and publication of genomics metadata as Omics Data Papers
**Article Type:**	Technical Note
**Funding Information:**	Pensoft Publishers — Mr Georgi Zhelezov, Mr Seyhan Demirov<br>H2020 Marie Skłodowska-Curie Actions (764840) — Ms Mariya Dimitrova
**Abstract:**	Background<br>Data papers have emerged as a powerful instrument for open data publishing, obtaining credit, and establishing priority for datasets generated in scientific experiments. Academic publishing improves data and metadata quality through peer-review and increases the impact of datasets by enhancing their visibility, accessibility, and re-usability.<br>Objective<br>We aimed to establish a new type of article structure and template for omics studies: the omics data paper. To improve data interoperability and further incentivise researchers to publish well-described data sets, we created a prototype workflow for streamlined import of genomics metadata from the European Nucleotide Archive directly into a data paper manuscript.<br>Methods<br>An omics data paper template was designed by defining key article sections which encourage the description of omics datasets and methodologies. A metadata import workflow, based on REpresentational State Transfer services and Xpath, was prototyped to extract information from the European Nucleotide Archive, ArrayExpress and BioSamples databases.<br>Findings<br>The template and workflow for automatic import of standard-compliant metadata into an omics data paper manuscript provide a mechanism for enhancing existing metadata through publishing.<br>Conclusion<br>The omics data paper structure and workflow for import of genomics metadata help to bring genomic and other omics datasets into the spotlight. Promoting enhanced metadata descriptions and enforcing manuscript peer review and data auditing of the underlying datasets brings additional quality to datasets. We hope that streamlined metadata re-use for scholarly publishing encourages authors to create enhanced metadata descriptions in the form of data papers to improve both the quality of their metadata and its findability and accessibility
**Corresponding Author:**	Mariya Dimitrova<br>Pensoft Publishers<br>Sofia, BULGARIA
**Corresponding Author Secondary Information:**	
**Corresponding Author's Institution:**	Pensoft Publishers
**Corresponding Author's Secondary Institution:**	
**First Author:**	Mariya Dimitrova
**First Author Secondary Information:**	
**Order of Authors:**	Mariya Dimitrova

Powered by Editorial Manager® and ProduXion Manager® from Aries Systems Corporation

	Raïssa Meyer
	Pier Luigi Buttigieg
	Teodor Georgiev
	Georgi Zhelezov
	Seyhan Demirov
	Vincent Smith
	Lyubomir Penev
**Order of Authors Secondary Information:**	
**Additional Information:**	
**Question**	**Response**
Are you submitting this manuscript to a special series or article collection?	No
**Experimental design and statistics**<br><br>Full details of the experimental design and statistical methods used should be given in the Methods section, as detailed in our Minimum Standards Reporting Checklist. Information essential to interpreting the data presented should be made available in the figure legends.<br><br>Have you included all the information requested in your manuscript?	Yes
**Resources**<br><br>A description of all resources used, including antibodies, cell lines, animals and software tools, with enough information to allow them to be uniquely identified, should be included in the Methods section. Authors are strongly encouraged to cite Research Resource Identifiers (RRIDs) for antibodies, model organisms and tools, where possible.<br><br>Have you included the information requested as detailed in our Minimum Standards Reporting Checklist?	Yes
**Availability of data and materials**	Yes

All datasets and code on which the conclusions of the paper rely must be either included in your submission or deposited in publicly available repositories (where available and ethically appropriate), referencing such data using a unique identifier in the references and in the "Availability of Data and Materials" section of your manuscript.<br><br>Have you have met the above requirement as detailed in our Minimum Standards Reporting Checklist?	

Manuscript

Click here to access/download;Manuscript;Dimitrova_et_al_Omics_Data_Pa

A streamlined workflow for conversion, peer review and publication of genomics metadata as Omics Data Papers

Mariya Dimitrova[1*], Raïssa Meyer[2], Pier Luigi Buttigieg[3], Teodor Georgiev[4], Georgi Zhelezov[5], Seyhan Demirov[6], Vincent Smith[7], Lyubomir Penev[8]

[1] Pensoft Publishers, Prof. Georgi Zlatarski Street 12, 1700 Sofia, Bulgaria;
Institute of Information and Communication Technologies, Bulgarian Academy of Sciences, Acad. G. Bonchev St., Block 25A, 1113 Sofia, Bulgaria
Correspondence address: Pensoft Publishers, Prof. Georgi Zlatarski Street 12, 1700 Sofia, Bulgaria
Email: m.dimitrova@pensoft.net
https://orcid.org/0000-0002-8083-6048
*Corresponding author

[2] Alfred-Wegener-Institut, Helmholtz-Zentrum für Polar- und Meeresforschung, Bremerhaven, Germany
Correspondence address: Alfred-Wegener-Institut, Helmholtz-Zentrum für Polar- und Meeresforschung, Bremerhaven, Germany
Email: raissa.meyer@awi.de
https://orcid.org/0000-0002-2996-719X

[3] Alfred-Wegener-Institut, Helmholtz-Zentrum für Polar- und Meeresforschung, Bremerhaven, Germany
Correspondence address: Alfred-Wegener-Institut, Helmholtz-Zentrum für Polar- und Meeresforschung, Bremerhaven, Germany
Email: pier.buttigieg@awi.de
https://orcid.org/0000-0002-4366-3088

[4] Pensoft Publishers, Prof. Georgi Zlatarski Street 12, 1700 Sofia, Bulgaria
Correspondence address: Pensoft Publishers, Prof. Georgi Zlatarski Street 12, 1700 Sofia, Bulgaria
Email: t.georgiev@pensoft.net
https://orcid.org/0000-0001-8558-6845

[5] Pensoft Publishers, Prof. Georgi Zlatarski Street 12, 1700 Sofia, Bulgaria
Correspondence address: Pensoft Publishers, Prof. Georgi Zlatarski Street 12, 1700 Sofia, Bulgaria
Email: g.zhelezov@pensoft.net

[6] Pensoft Publishers, Prof. Georgi Zlatarski Street 12, 1700 Sofia, Bulgaria
Correspondence address: Pensoft Publishers, Prof. Georgi Zlatarski Street 12, 1700 Sofia, Bulgaria
Email: programmer@pensoft.net

[7] The Natural History Museum, London, United Kingdom
Correspondence address: The Natural History Museum, London, United Kingdom
Email: vince@vsmith.info

https://orcid.org/0000-0001-5297-7452

[8] Pensoft Publishers, Prof. Georgi Zlatarski Street 12, 1700 Sofia, Bulgaria;
Institute of Biodiversity and Ecosystem Research, Bulgarian Academy of Sciences, 2 Gagarin Street, 1113 Sofia, Bulgaria
Correspondence address: Pensoft Publishers, Prof. Georgi Zlatarski Street 12, 1700 Sofia, Bulgaria
Email: l.penev@pensoft.net
https://orcid.org/0000-0002-2186-5033

Abstract

Background

Data papers have emerged as a powerful instrument for open data publishing, obtaining credit, and establishing priority for datasets generated in scientific experiments. Academic publishing improves data and metadata quality through peer-review and increases the impact of datasets by enhancing their visibility, accessibility, and re-usability.

Objective

We aimed to establish a new type of article structure and template for omics studies: the omics data paper. To improve data interoperability and further incentivise researchers to publish well-described data sets, we created a prototype workflow for streamlined import of genomics metadata from the European Nucleotide Archive directly into a data paper manuscript.

Methods

An omics data paper template was designed by defining key article sections which encourage the description of omics datasets and methodologies. A metadata import workflow, based on REpresentational State Transfer services and Xpath, was prototyped to extract information from the European Nucleotide Archive, ArrayExpress and BioSamples databases.

Findings

The template and workflow for automatic import of standard-compliant metadata into an omics data paper manuscript provide a mechanism for enhancing existing metadata through publishing.

Conclusion

The omics data paper structure and workflow for import of genomics metadata help to bring genomic and other omics datasets into the spotlight. Promoting enhanced metadata descriptions and enforcing manuscript peer review and data auditing of the underlying datasets brings additional quality to datasets. We hope that streamlined metadata re-use for scholarly publishing encourages authors to create enhanced metadata descriptions in the form of data papers to improve both the quality of their metadata and its findability and accessibility.

Keywords

data, data paper, omics, genomics, metadata, workflow, standards, FAIR principles, MIxS, MINSEQE

1. Introduction

The term "omics" refers to the study of biological systems through the examination of different elements of the molecular basis of life. For example, the genome is examined through the analysis of gene (DNA) sequences, the transcriptome is the collection of all mRNA molecules in an organism, and the metabolome is the collection of all metabolites and intermediate substrates participating in the metabolic pathways. Omic studies are generating large quantities of deeply minable data with increasing scale and complexity [1, 2]. Further, omics technologies and approaches have revolutionised biodiversity science [3, 4, 5].

Independently from the recent advances in omics technologies and data generation, however, the published omics biodiversity data and its accompanying, standardised

metadata, are still neither harmonised nor interoperable [6]. Existing infrastructures in omics data science focus on the sequence or molecular data generated from omics studies. The databases of the International Nucleotide Sequence Database Collaboration (INSDC) [7, 8] have provided a trusted archive for these data. In parallel, major infrastructures to handle higher-order biodiversity data (e.g. occurrences linked to taxa, specimen records) have emerged and include the Global Biodiversity Information Facility (GBIF) [9], the Integrated Digitized Biocollections (iDigBio) [10], the Distributed System of Scientific Collections (DiSSCo) [11], the Ocean Biogeographic Information System (OBIS) [12], the Global Genome Biodiversity Network (GGBN) [13], DataONE [14] and others. Some of these infrastructures support data repositories which follow community-accepted metadata standards. GBIF uses the Ecological Metadata Language (EML) standard for describing ecological datasets in XML files [15], whereas biodiversity data is recorded by following the Darwin Core Standard (DwC) [16, 17]. Likewise, the GGBN have developed their own GGBN Data Standard, which interoperates with DwC and the Access to Biological Collections Data (ABCD) schema for primary biodiversity data [18, 19, 20]. The INSDC cooperates with community standards initiatives such as the Genomic Standards Consortium (GSC) to implement their Minimum Information about any (x) Sequence (MIxS) checklists for genomic, metagenomic and environmental metadata descriptors, and with the Global Microbial Identifier (GMI) group for pathogen sequence metadata [21, 22]. MIxS consists of three checklists each containing several packages for the description of various environments where genomic material could be sampled from [22]. Other international data repositories such as EBI EMBL's ArrayExpress [23] and the BioSamples [24] database implement standards such as Minimum Information about a high-throughput nucleotide

SEQuencing Experiment (MINSEQE) and Minimum Information About a Microarray Experiment (MIAME) and various MIxS environmental checklists [25]. Databases such as the Genomic Observatories Metadatabase (GeOMe) [26] offer integrative solutions for the data management of genomic, geographical and ecological metadata by providing mechanisms to create standard-compliant templates tailored to specific use cases and linking metadata to dataset records via stable identifiers [27].

A more comprehensive approach towards omics metadata mobilisation is undertaken by the ISA Commons community [28], who use the “extensible, cross-domain format” ISA-Tab for organising metadata [29]. This format focuses on three major components of any scientific research: “Investigation”, “Study” and “Assay” to help structure the underlying study and assay specific metadata records [29]. Software for creating and validating ISA-Tab files has also been developed as part of the ISA Tools framework [29]. This framework aims to complement existing omics standards to improve the description of research outputs in the field of omics. Several omics data repositories, such as EMBL-EBL’s Metabolights [30] and GigaScience’s GigaDB [31], have adopted the ISA model and serialisations.

There are different ways scientists can publish their data in a FAIR (Findable, Accessible, Interoperable and Re-usable) [32, 33] manner, however, all can be attributed to two main routes: (1) data publishing through international trusted data repositories, such as INSDC [7], GBIF [9], and others, and (2) scholarly data publishing in the form of data papers or as data underpinning a research article [34, 35, 36, 37, 38, 39]. While the first route focuses on data aggregation, standardisation and re-use, the second one augments the quality and reusability of data and metadata through

peer reviewing and data auditing in the scholarly publishing process. Scholarly data publishing provides an opportunity to enhance the original metadata in the data paper narrative and to link it to the original dataset via stable identifiers, thus improving the reproducibility and findability of the data [34]. Furthermore, it ensures a scientific record, crediting and acknowledgement for the data creators and scientists in the form of citable scholarly articles. Academic publishing involves dissemination of research through additional channels, such as journal distribution networks, and creates increased opportunities for open science collaboration [34].

While standards and infrastructures are crucial to the advancement of data sharing and reuse within the field of omics, we argue that incentivising authors to publish their data in the form of peer reviewed journal articles (data papers) creates the driving force towards a truly FAIR data world (Fig. 1).

Fig. 1. The different layers of FAIRness of data and metadata. Describing data and metadata in a data paper publication helps to enhance their FAIRness through provision of better visibility and accessibility.

As more and more researchers want to deposit and share their datasets, standards, infrastructures, and workflows become central to delivering FAIR data. Following the example set by Chavan and Penev [34], who introduced data papers in biodiversity science, we have established a concept for an omics data paper - a type of scholarly paper in which data, generated in genomic or other omic experiments, is described with extended and peer reviewed metadata, and linked to the corresponding dataset(s)

deposited in an INSDC or other archive. To further incentivise authors to publish omics data papers and to demonstrate the importance of high-quality metadata, we propose a prototype of a streamlined workflow for conversion of European Nucleotide Archive (ENA) genomic metadata directly into a data paper manuscript. We build upon previous work by Pensoft, namely workflows for automatic import of EML metadata from GBIF, DataONE and LTER [40] as well as Food Safety Knowledge Markup Language (FSK-ML) metadata [41] into data paper manuscripts.

The aim of the present paper is to conceptualise the omics data paper, to create a specific article template for it, and to describe a prototyped workflow for automated import of genomic metadata into an omics data paper manuscript. This workflow also accommodates the peer review and publication processes associated with the manuscript.

2. Methods

Approach

We took the following steps to approach the goal of establishing an omics data paper template and workflow:

1. Identify the high-level needs of the omics communities to better describe their datasets
2. Review existing standards, infrastructures [42] and datasets, as well as the existing data paper formats for describing (gen)omic data [43, 44, 45].
3. Synthesise the technical solutions and incorporate further functional needs to create the structure of the new type of data paper.

We created a template, defining article sections and subsections to map the article narrative to metadata associated with the dataset(s) described in an omics data paper.

Workflow for extracting relevant metadata from ENA XML files

We developed a workflow for automatic import of metadata into omics data paper manuscripts based on ENA's metadata structure, as well as the ArrayExpress [23] and BioSamples [24] databases. The workflow uses REST API requests and Xpath to retrieve segments of information from XML files from ENA, ArrayExpress and BioSamples [46]. It then imports them into our proposed data paper manuscript structure, filling in the relevant subsections.

For demonstration, testing and reproducibility purposes, this workflow was implemented in a R Shiny app [47, 48, 49] which visualises metadata extracted from ENA inside the relevant sections of the proposed manuscript template within the application interface. The application also enables import of metadata into a valid JATS XML [50] document. Download of the HTML and XML versions of the metadata, as well as supporting supplementary material is also enabled via reactive buttons in the user interface.

The R Shiny app was transformed into an installable R package using the golem framework [51], ensuring that it can be installed and run on any computer with R and RStudio. The package can be installed and run with just three R commands, which are documented in its Github repository [52]. The R version at the time of developing the R Shiny app was R version 4.0.0 (2020-04-24) (Arbor Day) [49].

In addition, the R Shiny app can be run without installation as an interactive web app [53] deployed in an RStudio cloud environment [55] and hosted on a Shinyapps.io

server [47, 48]. The code behind the interactive web app is openly available on Github [54].

Integration of metadata extraction workflow with the ARPHA Writing Tool

After testing the metadata extraction and import workflow in the R shiny app, it was realised as a production-grade workflow integrated via Web service with the Pensoft's ARPHA Writing Tool (AWT) [39]. The AWT is a web platform for collaborative authoring, reviewing and publishing of manuscripts, developed and used by Pensoft in their publication process. It supports the creation of manuscripts by manual entry of text into different templates corresponding to separate article types, such as "Research Article", "Software Description", "Data Paper", etc. In addition, there are existing implemented workflows for import of metadata from files or web resources into the templates of some article types (e.g. "Data Paper" or "FSKX (Food Safety Knowledge) Paper") [39, 40].

Similarly, we established a new publication type, "OMICS Data Paper", and a manuscript template for it, following the proposed data paper structure. Essential sections of the omics data paper template were made mandatory in AWT such as the "Methods" section and the "Data resources" section. This means that the system requires the authors to fill them in before they can submit the manuscript for review.

We then replicated the genomic metadata import workflow from the R Shiny app inside the ARPHA Writing Tool. The workflow was designed to automatically populate some of the fields from the omics data paper manuscript template. It must be noted that not all fields from the template would be automatically filled in with metadata records by the workflow because ENA metadata records only cover a limited amount of

information. For instance, sections such as “Environmental profile” and “Societal value” would not be populated and the users would have to manually fill them in with information, if they wish to keep these sections in the data paper.

An important component of the design and implementation of the omics data papers is the BioSamples Supplementary Table. ENA metadata records that contain links to associated BioSamples metadata (MIxS checklists) [22, 24] are retrieved by the automatic import workflow, and are transformed into a narrow format table, which will be attached to the manuscript as a comma-separated value (CSV) file. We restrict editing of Supplementary Tables imported from BioSamples to prevent metadata loss and tampering. Authors can only change the MIxS checklists related to their manuscript if they re-upload them to the long-term, trusted source repository: BioSamples. Synchronisation with BioSamples from the manuscript in the ARPHA Writing Tool is enabled through a button labelled “Re-import from BioSamples”.

3. Findings

Structure of the OMICS data paper

The omics data paper describes datasets generated in omics research. The described dataset is at the core of the data paper, but the methodology required to obtain it is just as valuable as the data itself. To guide authors in the authoring process and to better inform the readers about the contents of the proposed data paper, we designed a detailed manuscript template. Table 1 outlines each section and associated subsections of the template to be used either for manual population in the AWT, or to match the metadata records extracted by the workflow to populate certain sections

and subsections of the template. Many data paper sections do not have ENA metadata fields associated with them and the authors are encouraged to fill in their contents in the data paper manuscript as well as to update the original ENA record accordingly if possible.

**Article section**	**Purpose**	**ENA metadata source field**
**Abstract**	Summary of the value of the study, the experimental design and the dataset itself.	**Study/Project XML:**<br>//abstract
Introduction<br>- Value of the dataset<br>- Scientific value<br>- Societal value	Outline of the reason for the study. Authors should put into perspective its value for the scientific and broader communities. Often sequencing studies are part of large scale genome sequencing projects and this article section allows authors to explain their role in them.	*Written by the authors*
Methods<br>- **Sampling**<br>- Environmental profile<br>- Geographic range<br>- Technologies used<br>- Sample processing<br>- **Technologies used**<br>- Data processing	This section is split into 3 major parts to describe how the physical material was collected, processed and transformed into a dataset.<br>The "Sampling" section allows authors to outline the environmental and geographic characteristics of the locations where their material was collected. Sampling metadata imported from ENA fills in the "Sampling" section but the "Environmental profile" and "Geographic range" subsections remain to be	**ArrayExpress XML> Protocol XMLs:**<br>protocol/type<br>protocol/text<br>protocol/hardware<br>protocol/software<br><br>And<br><br>**Experiment XMLs:**<br>//EXPERIMENT/DESIGN/LIBRARY_DESCRIPTOR/LIBRARY_STRATEGY<br><br>And<br><br>**Experiment XMLs:**<br>//EXPERIMENT/PLATFORM (-> Sample

	filled in by the author manually. Authors are encouraged to share as much detail as they can (e.g. geographic coordinates, habitats, seasonal information, etc.). The sampling methods should be described in the “Technologies used” subsection.<br>“Sample processing” should explain the laboratory procedures involved in the transition of the physical sample into its digital footprint. Finally, the “Data Processing” subsection should mention the steps taken to transform the raw dataset into the one which was published (e.g. normalisation steps).<br>None of the subsections are compulsory and the authors can write the Methods in a form outside these topics but our template provides a detailed best practices structure to follow.	processing/Technologies used)<br><br>And<br><br>**Sample XMLs:**<br><br>//SAMPLE/DESCRIPTION<br>//SAMPLE/SAMPLE_ATTRIBUTES/SAMPLE_ATTRIBUTE
Biodiversity profile<br>- Target<br>- Taxonomic range<br>- Functional range<br>- Traits	This section describes the experimental design of the study. The target refers to the molecular target being studied (i.e. DNA, RNA, protein). The taxonomic range refers to the taxonomy of the studied organism(s) or the taxonomic composition of a metagenomic sample. The authors are encouraged to use a common taxonomy but they can also provide their	*Written by the authors*

	own during the authoring process in AWT. Authors can specify a particular range of biological functions which was the subject of their study (e.g. metabolic functions), as well as specific traits (e.g. pathogenicity) if relevant to the study.	
**Data resources**	This is the section which contains a link to the dataset(s) (preferably to its permanent resolvable identifier, such as a DOI), as well as any accession numbers and data formats.	**Study/Project XML:** //XREF_LINK/ID[../DB='ENA-FASTQ-FILES']
Data statistics	Quantitative and qualitative description of the dataset. (e.g. read depth, coverage, base ratios). This section helps readers to quickly evaluate the dataset by gauging some of its characteristics without having analysed the dataset themselves. Some of the data statistics can be represented as charts and/or short tables.	*Written by the authors*
Caveats and limitations	A section to discuss what could be improved in the experiment, what future steps could be taken and what to consider when re-using the published data.	*Written by the authors*
Usage rights	Rights and licenses to use the data. The data paper is open access by default Authors can read more about Pensoft's recommended data publishing licenses in [38]	*Written by the authors*

**Supplementary table**	Contains imported MIxS checklists for the imported BioSamples. The checklists are in long format. The table can be downloaded as a separate comma-separated value (CSV) file after publication.	**Sample XMLs:** //SAMPLE/IDENTIFIERS/ EXTERNAL_ID[@namespace="BioSample"]<br><br>And<br><br>**BioSample XMLs:** //Property[@class]

Table 1. OMICS data paper sections, their purpose and ENA metadata fields from which they are populated, if such fields exist. The names of manuscript sections which could be automatically populated by the workflow are marked in bold in the first column. Values in the third column refer to the fields in ENA's XML files which contain the information used to automatically fill in the relevant section of the template. We have pointed to the type of XML (marked in bold) as well as the Xpath used to extract the information.

The template focuses on the value of the data, the methods used to generate it and the qualitative and quantitative characteristics of the dataset. We have included a section to describe the biological entities which are the focus of the research: "Biodiversity profile". In addition to filing in the relevant subsections of this section, authors can attach a supplementary EML file or an Appendix table [56] to describe the different dimensions of the research target. Such Appendix tables can be used to record and link taxonomic, genomic, ecology, image and other types of data using community agreed vocabularies and ontologies. A spreadsheet template and instructions have been published as part of the Author's Guidelines of the Biodiversity Data Journal (BDJ) [57].

Genomics metadata extraction workflow

Omics data papers can be created via two separate routes: 1) manually, by filling in all sections from the omics data paper template relevant to the research experiment inside the ARPHA Writing Tool and 2) semi-automatically, by using the genomics metadata extraction workflow with ENA metadata records and later manually enhancing the extracted metadata by filling in missing information inside the ARPHA Writing Tool. Here we outline the second route.

Metadata describing the datasets was utilised to facilitate creation and authoring of the data paper manuscript. By following ENA's metadata model [42], including its links to the ArrayExpress [23] and BioSamples [24] databases, we designed a workflow which orchestrates the extraction of relevant metadata from the various ENA XML files (Fig. 2). The Study XML and the Project XML are the starting points in the proposed workflow as they integrate all other types of data and metadata available in ENA for a given scientific study. Each metadata object in the ENA metadata model is associated with a unique identifier, which can be used to retrieve its corresponding XML file via the ENA API [42].

Fig. 2 Metadata extraction workflow from ENA, ArrayExpress and BioSamples

As outlined in our proposed workflow (Fig. 2), the Study or Project accession number is used to obtain a XML file which contains the accession numbers for all associated Experiment and Sample metadata objects, and in some cases ArrayExpress and BioSamples metadata objects.

ArrayExpress is a database storing data and metadata from functional genomic microarray or sequencing experiments [23]. ArrayExpress' own submission platform Annotare and curators ensure that metadata from all sequencing experiments follow the Minimum Information About a Sequencing Experiment (MINSEQE) standard [23, 25].

Raw data from sequencing experiments submitted to ArrayExpress are also automatically deposited in ENA [46] as part of a Study metadata object, linked to Experiment and Sample objects [58]. Provenance of metadata imported from ArrayExpress can be established through a unique ArrayExpress accession number in the ENA Study XML. We integrated the extraction of curated, MINSEQE compliant metadata from ArrayExpress into the workflow, thus enhancing manuscripts with additional metadata about experimental design and methodologies.

Another database within EMBL-EBI's infrastructure is BioSamples, a database which "stores and supplies descriptions and metadata about biological samples" [24]. Metadata descriptors in BioSamples records follow the MIxS standard [25]. Depending on the type of sample, submission to BioSamples requires different MIxS checklists to be filled in, after which they are publicly available in the form of XML files [24]. Unique identifiers link Sample XMLs from ENA with their associated BioSamples XML records. Thus, we are able to extract BioSamples information for any samples from a given ENA Study or Project. BioSamples records are imported into a table which is attached to the manuscript as a Supplementary CSV file. This supplementary table is a mandatory component of a manuscript, when accompanying BioSamples records are

available, and cannot be removed by the authors. In cases when the authors spot a mistake in their submitted metadata, they are encouraged to change it within the BioSamples database. Upload of standalone BioSamples MIxS checklists is not permitted in the ARPHA Writing Tool, so that authors perform their corrections in the original metadata repository. After that, they can automatically retrieve them from BioSamples and import them into the manuscript with the click of a button. Thus, we promote the reuse and interoperability of MIxS compliant metadata sourced from BioSamples.

We implemented the template and workflow into Pensoft's ARPHA Writing Tool [39] , enabling import of the extracted ENA metadata records into the omics data paper template (Table 1). Fig. 3 shows a diagram demonstrating the import functionality from the perspective of the user.

Fig. 3. Automatic metadata import from ENA, ArrayExpress and BioSamples, facilitates the creation of omics data paper manuscripts inside the ARPHA Writing Tool.

R shiny app - deployment and reproducibility

The template and workflow were first prototyped in a R shiny app [53], the code for which is open source and available on Github under Apache 2.0 license [54, 55], as outlined in the Methodology section of this paper. The R shiny app is a web application emulating the functionality of the metadata import workflow in the ARPHA Writing Tool. The application runs in a virtual R environment [47, 55] and is deployed and hosted on the web via Shinyapps.io [48], configured to allow up to 50 concurrent connections.

The interface of the application features a text field for input of ENA Study or Project ID and an 'Convert' button controlling the import of metadata and conversion to manuscript. Three buttons to download the outputs appear after the 'Convert' button is clicked. The generated manuscript narrative, along with a data frame containing the BioSamples MIxS checklist, are visualised in the R shiny app interface. The narrative can be downloaded as a HTML file by clicking the 'Download HTML' button, whereas the BioSamples checklist can be downloaded as a CSV file by clicking the 'Download Supplementary Material' button. This CSV file is identical to the one generated as a supplementary file by the ARPHA Writing Tool.

The R shiny app has one additional functionality, which is not present in the workflow implemented in the ARPHA Writing Tool: it transforms the imported metadata into a Journal Article Tag Suite (JATS) XML file [50], which can be downloaded by clicking the 'Download XML' button. We validated the XML against the latest JATS DTD version with the JATS4R validator [59]. The JATS XML is structured according to the Pensoft omics data paper template so that most article section nodes are defined with the sec tag and an attribute sec-type is used to define the exact section name (e.g. the Methods section is marked in the XML as <sec sec-type="Methods">). A basic "skeleton" file of the JATS XML file is available in the Github repository containing the code of the interactive web app [54].

Despite being tailored to the Pensoft omics data paper template, JATS XML files generated via the R shiny app can be used by other publishers or individuals to generate their own omics data paper manuscripts. Together with ENA's documentation about programmatic access to its resources [46], the codebase enables reproducibility of our workflow and creates the potential for it to be deployed by other journals or publishers.

4. Discussion

The data and metadata publishing landscape

The concept of data papers is not new; in fact, they have been in existence for more than two decades already. One of the first journals to implement this concept was Ecological Society of America's Ecological Archives [60, 61]. In 2011, Chavan and Penev envisioned metadata as a resource for authoring data papers for primary biodiversity data and identified a lack of clear guidelines and good practices for authoring metadata (the "how") and the incentives for authors to do so (the "why") [34]. They proposed data papers as a "mechanism to incentivise data publishing in biodiversity science" and introduced them to the biodiversity community through Pensoft's journals. To further simplify data paper authoring, Pensoft pioneered an integrated workflow for automatic metadata-to-manuscript conversion of primary biodiversity datasets published through GBIF's Integrated Publishing Toolkit (IPT) [34, 37, 39, 40, 62].

This streamlined metadata conversion workflow was first introduced in several of Pensoft's biodiversity journals and then in journals by other publishers, such as Nature's Scientific Data, PLOS ONE, BMC Ecology and many others [63]. Since 2011, nearly 300 data papers have been published in Pensoft's journals and there is a steady uptake of this type of publication not only among Pensoft's journals but among journals of other publishers too [64]. Data papers are no longer an abstract idea but have already been practically implemented in multiple journals in different disciplines.

Since 2011, Pensoft has developed other integrative ways to streamline metadata authoring and data paper publication by integrating different workflows into its collaborative online authoring tool, the ARPHA Writing Tool (AWT) and associated Biodiversity Data Journal [65]. For instance, metadata files following the GBIF EML profile used in the IPT can be directly converted and imported into manuscripts in AWT "at the click of a button", then edited in the tool and submitted to the Biodiversity Data Journal [62, 66]. This workflow closely resembles the workflow described in this paper but it is focused on ecological data. The EML workflow accepts a single specimen record identifier and imports information about that record from several infrastructures (GBIF, Barcode of Life Data Systems (BOLD), iDigBio, or PlutoF) into manuscripts [66]. It also enables conversion of an EML-formatted file into a biodiversity data paper [66], a functionality not covered by the present workflow, which only performs API requests.

Generation of extended metadata descriptors has been the focus of other tools, such as the Metadata Shiny Automated Resources and Knowledge (MetaShARK) [67] and Datascriptor [68], which is still under development. MetaShARK aims to facilitate assembly of ecology metadata by providing a user-friendly workflow for metadata packaging [67]. Unlike the workflow described here, it is more focused on primary metadata generation than metadata sharing and reuse [67]. Our workflow uses already generated metadata and provides a template for their extension to create an extended metadata description converted to narrative. Datascriptor is more closely related to our workflow because it aims to transform metadata, generated by following community standards, into a data article [68]. To do so, the developers have envisioned the generation of a JATS XML [68], which is what we have implemented in

our R shiny app demonstrating the workflow for import of metadata into omics data paper manuscript.

Data papers for the field of omics: rationale and benefits

Generation of omic data and metadata is one of the very first outputs of the research cycle, but not all of this is shared via research publications. Even when these data are published, the focus is usually on the interpretation of the data, rather than metadata quality or the FAIR properties of the dataset. Deposition of raw omic data, such as sequencing data, mass spectrometry (MS) proteomic data and RNA-sequencing data, into centralised databases has become a routine practice for studies involving omic experiments [69]. ENA provides the necessary infrastructure to share sequencing data in a structured format and enables machine-readability and interoperability through the use of identifiers, consistent schema models and APIs [42]. Describing that data, including its limitations and opportunities, inside a human readable narrative will further improve its interpretation and reusability, and increase its impact. With our proposed omics data paper and the automatic import prototype workflow, we encapsulate all metadata about a study into a single piece of narrative, thus completing the scientific process.

Authoring omics data papers, despite being aided by the automated workflow, requires additional effort and time largely because ENA records do not contain all metadata needed to assemble a thorough genomics study description. As the quality, breadth, and depth of metadata records in the sourced repositories improves, that additional

effort will decrease. The prototype workflow merely demonstrates the possibility of interoperable metadata sharing and integration inside the publishing process. Here we outline some of the benefits which make the process of creating such manuscripts worthwhile, as well as on how data interoperability contributes to the FAIR data and metadata publishing landscape.

1. *Omics data papers and underlying datasets undergo peer-review and data auditing*

   Prior to peer-review of submitted omics data paper manuscripts, all underlying datasets go through mandatory data auditing, cleaning and quality checks to assure that they meet the journal standards for publication [70]. This is done by a data auditor, whose role is to technically evaluate the submitted datasets for compliance to a data quality checklist [70] and to provide authors with a detailed report, including recommendations for improving the dataset. Only after the authors change the dataset according to the recommendations, it can be approved for peer-review. The introduction of data scientists into the publishing process ensures to an extent that submitted data and metadata are FAIR and consistent. This double checking - first of the datasets by the data auditors and then of the whole manuscript by the reviewers - is an meticulous approach to enhancing the quality of datasets and to the best of our knowledge has not been adopted by any other publisher so far.

2. *Publication of data papers improves metadata quality*

Authoring metadata is a necessary step to publish omics data into an open repository; however, there is considerable variability when it comes to the quality of the published metadata [6]. The workflow allows metadata authors, metadata standard creators and data repository managers to evaluate the quality of metadata files deposited to INSDC databases. Throughout our testing phase we came across many datasets with missing or incorrectly formatted metadata fields. A recent observation by members of the Genomics Standards Consortium found that missing or incomplete metadata records from SARS-CoV-2 genomic and metagenomic studies are of frequent occurrence in INSDC databases and other repositories: such deficits of high-quality, community-standard metadata, have become apparent during the COVID-19 healthcare crisis as global scientific efforts have been directed at generating and analysing data related to the novel coronavirus and data sharing and re-use have become crucial [6].

Curation of metadata, currently implemented by ArrayExpress via their Annotare tool [71, 72], is an adequate method for high-quality metadata publishing, based on standards. Most important, however, is that metadata authors learn to adopt and correctly use the existing standards in the process of describing their data. By directly observing the role of their metadata in creating the manuscript, they are made aware of its value and should be incentivised to improve the quality and quantity of the metadata they provide. After importing metadata into their omics data paper manuscript, authors would need to manually correct and fill in the missing information, which defies the main purpose of the workflow: to make the data better described through

extended and detailed metadata in the form of peer-reviewed, widely accessible and citable data papers.

3. *High-quality metadata enables data-driven discovery*

Metadata which follows community accepted standards is vital for data-driven discoveries as it provides the necessary context to characterise the dataset it describes. Omics data papers do not only improve the quality of the metadata but also constitute an enhanced metadata record themselves. As a result, the publication can inspire new research ideas and open new possibilities for use of the dataset. For instance, in pharmaceutical science, old compounds are commonly researched as part of the development of new drugs because they could harbour unexplored biological activities [73]. By giving further visibility to omic datasets through their publication in an omics data paper and by enhancing metadata through publication, we stimulate scientific research and data-driven discovery.

4. *Data papers help to establish priority*

Publishing data papers at early stages of the research process can provide an important benefit for authors: the opportunity to get the first scientific record for their effort in assembling a dataset and obtain feedback from the research community. It is well known that many authors are hesitant to publish datasets which they have not yet analysed or used for supporting any research findings for fear of someone else using the data and getting ‘scooped’. By publishing a

data paper, the authors are guaranteed that the described data can be re-used in accordance with the Open Science principles, following all community accepted ethical norms for citation, priority and generating new knowledge through joint publications based on shared data.

5. *Publishing omics data papers is a way to obtain credit for one's work*

Science crediting further incentivises researchers to publish omics data papers because their work impact can be measured in a way familiar to authors of traditional research papers, adding to their researcher impact metrics. In addition, the data managers and scientists who generate the data are not always among the authors of traditional research articles, which focus on the data analysis and outcomes. Thus, data paper publishing can be a way for all actors involved in the process of gathering, curating and managing the data - be they early stage researchers, technicians or data scientists - to obtain credit for their valuable work.

Limitations and future outlooks

The automated workflow prototype for importing omics metadata into data paper manuscripts currently works only with ENA metadata records. While INSDC metadata is exchanged across all three databases in the consortium (ENA, GenBank and DDBJ) [7], it would be beneficial if users could import metadata from any of the three data repositories via their associated identifier. The reason for the current limitation is the requirement for additional integrations, produced by the variation of APIs and the

differing metadata schemas even between ENA, GenBank and DDBJ repositories which hold identical data and are synchronised. We decided to integrate the prototype workflow with ENA as the first showcase of this novel method of creation of data paper manuscripts because of the more straightforward links between ENA, BioSamples and ArrayExpress compared to GenBank or DDBJ.

Currently, the streamlined metadata import workflow for the omics data paper is focused mostly on genomic data. In the future, we plan to expand the workflow to include other repositories and data types, such as metagenomics data and operational taxonomic units (OTU) tables. This addition will integrate new data science solutions for efficiently and interoperably exchanging and storing sparse and high dimensional contingency tables along with their associated sample and taxonomic metadata (e.g. the BIOM format [74]). Thus, we support the development away from the fragmentation of data and towards a single quantum of information to exchange, containing interoperable, accessible, and transparent information. Making use of this advancement, future workflows for omics data paper creation may also support BIOM files for data provision, as outlined in an unpublished dissertation by Raïssa Meyer (2020).

Integrations between existing infrastructures and data-driven initiatives are key to the FAIRness of data and metadata. The streamlined workflow for import of metadata from ENA, ArrayExpress and BioSample is another step in this direction. However, to make metadata truly FAIR, there should be a two-way link between the original data and

metadata repository (e.g. ENA) and the enhanced metadata record (e.g. the omics data paper).

5. Conclusions

In conclusion, the new omics data paper, implemented in Pensoft's publishing process provides a mechanism for incentivising omics data sharing and reuse through scholarly publishing. In addition, the workflow for import of metadata into manuscripts encourages and incentivises authors to enhance data quality and completeness. The workflow also demonstrates the importance of linking data from different infrastructures using stable identifiers and thus sets an example for future integrations with other metadata and data repositories.

Availability of supporting source code and requirements

- Project name: omicsdatapaper
- Project home page: https://github.com/pensoft/omicsdatapaper
- Operating system(s): Platform independent
- Programming language: R
- Other requirements: R version 4.0.0 (2020-04-24) (Arbor Day), R Studio
- License: Apache 2.0

List of abbreviations

ABCD standard: Access to Biological Collections Data standard; AWT: ARPHA Writing Tool; BOLD: Barcode of Life Data Systems; CSV file: comma-separated value file; DiSSCo: Distributed System of Scientific Collections; DwC: Darwin Core Standard; ENA: European Nucleotide Archive; FAIR data: Findable, Accessible,

Interoperable and Re-usable data; GBIF: Global Biodiversity Information Facility; GGBN: Global Genome Biodiversity Network; GMI: Global Microbial Identifier; GSC: Genomics Standards Consortium; iDigBio: Integrated Digitized Biocollections; INSDC: International Nucleotide Sequence Database Collaboration; IPT: Integrated Publishing Toolkit; JATS: Journal Article Tag Suite; MIAME: Minimum Information About a Microarray Experiment; MINSEQE: Minimum Information about a high-throughput nucleotide SEQuencing Experiment; MIxS: Minimum Information about any (x) Sequence; MS: mass spectrometry; OBIS: Ocean Biogeographic Information System; OTU: operational taxonomic units

Competing interests

The authors declare that they have no competing interests.

Funding

This research has received funding from the European Union’s Horizon 2020 research and innovation programme under the Marie Sklodowska-Curie grant agreement IGNITE (No 764840) and from Pensoft Publishers.

References

1. Hey, T. and Trefethen, A., 2003. The Data Deluge: An e-Science Perspective. In: Grid Computing - Making the Global Infrastructure a RealityHey, A J G and Trefethen, A E (2003) . In, Berman, F, Fox, G C and Hey, A J G (eds.) Grid Computing - Making the Global Infrastructure a Reality. Wiley and Sons, pp.809-824.
2. Perez-Riverol, Y., Zorin, A., Dass, G., Vu, M., Xu, P., Glont, M., Vizcaíno, J., Jarnuczak, A., Petryszak, R., Ping, P. and Hermjakob, H., 2019. Quantifying the impact of public omics data. Nature Communications, 10(1).
3. *Darwin Tree Of Life*. 2020. Darwintreeoflife.org. https://www.darwintreeoflife.org/. Accessed on 8 June 2020.
4. *Earth BioGenome Project*. 2020. Earth Biogenome Project. https://www.earthbiogenome.org/. Accessed on 8 June 2020.
5. *Fondation Tara Océan*. 2020. Fondation Tara Océan. https://oceans.taraexpeditions.org/en/. Accessed on 8 June 2020.
6. Schriml, L., Chuvochina, M., Davies, N., Eloe-Fadrosh, E., Finn, R., Hugenholtz, P., Hunter, C., Hurwitz, B., Kyrpides, N., Meyer, F., Mizrachi, I., Sansone, S., Sutton, G., Tighe, S. and Walls, R., 2020. COVID-19 pandemic reveals the peril of ignoring metadata standards. Scientific Data, 7(1). https://doi.org/10.1038/s41597-020-0524-5
7. Karsch-Mizrachi, I., Takagi, T. and Cochrane, G., 2017. The international nucleotide sequence database collaboration. Nucleic Acids Research, 46(D1), pp.D48-D51.
8. Thessen, A. and Patterson, D., 2011. Data issues in the life sciences. ZooKeys, 150, pp.15-51.
9. *GBIF: The Global Biodiversity Information Facility*. 2020. What is GBIF?. https://www.gbif.org/what-is-gbif. Accessed on 25 June 2020.
10. *iDigBio*. 2020. https://www.idigbio.org/. Accessed on 25 June 2020.
11. DiSSCo. 2020. Home - Dissco. https://www.dissco.eu/. Accessed on 25 June 2020.
12. *OBIS (2020) Ocean Biodiversity Information System*. Intergovernmental Oceanographic Commission of UNESCO. www.iobis.org. Accessed on 25 June 2020.
13. Droege, G., Barker, K., Astrin, J., Bartels, P., Butler, C., Cantrill, D., Coddington, J., Forest, F., Gemeinholzer, B., Hobern, D., Mackenzie-Dodds, J., Ó Tuama, É., Petersen, G., Sanjur, O., Schindel, D. and Seberg, O., 2013. The Global Genome Biodiversity Network (GGBN) Data Portal. Nucleic Acids Research, 42(D1), pp.D607-D612.
14. DataONE (2020) *Data Observation Network For Earth | Dataone*. https://www.dataone.org. Accessed on 24 November 2020.
15. GBIF Data standards (2020). Data Standards. https://www.gbif.org/standards. Accessed on 24 November 2020.

16. Wieczorek, J., Bloom, D., Guralnick, R., Blum, S., Döring, M., Giovanni, R., Robertson, T. and Vieglais, D., 2012. Darwin Core: An Evolving Community-Developed Biodiversity Data Standard. PLoS ONE, 7(1), p.e29715. https://doi.org/10.1371/journal.pone.0029715
17. *What Is Darwin Core, And Why Does It Matter?*. 2020. Gbif.org. https://www.gbif.org/darwin-core. Accessed on 15 July 2020.
18. Droege, G., Barker, K., Seberg, O., Coddington, J., Benson, E., Berendsohn, W., Bunk, B., Butler, C., Cawsey, E., Deck, J., Döring, M., Flemons, P., Gemeinholzer, B., Güntsch, A., Hollowell, T., Kelbert, P., Kostadinov, I., Kottmann, R., Lawlor, R., Lyal, C., Mackenzie-Dodds, J., Meyer, C., Mulcahy, D., Nussbeck, S., O'Tuama, É., Orrell, T., Petersen, G., Robertson, T., Söhngen, C., Whitacre, J., Wieczorek, J., Yilmaz, P., Zetzsche, H., Zhang, Y. and Zhou, X., 2016. The Global Genome Biodiversity Network (GGBN) Data Standard specification. Database, 2016, p.baw125.
19. Holetschek, J., Dröge, G., Güntsch, A. and Berendsohn, W., 2012. The ABCD of primary biodiversity data access. Plant Biosystems - An International Journal Dealing with all Aspects of Plant Biology, 146(4), pp.771-779.
20. Field, D., Amaral-Zettler, L., Cochrane, G., Cole, J., Dawyndt, P., Garrity, G., Gilbert, J., Glöckner, F., Hirschman, L., Karsch-Mizrachi, I., Klenk, H., Knight, R., Kottmann, R., Kyrpides, N., Meyer, F., San Gil, I., Sansone, S., Schriml, L., Sterk, P., Tatusova, T., Ussery, D., White, O. and Wooley, J., 2011. The Genomic Standards Consortium. PLoS Biology, 9(6), p.e1001088.

21. *Forside - Global Microbial Identifier*. 2020. Global Microbial Identifier. http://www.globalmicrobialidentifier.org/. Accessed on 15 July 2020.
22. Yilmaz, P., Kottmann, R., Field, D., Knight, R., Cole, J., Amaral-Zettler, L., Gilbert, J., Karsch-Mizrachi, I., Johnston, A., Cochrane, G., Vaughan, R., Hunter, C., Park, J., Morrison, N., Rocca-Serra, P., Sterk, P., Arumugam, M., Bailey, M., Baumgartner, L., Birren, B., Blaser, M., Bonazzi, V., Booth, T., Bork, P., Bushman, F., Buttigieg, P., Chain, P., Charlson, E., Costello, E., Huot-Creasy, H., Dawyndt, P., DeSantis, T., Fierer, N., Fuhrman, J., Gallery, R., Gevers, D., Gibbs, R., Gil, I., Gonzalez, A., Gordon, J., Guralnick, R., Hankeln, W., Highlander, S., Hugenholtz, P., Jansson, J., Kau, A., Kelley, S., Kennedy, J., Knights, D., Koren, O., Kuczynski, J., Kyrpides, N., Larsen, R., Lauber, C., Legg, T., Ley, R., Lozupone, C., Ludwig, W., Lyons, D., Maguire, E., Methé, B., Meyer, F., Muegge, B., Nakielny, S., Nelson, K., Nemergut, D., Neufeld, J., Newbold, L., Oliver, A., Pace, N., Palanisamy, G., Peplies, J., Petrosino, J., Proctor, L., Pruesse, E., Quast, C., Raes, J., Ratnasingham, S., Ravel, J., Relman, D., Assunta-Sansone, S., Schloss, P., Schriml, L., Sinha, R., Smith, M., Sodergren, E., Spor, A., Stombaugh, J., Tiedje, J., Ward, D., Weinstock, G., Wendel, D., White, O., Whiteley, A., Wilke, A., Wortman, J., Yatsunenko, T. and Glöckner, F., 2011. Minimum information about a marker gene sequence (MIMARKS) and minimum information about any (x) sequence (MIxS) specifications. Nature Biotechnology, 29(5), pp.415-420.
23. Athar, A., Füllgrabe, A., George, N., Iqbal, H., Huerta, L., Ali, A., Snow, C., Fonseca, N., Petryszak, R., Papatheodorou, I., Sarkans, U. and Brazma, A., 2018. ArrayExpress update – from bulk to single-cell expression data. *Nucleic Acids Research*, 47(D1), pp.D711-D715.
24. *Biosamples*. 2020. Ebi.ac.uk.https://www.ebi.ac.uk/biosamples/. Accessed on 21 May 2020.
25. *FGED: MINSEQE.* 2020. Fged.org. http://fged.org/projects/minseqe/. Accessed on 25 June 2020.
26. GEOME. 2020. *Genomic Observatories Metadatabase*. https://geome-db.org/. Accessed on 24 November 2020.
27. Deck, J., Gaither, M.R., Ewing, R., Bird, C.E., Davies, N., et al., 2017. The Genomic Observatories Metadatabase (GeOMe): A new repository for field and sampling event metadata associated with genetic samples. PLOS Biology, 15(8): e2002925. https://doi.org/10.1371/journal.pbio.2002925
28. Sansone, SA., Rocca-Serra, P., Field, D. et al, 2012. Toward interoperable bioscience data. *Nat Genet,* 44, 121–126. https://doi.org/10.1038/ng.1054
29. Rocca-Serra, P., Brandizi, M., Maguire, E., Sklyar, N., Taylor, C., Begley, K., Field, D., Harris, S., Hide, W., Hofmann, O., Neumann, S., Sterk, P., Tong, W., & Sansone, S. A., 2010. ISA software suite: supporting standards-compliant experimental annotation and enabling curation at the community level. *Bioinformatics (Oxford, England)*, *26*(18), 2354–2356. https://doi.org/10.1093/bioinformatics/btq415

30. Metabolights. 2020. *Metabolights - Metabolomics Experiments And Derived Information*. Ebi.ac.uk. https://www.ebi.ac.uk/metabolights/. Accessed on 24 November 2020.
31. GigaDB. 2020. GigaDB. GigaScience Press. http://gigadb.org/. Accessed on 24 November 2020.
32. FORCE11. 2016. *The FAIR Data Principles*. https://www.force11.org/group/fairgroup/fairprinciples. Accessed on 12 June 2020.
33. Sansone, S., McQuilton, P., Rocca-Serra, P. et al. FAIRsharing as a community approach to standards, repositories and policies. Nat Biotechnol 37, 358–367 (2019). https://doi.org/10.1038/s41587-019-0080-8
34. Chavan, V. and Penev, L., 2011. The data paper: a mechanism to incentivize data publishing in biodiversity science. BMC Bioinformatics, 12(S15). https://doi.org/10.1186/1471-2105-12-S15-S2
35. *Earth System Science Data*. 2020. http://www.earth-syst-sci-data.net/. Accessed on 7 July 2020.
36. Five years of Scientific Data. Sci Data 6, 72 (2019). https://doi.org/10.1038/s41597-019-0065-y
37. Penev L, Mietchen D, Chavan V, Hagedorn G, Remsen D, Smith V, Shotton D (2011). Pensoft Data Publishing Policies and Guidelines for Biodiversity Data. Pensoft Publishers, http://www.pensoft.net/J_FILES/Pensoft_Data_Publishing_Policies_and_Guidelines.pdf.
38. Penev, L., Mietchen, D., Chavan, V., Hagedorn, G., Smith, V., Shotton, D., Ó Tuama, É., Senderov, V., Georgiev, T., Stoev, P., Groom, Q., Remsen, D. and Edmunds, S., 2017. Strategies and guidelines for scholarly publishing of biodiversity data. Research Ideas and Outcomes, 3, p.e12431.
39. Penev L, Georgiev T, Geshev P, Demirov S, Senderov V, Kuzmova I, Kostadinova I, Peneva S, Stoev P (2017) ARPHA-BioDiv: A toolbox for scholarly publication and dissemination of biodiversity data based on the ARPHA Publishing Platform. Research Ideas and Outcomes 3: e13088. https://doi.org/10.3897/rio.3.e13088
40. Pensoft blog. 2020. *How To Import Data Papers From GBIF, Dataone And LTER Metadata*. https://blog.pensoft.net/2016/05/18/how-to-import-data-papers-from-gbif-data-one-and-lter-metadata/. Accessed on 24 November 2020.
41. Filter M, Candela L, Guillier L, Nauta M, Georgiev T, Stoev P, Penev L., 2019. Open Science meets Food Modelling: Introducing the Food Modelling Journal (FMJ). Food Modelling Journal 1: e46561. https://doi.org/10.3897/fmj.1.46561

42. *The ENA Metadata Model*. 2020. Ena-docs.readthedocs.io. https://ena-docs.readthedocs.io/en/latest/submit/general-guide/metadata.htm. Accessed on 5 May 2020.
43. Carpenter, E., Matasci, N., Ayyampalayam, S., Wu, S., Sun, J., Yu, J., Jimenez Vieira, F., Bowler, C., Dorrell, R., Gitzendanner, M., Li, L., Du, W., K. Ullrich, K., Wickett, N., Barkmann, T., Barker, M., Leebens-Mack, J. and Wong, G., 2019. Access to RNA-sequencing data from 1,173 plant species: The 1000 Plant transcriptomes initiative (1KP). GigaScience, 8(10).
44. Filho, J., Jorge, S., Kremer, F., de Oliveira, N., Campos, V., da Silva Pinto, L., Dellagostin, O., Feijó, R., de Menezes, F., de Sousa, O., Maggioni, R. and Marins, L., 2018. Complete genome sequence of native Bacillus cereus strains isolated from intestinal tract of the crab Ucides sp. Data in Brief, 16, pp.381-385.
45. Zhou, Y., Xiao, S., Lin, G., Chen, D., Cen, W., Xue, T., Liu, Z., Zhong, J., Chen, Y., Xiao, Y., Chen, J., Guo, Y., Chen, Y., Zhang, Y., Hu, X. and Huang, Z., 2019. Chromosome genome assembly and annotation of the yellowbelly pufferfish with PacBio and Hi-C sequencing data. Scientific Data, 6(1).
46. Programmatic Access To ENA Data. 2020. Ebi.ac.uk. https://www.ebi.ac.uk/ena/browse/programmatic-access. Accessed on 21 May 2020.
47. Winston Chang, Joe Cheng, JJ Allaire, Yihui Xie and Jonathan McPherson (2020). shiny: Web Application Framework for R. R package version 1.5.0. http://shiny.rstudio.com
48. RStudio, PBC, 2020. Shinyapps.io. Boston, Massachusetts, USA: RStudio, PBC.
49. R Core Team (2020). R: A language and environment for statistical computing. R Foundation for Statistical Computing, Vienna, Austria. URL https://www.R-project.org/.
50. National Information Standards Organization, 2019. ANSI/NISO Z39.96-2019, JATS: Journal Article Tag Suite, Version 1.2 | NISO Website. Niso.org. https://www.niso.org/publications/z3996-2019-jats. Accessed on 10 August 2020.
51. Colin Fay, Vincent Guyader, Sébastien Rochette and Cervan Girard (2020). golem: A Framework for Robust Shiny Applications. R package version 0.3.0. https://github.com/ThinkR-open/golem
52. Dimitrova, Mariya, 2020. omicsdatapaper: OMICS data paper R shiny app as golem. GitHub. https://github.com/pensoft/omicsdatapaper. Accessed on 24 November 2020.
53. Dimitrova, Mariya, 2020. *Omics Data Paper Generator*. https://mdmtrv.shinyapps.io/Omics_data_paper/. Accessed on 18 August 2020.

54. Dimitrova, Mariya, 2020. Pensoft/Omics-Data-Paper-Shinyapp. GitHub. https://github.com/pensoft/omics-data-paper-shinyapp. Accessed on 18 August 2020.
55. RStudio, PBC, 2020. Rstudio.Cloud. Boston, Massachusetts, USA: RStudio, PBC.
56. Penev, L., Dimitrova, M., Kostadinova, I., Georgiev, T., Agosti, D. and Poelen, J., 2020. *How To Get Data From Research Articles Back Into The Research Cycle At No Additional Costs?*. Pensoft blog. https://blog.pensoft.net/2020/04/24/how-to-get-data-from-research-articles-back-into-the-research-cycle-%D0%B0t-no-additional-costs/. Accessed on 24 November 2020.
57. Biodiversity Data Journal. 2020. Data Publishing Guidelines: Linked data table for primary biodiversity data.https://bdj.pensoft.net/about#Linkeddatatableforprimarybiodiversitydata. Accessed on 24 November 2020.
58. *Arrayexpress - Data Access Policy*. 2020. Ebi.ac.uk. https://www.ebi.ac.uk/arrayexpress/help/data_availability.html. Accessed on 5 May 2020.

59. JATS4R, 2020. *JATS4R Validator*. Validator.jats4r.org. https://validator.jats4r.org/. Accessed on 18 August 2020.
60. *ESA's Ecological Archives*. 2020. Esapubs.org. http://www.esapubs.org/archive/default.htm. Accessed on 5 May 2020.
61. Smith, M., 2011. Data Papers in the Network Era. In: *Charleston Library Conference*. Against the Grain Press, LLC. http://dx.doi.org/10.5703/1288284314871. Accessed on 21 May 2020.
62. Robertson, T., Döring, M., Guralnick, R., Bloom, D., Wieczorek, J., Braak, K., Otegui, J., Russell, L. and Desmet, P., 2014. The GBIF Integrated Publishing Toolkit: Facilitating the Efficient Publishing of Biodiversity Data on the Internet. *PLoS ONE*, 9(8), p.e102623.
63. *Data Papers*. 2020. Gbif.org. https://www.gbif.org/data-papers. Accessed on 5 May 2020.
64. Joachim Schöpfel, Dominic Farace, Hélène Prost, Antonella Zane. Data papers as a new form of knowledge organization in the field of research data. 12ème Colloque international d'ISKO-France :Données et mégadonnées ouvertes en SHS : de nouveaux enjeux pour l'état et l'organisation des connaissances ?, ISKO France, Oct 2019, Montpellier, France. halshs-02284548
65. Smith, V., Georgiev, T., Stoev, P., Biserkov, J., Miller, J., Livermore, L., Baker, E., Mietchen, D., Couvreur, T., Mueller, G., Dikow, T., Helgen, K., Frank, J., Agosti, D., Roberts, D. and Penev, L., 2013. Beyond dead trees: integrating the scientific process in the Biodiversity Data Journal. *Biodiversity Data Journal*, 1, p.e995. https://doi.org/10.3897/BDJ.1.e995
66. Senderov, V., Georgiev, T. and Penev, L., 2016. Online direct import of specimen records into manuscripts and automatic creation of data papers from biological databases. *Research Ideas and Outcomes*, 2, p.e10617
67. Elie Arnaud, 2020. MetaShARK-v2. GitHub. https://github.com/earnaud/MetaShARK-v2. Accessed on 19 August 2020.
68. Susanna-Assunta Sansone, Philippe Rocca-Serra, Massimiliano Izzo, 2020. Datascriptor. https://datascriptor.org/. Accessed on 19 August 2020.
69. Martens, L. and Vizcaíno, J., 2017. A Golden Age for Working with Public Proteomics Data. *Trends in Biochemical Sciences*, 42(5), pp.333-341.
70. Pensoft Publishers, 2020. *Data Quality Checklist And Recommendations*. Bdj.pensoft.net. https://bdj.pensoft.net/about#DataQualityChecklistandRecommendations. Accessed on 18 August 2020.
71. Kolesnikov N. et al., 2015. *ArrayExpress update-simplifying data submissions*. Nucleic Acids Res, doi:10.1093/nar/gku1057 . Pubmed ID 25361974.
72. Arrayexpress/Annotare2. GitHub. 2012. Web. Available at: https://github.com/arrayexpress/annotare2
73. Xue, H., Li, J., Xie, H. and Wang, Y., 2018. Review of Drug Repositioning Approaches and Resources. *International Journal of Biological Sciences*, 14(10), pp.1232-1244.

74. McDonald, D., Clemente, J., Kuczynski, J., Rideout, J., Stombaugh, J., Wendel, D., Wilke, A., Huse, S., Hufnagle, J., Meyer, F., Knight, R. and Caporaso, J., 2012. The Biological Observation Matrix (BIOM) format or: how I learned to stop worrying and love the ome-ome. *GigaScience*, 1(1).

Figure 1

Click here to access/download;Figure;figure_1.pdf

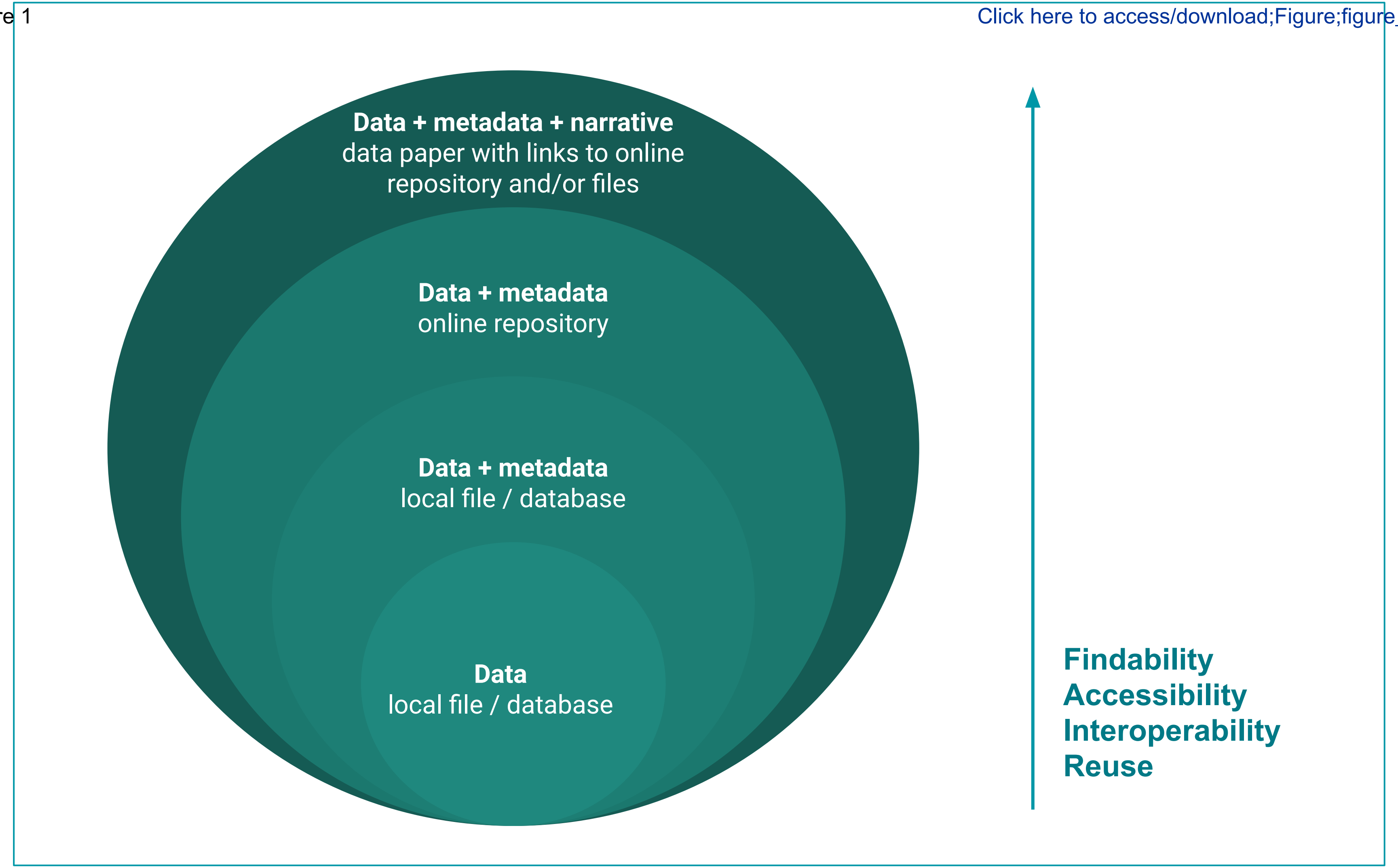

Figure 2

Click here to access/download;Figure;figure_2.pdf

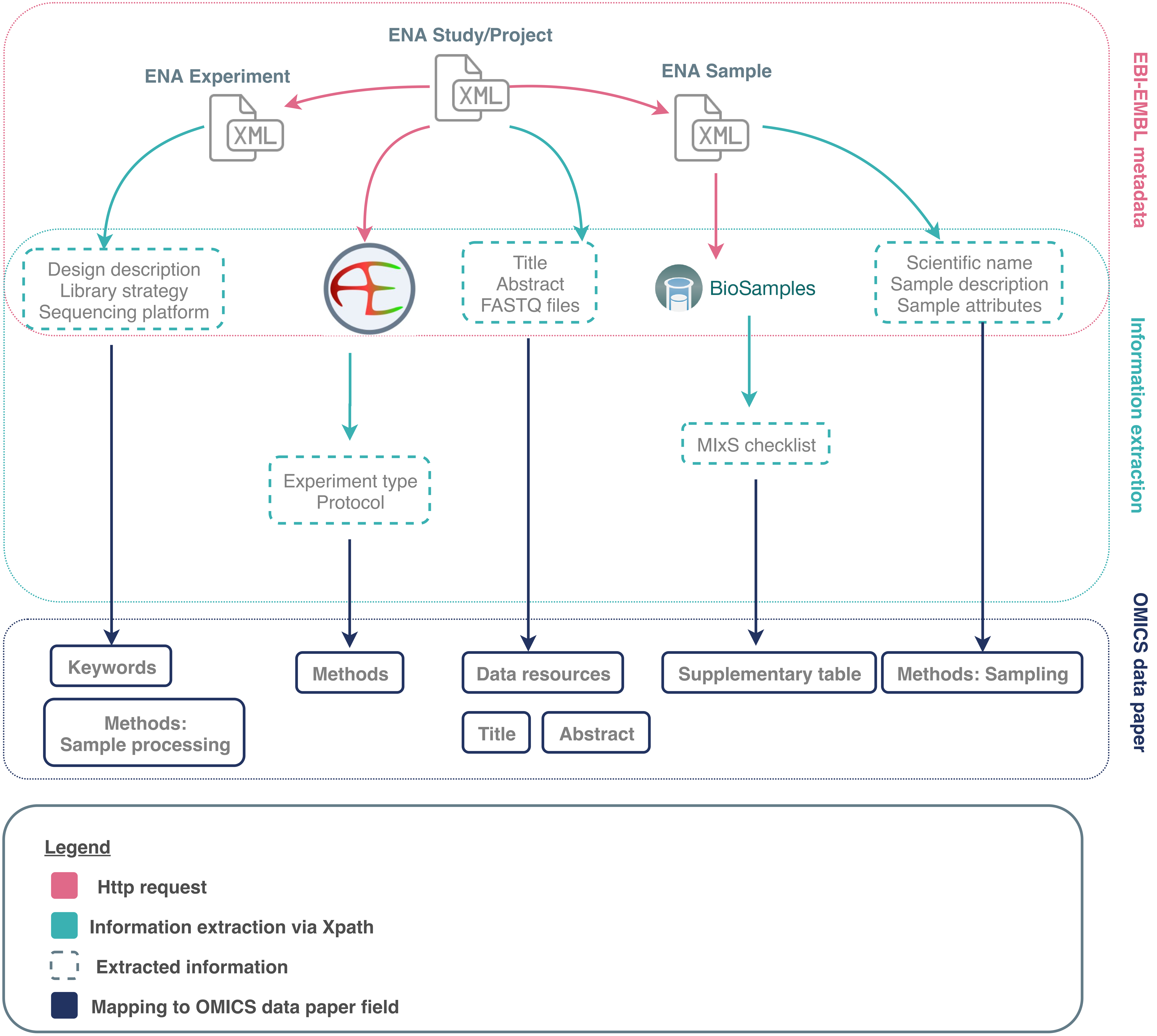

Figure 3

Click here to access/download;Figure;figure_3.pdf

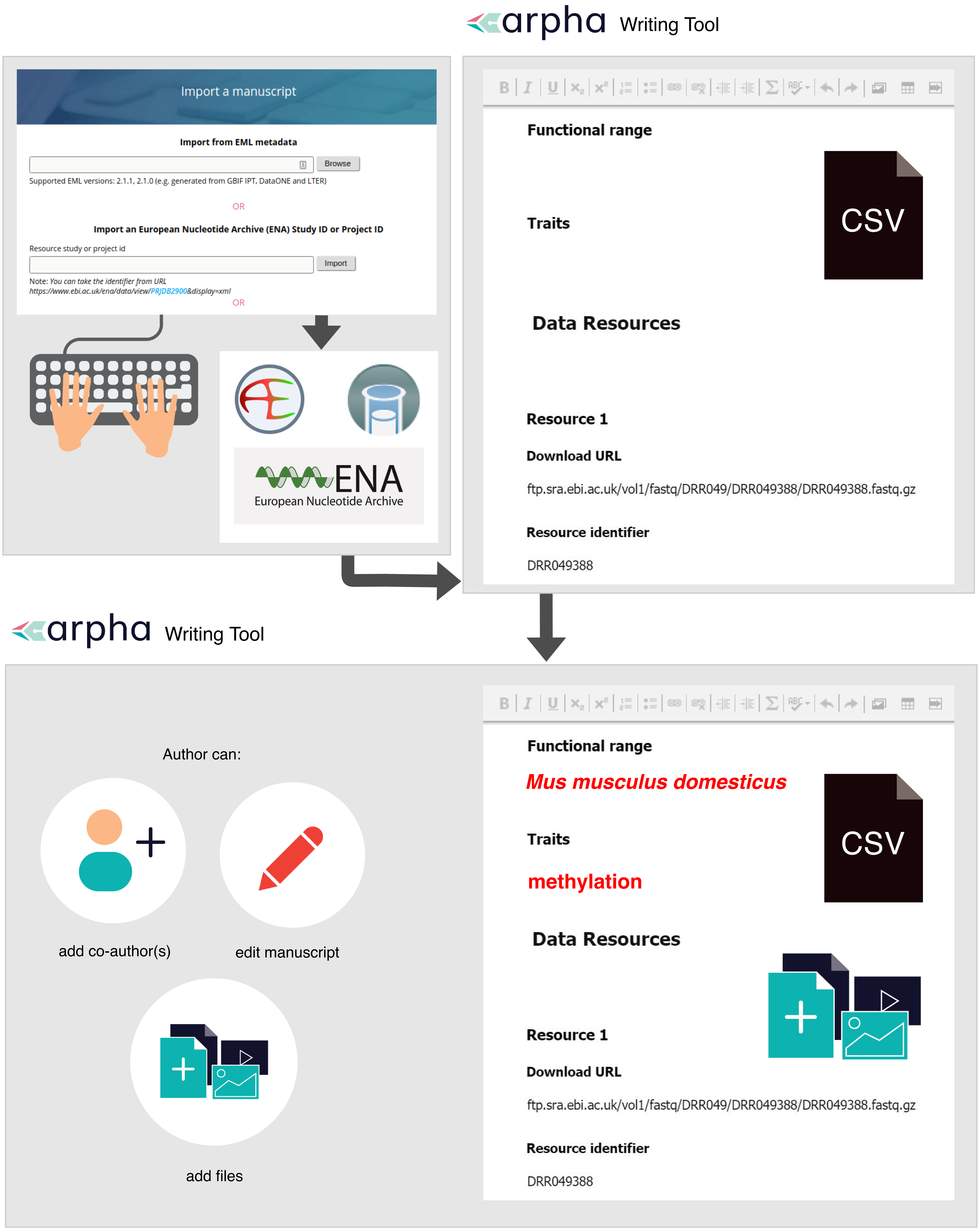

Cover Letter Click here to access/download;Personal Cover;cover_letter_and_reviewer_comments.docx

Mariya Dimitrova
Corresponding author

Cover Letter

Resubmission of manuscript "*A streamlined workflow for conversion, peer review and publication of genomics metadata as Omics Data Papers*"

Dr. Scott Edmunds,
Editor-in-Chief
GigaScience Journal

November 29th, 2020

Dear Dr. Edmunds,

Thank you very much for the detailed and timely reviews of our manuscript "*A streamlined workflow for conversion, peer review and publication of genomics metadata as Omics Data Papers"* and for the encouragement to pursue a resubmission!

We fully understand and appreciate the critics and respect the scrutinous reproducibility criteria of Giga Science. Therefore, we have made changes to the code which now enable installation of the RShiny app as a package, as recommended by the editor and one of the reviewers, thus hopefully responding to the rigorous reproducibility criteria of GigaScience. The package is openly available on Github: https://github.com/pensoft/omicsdatapaper, including installation and execution instructions and technical requirements. The information on that is updated in the Availability of supporting source code and requirements section of the revised manuscript. The interactive web app is unchanged.

Also, we have made changes throughout the manuscript to reflect that: 1) it describes a prototype workflow for genomic data which could be easily extended to omics but does not claim to be exhaustive 2) the R Shiny app is not production grade (only meant for demonstration purposes) but the ARPHA workflow is, 3) several other changes to address reviewers' and editor's comments

A more detailed account of our feedback to the comments and the respective changes in the revised manuscript is enclosed in the document below which consists of:

Response to Reviewer 1 (Yvan Le Bras) Reject

Response to Reviewer 2 (Philippe Rocca-Serra) Major revision

Response to Reviewer 3 (Christine Ferguson) Minor revision

Best regards,

Mariya Dimitrova
Lead author on behalf of all co-authors

Decision letter

Dear Ms Dimitrova,

Thank you for considering GigaScience. Apologies it has taken some time to review this but we now have sufficient advice and testing. Peer review of your manuscript is therefore now complete and, in the light of the reports, and my own assessment as Editor, I regret to inform you that your manuscript cannot be accepted for publication in GigaScience.

Please find the reviewers' reports at the end of this email. Please also take a moment to check our website at https://www.editorialmanager.com/giga/ for any additional comments that were saved as attachments.

Unfortunately there were a lot of comments, and while we need to take into account some of the reviewers were working in potentially very close areas, GigaScience has very strict policies regarding reproducibility and reusability ("installability", and potential for reuse and uptake) that their comments aligned with. Many other journals do not require this level of detail for software, but if you were still interested in publishing it in Gigascience and were able to carry out all this additional work then we could potentially consider a resubmission later. Issues like the documentation, installation scripts, better justification of what is added (particularly to convince a non-publishing audience), and issues covering the future (e.g. plans for updatability).

If at some stage you are able to fully address these concerns, you may wish to submit to GigaScience with the revised manuscript. If you are able to do this a full cover letter, explaining the revisions made, should accompany the submission.

I hope the feedback is useful and I wish you every success with your research and hope that you will consider us again in the future.

Best wishes,

Scott Edmunds, Ph.D.
GigaScience
www.gigasciencejournal.com

Response to Reviewer 1 (Yvan Le Bras) Reject

Dear Dr. Le Bras,

Thank you for the extensive review and useful comments! Your thorough comments and suggestions guided us to improve our manuscript and the R shiny app and to resubmit. We also want to thank you for your positive words on the importance of developing this kind of workflows and the approach we have used.

Please find enclosed inline below our feedback and account of the respective changes in the manuscript.

Respectfully,
Mariya Dimitrova

Review and feedback:

Competing interests:

I think I can have competing interests as I am working on a similar development and submit a project with similar development for funding. This is one reason why I prefer not giving any recommandation.

Comments to author:

This is a hot topic for me and as I am working on a very similar development, I prefer to say I have potentiel competing interest and not give any recommandation to editors. That being said, I think of interest to still review your article and provide my comments. I must also apologize for my approximate english and maybe the manner I wrote my review, quite rapidly even if I take times to read the article and test the related source code.

I really want to thank authors for this very interesting article, I think there is a lot of points who are very important, as trying creating a manner to incite researchers to create more rich metadata associated to their data. I also really think the overall proposed approach can be a good one, but I have serious doubts regarding the proposed operationalization, notably due to the poor information actually found in ENA metadata.

> **Feedback**: *The aim of the data paper workflow is exactly to correct the deficiencies of metadata at ENA (or other repositories) by*

enhancing the metadata descriptions in the data paper and not just to import and publish ENA metadata as they are. The link between the permanent identifiers of the data (ProjectID) and the data paper (DOI) ensures the dataset is far better described with extended and peer reviewed metadata and is potentially far more re-usable to others.

I finally choose the "reject" recomandation as it seems to me that 1/ the described workflow is not related to omics, but "just" genomics,

Feedback: *We agree that the workflow was elaborated and tested for genomic data, although analogous workflows for other kinds of omics metadata are relatively easy to implement.*

Changes in the manuscript: *We have amended the manuscript to reflect that the workflow is prototyped for genomic metadata, whereas the conceptualisation and template could be applied to omics data in a future work.*

2/ I think the described automated workflow can't really help to create "high-quality metadata records" or "encourages authors to improve the quality of their metadata to achieve a truly FAIR data wolrd".

Feedback*: The workflow is not just a plain conversion of the existing metadata but gives the authors the possibility to amend, edit and review the data paper manuscript once it is imported in the ARPHa Writing Tool (AWT) and further in the Biodiversity Data Journal (BDJ). Moreover, the authors can also create a data paper manually in AWT, by filling in the relevant sections of the data paper template where the link to the described data should be inserted as well.*

We agree that "high-quality" is an ambiguous term and have changed the manuscript to explain that we mean "more extensive, or extended" metadata. As previously stated, the omics data paper enables more thorough descriptions of metadata and also improves the visibility and findability of datasets. This is achieved through links between the dataset and the publication, which is indexed by various research platforms and even social media. This raises the visibility and findability, thus the probability to comment on or reuse

the dataset and related omics project to another level. The peer-review and data auditing processes bestow more credibility to the dataset, which is what we meant by "high-quality". Data auditing before publication is complementary to data curation performed by the data repositories and can help to uncover missing essential metadata records which ENA or another repository have let pass through their "curation filter". Finally, the described workflow does not claim to automatically improve metadata but rather to facilitate authors to describe their datasets more thoroughly with the help of the provided omics data paper template.

Changes in the manuscript: *We have made changes in several parts of the manuscript reflecting the above comment. We indicated that the workflow and omics data publishing in general aim to complement the existing practice of data deposition to recognised repositories, as demonstrated by the newly added Figure 1.*

Article

I really want to thank authors for this very interesting article, I think there is a lot of points who are very important, as trying creating a manner to incite researchers to create more rich metadata associated to their data. I also really think the overall proposed approach can be a good one, but I have serious doubts regarding the proposed operationalization, notably due to the poor information actually found in ENA metadata.

Feedback: *We agree with the reviewer's statement about the "poor information actually found in ENA metadata". Please see the first paragraph in the Feedback above where we justify our approach to improve the metadata quality through data papers.*

General comment

- "Omics data paper" seems to me not an appropriate manner to call it as it focus on genomics / genomics related data, and standards. In my opinion, such an omics data paper will propose a way to integrate at least genomics, proteomics and metabolomics data.

  Feedback: *We agree and have changed the term for the automated workflow described in the manuscript to "genomic". However, the concept and template are still applicable to all omics metadata. Authors can write manuscripts without the help of the conversion*

workflow in the ARPHA Writing Tool if they want to describe a non-genomic dataset.

- Did you look at ISAcommons and related ISAtools ? It seems to me this initiative and tools of interest to consider reaching goals you mention to create “”””real”””” (at least genomics/proteomics/metabolomics) omics data paper with rich metadata considering notably sampling methods or data processing.

  Feedback: *We are aware about the ISAcommons and ISAtools and recognise that they have similar aims to our project.*

  Changes in the manuscript: *We have included a discussion on ISAcommons and ISAtools in the* *3rd paragraph of the Introduction* *of the revised manuscript.*

- To incite data producers to better structure their data through rich metadata addition; I don’t think acting at the level of such genomics standard will be very efficient. Maybe it can be more useful to propose this at a “higher” (more federative) level as ISAcommons related tools and standards or others standards like EML, as they are already used by scientists adding relatively rich metadata.

  Feedback: *Use of community accepted standards, ISA included, and omics data paper publishing are complimentary. We do not aim to replace this important standard with another one, but we rather aim to create a publishing mechanism which stimulates authors to create extended metadata descriptors. In addition, Pensoft has already developed a workflow for import of EML metadata into manuscripts and successfully uses it in its routine publishing practice since 2010. The current project aims to replicate that workflow but for genomic data and more widely for other omics data in future.*

Abstract

- Looking at the abstract, it seems to me relatively a lack of relation between the Background and the Findings.

  Changes in the manuscript: *We have revised all sections of the abstract to address the comment; those changes have been synchronised with the changes made to the rest of the manuscript.*

Introduction

- On “major infrastructures to handle higher-order biodiversity data” listed, DataOne is not cited, it seems to me this is one, with more than one millions data files for 77Tb

  Changes in the manuscript: *We agree with this comment and have cited DataONE as one of such major infrastructures in the second paragraph of the Introduction. Actually, since several years, Pensoft has integrated its EML conversion workflow to create “biodiversity”and “ecological” data papers with DataOne, to complement the already existing integration with GBIF and LTER.*

- Authors mention “community-accepted metadata standards” and in relation “GBIF uses the Darwin Core Standard” who is more a data standard than a metadata standard, used to create Darwin Core Archive containing data files where Darwin core terms are used to fill the headers, when a limited version of EML, the EML GBIF profile, is used as a metadata standard

  Changes in the manuscript: *We agree and fully understand that DwC is a data standard while EML is a metadata standard and have corrected it in the text (second paragraph of the Introduction).*

Methods

- Authors mention the identification of “high-level needs of the omics communities”. This means common fields, or minimum shared information?

  Feedback*: We mean the best practice to publish high-quality metadata and not a standard-based solution. Actually, Pensoft has implemented conversion and import workflows for both EML to create data paper manuscripts (see Chavan and Penev 2011,*https://doi.org/10.1186/1471-2105-12-S15-S2*) and Darwin Core to import primary biodiversity data into manuscripts (see Smith et al 2013,*https://doi.org/10.3897/BDJ.1.e995)

  Changes in the manuscript*: We have made changes in the Approach subsection of the Methods section to clarify the difference between EML and Darwin Core.*

- Testing the “RStudio cloud” based Shiny app, it seems to me that the example ENA “PRJDB2900” is maybe not a good one as it is really poor in terms of metadata, but maybe this point a major issue as metadata is often very poor on such kind of repository, and linked to this kind of standard. Notably a lack in “sampling method”, “”only”” “sample_name”, “strain”, “dev_stage”, “genotype” and ”host_body_site”, and nothing in “Environmental profile”, “Geographic range”, nothing in “Data Processing”.

  Feedback*: You are correct that this points to a major issue but there is also the fact that not all sections of the omics data paper template are meant to be automatically filled in by the workflow. Many of them are meant to be filled in manually and the authors have the rights and the option to include this missing information in the relevant sections of the data paper manuscript.*

  Changes in the manuscript*: We have made changes to the manuscript to reflect that (in first paragraph of section* “Workflow for extracting relevant metadata from ENA XML files” and the second paragraph of “Integration of metadata extraction workflow with the ARPHA Writing Tool” section)

- Related to previous comment, authors mention the fact that “certain sections” “were made mandatory” but if content is as poor as the content seen on the example accession, it seems to me that this is not very useful.

  Feedback: *We agree that it is not entirely clear what is meant by “made mandatory”.*

  Changes in the manuscript*: We have clarified that under “mandatory” we meant “mandatory in ARPHA” for the manuscript to proceed to review. We have clarified that in the second paragraph of the “Integration of metadata extraction workflow with the ARPHA Writing Tool” section.*

- Authors propose creation of a “long format table” from retrieved BioSamples metadata, I suppose because a CSV file is more comfortable for users without programming knowledge, but it seems to me this format with “blocks of lines” by BioSample who

can have different number of lines from one block to another is not really easy to deal with and maybe can be at the origin of error.

Feedback: *Long format tables in such universally used format as CSV can easily be transformed to wide format tables in many of the data processing softwares.*

Findings

- Totally agree with your sentence "The described dataset is at the core of the data paper, but the methodology required to obtain it is just as valuable as the data itself.
- Regarding Table 1, some questions come to my mind:
- How many records on ENA have information related to "environmental and geographic characteristics of the locations where their material was collected"?

  Feedback: *We have no information regarding the completeness of ENA metadata records across the different projects. It may well be that most of the datasets lack this kind of information. On the other side, this is exactly what the workflow offers, that is the authors are encouraged to supplement imported metadata with additional information, such as the abovementioned environmental and geographic characteristics during the process of completion of their manuscripts already in the ARPHA Writing Tool environment.*

- Same question for "Technologies used" / " Sample processing" and "Data Processing".

  Feedback: *Some ENA records do contain information about sequencing technologies which could go in the "Technologies used" section of the "Sample processing" section. For the rest you are correct that there are no ENA records but authors can fill those sections manually in the ARPHA Writing Tool, thus improving the description of their datasets deposited in ENA.*

- Linked to previous questions, is there a manner to update existing ENA records to add metadata information?

  Feedback*: The way metadata is updated at ENA is ENA's own policy, what we do is to enhance records in the data paper completed in the AWT. In a starting EU-funded project, coordinated*

by Pensoft, one of the tasks of the EMBL EBI as a partner is to provide means for easy feedback, annotation and improvement of ENA's metadata records.

- Regarding "Biodiversity profile", "written by the authors" seems to be too bad regarding others Metadata standards and/or terms who can help filling that, notably EML. Moreover, controlled vocabulary can be used to help enrich metadata content, notably Gene Ontology. It appears to me of interest to consider proposing the users using terminological resources notably for taxonomic information.

  ***Feedback**: We agree that it is recommended to use existing standards and vocabularies, so we have added a short paragraph outlining this recommendation in the end of the section "Structure of the OMICS data paper". Authors can attach a supplementary EML file or create their own Linked data table[1], which could serve a similar purpose and still use ontologies to express biodiversity data.*

  The "biodiversity profile" has indeed to be complemented by the authors, however it follows the EML metadata profile used by GBIF and also by Pensoft to create "biodiversity data paper". The process is described in due detail in Chavan and Penev (2011, https://doi.org/10.1186/1471-2105-12-S15-S2) and Penev et al (2017, https://doi.org/10.3897/rio.3.e12431).

- Data resources //XREF_LINK// seems to me not dedicated to inform about a data resource, but more the use of a generic xref term. Isn't it?

  ***Changes in the manuscript**: We have specified that we refer to a //XREF_LINK which has attribute ENA-FASTQ-FILES so it is not any generic xref term but the one linking to the data resource.*

- Concerning "Data statistics", again it would be better to avoid asking the user filling it by hand, is there any manner to propose a way to populate this section from existing metadata fields or other machine actionable resources?

---

[1] *https://blog.pensoft.net/2020/04/24/how-to-get-data-from-research-articles-back-into-the-research-cycle-%D0%B0t-no-additional-costs/*

Feedback: *We give authors the freedom to represent their data statistics in the way they see most valuable and informative to the readers. During the peer-review process they can obtain feedback about the data statistics they have decided to include.*

- Concerning Usage rights, authors mention the fact that open access is default value, can you precise which license? To facilitate reuse of related information and OA initiatives, authors can also choose to only accept OA license, but here it seems this is not the case.

  Feedback: *We have inserted in the manuscript a link to Pensoft's policy regarding data licenses and usage rights outlined in https://riojournal.com/article/12431/instance/3306534/. For data, Pensoft recommends the following data publishing licenses:*

  - *Open Data Commons Attribution License http://www.opendatacommons.org/licenses/by/1.0/*
  - *Creative Commons CC-Zero Waiver http://creativecommons.org/publicdomain/zero/1.0/*
  - *Open Data Commons Public Domain Dedication and License http://www.opendatacommons.org/licenses/pddl/1-0/*

  however it is the decision of the authors and a matter of the data repositories which license to use. The data paper itself is published under the Creative Commons Attribution LIcense (CC-BY 4.0), https://creativecommons.org/licenses/by/4.0/

- Authors mention that "some data paper sections do not have ENA metadata fields associated with them", is some exact? Maybe many can be more appropriate isn't it?

  Feedback: *We agree and have corrected it in the manuscript.*

- Authors mention "and the authors are encouraged to fill in their contents". Are authors encouraged to fill in their contents in the original ENA record? Or on related data paper fields ? And/or is there any feedback planned between data papers and ENA so ENA records can be updated from a data paper creation process?

***Feedback**: Authors are encouraged to provide complete metadata records during the submission process to ENA or afterwards if they want to correct them. They can also create extended metadata descriptions in the omics data paper.*

***Changes in the manuscript:** We have clarified this in the first paragraph of the Findings section. We are in contact with ENA and have proposed to them to link to the data papers describing their datasets.*

- Understanding Figure 1 was not so easy for me. The image quality of the Figure 1 is weak. ArrayExpress is not displayed explicitly on the Figure 1. Maybe the arrow between "ENA Sample" and "BioSamples" has to be dotted as the link is not always there?

  ***Changes in the manuscript**: We have improved the quality of the figure, which is now Figure 2 because we added a different first figure that demonstrates how data papers complement existing methods for metadata sharing.*

- Looking at the statement "thus enhancing manuscripts with high quality metadata", can the authors explain what is "high quality metadata"? Did the authors use metrics to evaluate that, for example metrics related to metadata completeness or others? It seems to me high quality metadata can be related to rich and detailed metadata and semantically connected to meaningful resources for example, and I am not sure the presented system is in line with this.

  ***Feedback**: We agree that 'high-quality' is not well defined term and accept your interpretation that "high-quality" metadata means actually "rich and detailed metadata, semantically connected to meaningful resources".*

  ***Changes in the manuscript**: We have changed the use of "high-quality" to "extended" or 'enhanced' metadata throughout the manuscript.*

- Authors "promote the reuse and interoperability of MIxS compliant metadata sourced from BioSamples", is this sufficient, notably to publish a quality data paper?

***Feedback**: It may not be sufficient but it is certainly beneficial to promote them to the community.*

- I particularly appreciate the JATS transformation functionality

Discussion

- Authors mention "the average number of published data papers continues to grow (Fig. 3)", but looking at the Figure 3, it seems to me that there is like a plateau since 2015 no? It will be interesting to look at such a graph with this number normalized regarding data sharing effort.

  ***Changes in the manuscript**: We agree with the comment and have corrected it in the text to highlight the data paper publishing uptake. We have also removed Figures 3 and 4, which we found to be a bit superfluous in the Discussion section.*

- In the "Comparison with other tools and workflows" sub section, authors mention EML in the first paragraph. Just to be sure the reader can't think this paragraph is related to the EML metadata standard, but to the limited version used by GBIF, it seems to me of interest to specify "EML GBIF profile".

  ***Changes in the manuscript**: Thank you for pointing it out, we have corrected it in the manuscript.*

- In the "Data papers for the field of omics: rationale and benefits" sub section first paragraph, this argue to me to look at others initiatives / standards than thus proposed by ENA, notably to go beyond the data, to publish tools, protocols and others Research products in data paper.
- Regarding "Data papers for the field of omics: rationale and benefits" first point, authors mention "the introduction of data scientists into the publishing process ensures that submitted data and metadata are FAIR". It seems to me, unfortunately, that the use of "ensure" is maybe not useable or this ensures a minimal level of FAIRness maybe.

  ***Changes in the manuscript**: we agree that the word 'ensures' is too strong but we definitely encourage and support authors with our*

workflow to FAIRizise their data to the maximum possible extent. We have corrected the manuscript to clarify that it "ensures to an extent".

- Double checking seems to be a big effort here for a limited impact no?

  ***Feedback**: The data auditing effort (QA/QC) is substantial indeed but proved to be valuable and very useful in improving the data and metadata quality and reuse.*

- Regarding "Data papers for the field of omics: rationale and benefits" second point, authors mention "we came across many datasets with missing or incorrectly formatted metadata fields". Considering the manner researchers are filling ENA metadata, this is really not a surprise for me. That's a reason why I think that maybe focusing on ENA metadata is not a so good idea unless the system allows to help filling retroactively ENA metadata fields.

  ***Feedback:** The retroactive filling of ENA metadata depends on ENA policies, what we do is just to improve the metadata in a third party environment through writing, peer review and publication, linked back to the original ENa metadata record.*

- "Annotaire tool" written instead of "Annotare tool" if I am not wrong.

  ***Changes in the manuscript:** We have corrected it in the text.*

- Regarding "Limitations and future outlooks" section, it seems to me that the described automated workflow works only with ENA metadata records is a huge limitation.

  ***Changes in the manuscript:** We agree and revised the manuscript to state that the automated conversion workflow is a working prototype based on ENA's metadata profiles.*

- Regarding "Limitations and future outlooks" section, I am not sure that motivating the choice of ENA because their metadata structure was easiest to work with is a good point.

Feedback: *We agree. Our motivation was that the links between ENA, BioSamples and ArrayExpress are more clear or in other words easy to "translate" into XPATH and HTTP requests. We think that it is fair to look for the least-complicatedly structured source of metadata when building a prototype workflow. We apply this "minimal viable product" concept because to us it is important to receive feedback and monitor the uptake of the workflow and then make changes to the future versions.*

- Regarding "Limitations and future outlooks" section and the "two-way link" statement, again it seems to me this argue towards the use of another kind of metadata standard, domain-oriented for example and/or another kind of data repository that can refer to ENA files.
-

  Feedback: Well, this is what we have at disposal. This metadata standard may not be ideal but this is what scientists and data repositories use.

OMICS data papers Shiny apps

<u>Interesting points</u>

- Easy and quick to use, with a nice display (choice of color palettes, simple UI)
- Ability to download the files in local with format choice
- A commented and legible code !
- Access to reference and general context from the app

<u>Enhancements</u>

- Turn it into a package of it (cf. {golem})
- Add an installation script (get all required packages). See this link for auto-install packages: https://stackoverflow.com/questions/4090169/elegant-way-to-check-for-missing-packages-and-install-them
- Test with the provided example:
    - use {shinycssloaders} while loading paper
    - avoid empty fields: fill them with a shiny::helpText("No content found") OR do not display them.
    - maybe a way to get to an index of references?
- Code itself:
    - split tasks in subroutines: this allows to use *apply() instead of for() which is more legible and somehow faster and makes bug detection better, rather than "Error on line 579 of app.R"

    - package namespacing (pkg::function) not always applied (e.g. app.R#234 vs app.R#236)
    - use "document outline" if you use RStudio: allows to browse the code quickly and efficiently
- Add a way to browse the downloaded document
    - check for {shinyTree}. A suggestion could be:
    - make a hierarchy of displayed <div> elements (for instance, in Newick format for "Value of the dataset" and its subitems: {Value of the dataset, {Scientific value, Societal value}})
    - when user click on a level of the tree, redirect him to the <div> with attribute 'id' matching the clicked level
- Add a Help to explain:
    - how the downloaded document will be structured
    - some definitions and basic ENA concepts (same example: what are the "scientific value" and "societal value")

Changes in the manuscript*: Thank you for the useful suggestions! We have created a golem package: https://github.com/pensoft/omicsdatapaper. It allows running the R shiny app after local installation of the package. Instructions for installation and running are available on github: https://github.com/pensoft/omicsdatapaper/blob/main/README.md.*
The package imports all required packages and we've fixed package namespacing. We've revised the code and have replaced the use of for() loops with lapply() where appropriate. We have also added additional documentation / help about the omics data paper structure. It is accessible by clicking the "Read more" button in "Omics data paper structure". The previous R shiny app code is still available on GitHub and we've added a script to it to load all required packages.

Response to Reviewer 2 (Philippe Rocca-Serra) Major revision

Dear Dr. Rocca-Serra,

Thank you for taking the time to read and get an in-depth understanding of our manuscript! Your comments and suggestions encouraged us to resubmit the manuscript after making the necessary corrections. We have specified that the workflow is a prototype, hence it currently works with genomic metadata only. We indicate that the workflow and omics data publishing in general do not aim to replace the existing practice of data deposition to recognised repositories, but aim to complement it. We have made changes to the manuscript reflecting all comments we received and have created an installable version of the R shiny app as a golem package, as suggested be the first reviewer. We believe that these additions contribute to the reproducibility of your submission. You can find our replies to your comments below.

Kind regards,
Mariya Dimitrova
Lead author on behalf of all authors

Review and feedback:

Competing interests:
Lead on the Datascriptor project mentioned in the manuscripts

Comments to author:
I thank the authors for a well written and well presented manuscript.

main comments:
1. slight over-claiming when it comes to 'omics'
What the authors have documented as "Omics Data Papers" would benefit from being highlighted as 'sequencing based omics data sets'.
since the targeted repositories are restricted to sequencing based omics and the workflows essentially interrogates insdc resources, thus covering Transcriptomics, Genomics, metagenomics, but leaving out proteomics and metabolomics.
This should be made more explicit.
While the introduction section includes 'proteomics and metabolomics', the repositories hosting such data seems to be ignored in the work presented in this manuscript. Could the others clarify the reasons for restricting to INSDC resources?

Feedback: *We fully agree with your comment. When it comes to other omics datasets (e.g. proteomic and metabolomic), they can be described*

in an omics data paper, created in the "traditional" way: by filling in the relevant sections of the omics data paper template in Pensoft's ARPHA Writing Tool.

Changes in the manuscript: *We have revised the respective sections of the manuscript accordingly to show that the workflow is a prototype and only applies to genomic data from ENA. We have also clarified in the text that omics data papers can be authored in 2 different ways: manually (by filling in the template) and semi-automatically (prototyped by the genomic data workflow + additional manual effort).*

2. INSDC deposition comes first. What is the gain of the 'omics data paper' compared to referencing an INSDC accession number using the RRID mechanism in a standard article?

Feedback: *There are several advantages of publishing omics data papers: obtaining credit for the dataset, enabling early stage researchers and technicians to get recognition for their work even if it does not evolve into a traditional publication, improving metadata descriptions within the narrative, etc. Of course one can write a traditional article and simply reference the dataset identifier (an INSDC accession or other) but the data paper does not aim to describe a complete research study but only the data generation part of it. We see the data as a separate entity which can be described on its own, in addition to a traditional publication (see for detail Chavan & Penev 2011,* https://doi.org/10.1186/1471-2105-12-S15-S2*).*

3. automatic text generation from metadata harvested from public repositories. It would be nice if the authors could expand on the component. In particular, to show how a "Publication of data papers improves metadata quality".
Some data models used by ENA/SRA has a number of known shortcomings (e.g. impossibility to use ontology annotation, lack of objects to capture Study Design predictor variables, compression of Source-Sample graph).
It would be of interest to know if the authors have considered generating nanopublications from their workflow? If so, could it be documented and discussed in the manuscript?

Feedback: *Publication of data papers improves metadata quality as it provides opportunity for enhanced metadata descriptions. By following our template, authors are encouraged to describe their datasets in a very*

detailed manner. Most importantly, data papers are peer-reviewed and the datasets undergo data auditing by a professional data auditor at Pensoft. These practices aim at providing feedback to guide authors to improve their methodologies and/or the dataset descriptions themselves. Nanopublications are a research area in which we invest significant effort but the results will be published in a separate article.

Abstract:
Objective: clarify the origin of the import, ie "we created a workflow for streamlined import of omics metadata directly from public repositories into a data paper manuscript".

Changes in the manuscript: *We agree with this comment and have corrected the abstract.*

Introduction:
What does the "FAIRsharing of data" mean?

Changes in the manuscript: *We have corrected it to "sharing to FAIR data"*

Discussion:
point 3:
The authors posit that providing data article in addition to the dataset themselves enhance the ability to discover. In that section, it is unclear if the extended metadata provided in an omics data article supersedes or complements the data record held in the INSDC repository. Could the authors clarify if the ultimate goal is to update the INSDC metadata records ? If not, what is the mechanism provided by. 'omics data paper' to provide a standard interface to these new records? In particular, are these records "visible" to search engines (.e.g with schema.org mark up)?

Feedback: *The omics data paper complements the data record held in the INSDC repository. We are not aiming to replace such records but to improve their metadata descriptions through data paper publication. Please see the newly added Figure 1 which visualises this idea. Data paper publishing adds another layer of credibility to a dataset, since it includes peer-review. Our goal is not to update the INSDC metadata records although we would be open to collaborate if there is interest from their side. Data papers link to the original data records. The new interface we refer to is the narrative, which is yet another way to observe a*

dataset. It is more human-readable but since Pensoft publishes all its articles in JATS XML, it is also machine actionable, so a lot of the metadata can be understood by computers. We do not use schema.org markup yet.

Finally, when it comes to better visibility, publications are indexed by various search engines and academic research recommender systems, which means that they are more discoverable than the original datasets, stored in repositories. In addition, publications are often promoted in social media, which can additionally boost their visibility.

point 4:
Dryad, Figshare, Zenodo are generalist repositories which can mint DOI to various kind of digital objects, including datasets. Can the authors comment on the benefits of using a data paper over depositing to Zenodo or Figshare or Dryad?

Feedback: *Data paper publishing does not aim to provide another mechanism for dataset deposition but aims at improving the dataset description. Deposition to Dryad, Figshare and Zenodo, in addition to INSDC repositories, does not improve the dataset description through an enhanced metadata record (narrative) published in a way that provides scientific record,m credit, recognition and citation for all actors involved in data gathering, deposition and management.*

additional commenst:
versioning of schema, checklists. It would be nice to have the authors discuss that point as it has proven to be a tricky issue. How to keep in synch with ENA checklist as they evolve?

Feedback*: We will adapt the workflow accordingly if the ENA metadata schema changes. This is a part of the maintenance of the current workflow and its ability to work with the latest version of ENA.*

controled vocabularies and annotation. ENA is known to store variable values as string or numerical valuess but any semantic markup is somehow lost.
Can the authors comment on that point especially in the context of FAIR data and FAIR evaluation.

Feedback*: Unfortunately this is a common practice in other repositories probably due to technical difficulties to maintain the semantic markup. In Pensoft articles, data papers included, we implement upfront semantic markups for several terms (Especially taxon names). In the future, we plan to provide annotation of published texts to any domain specific ontology/vocabulary through the Pensoft Annotator tool.*

Table1: Data statistics section. SRA/ENA schemas cover "Assembly" and "Analysis". Have the authors considered pulling information from these documents?

Feedback: *We aimed to provide a more general template suited for other kinds of omics datasets besides genomics so we have not included Assembly and Analysis but the authors are encouraged to fill in details from these sections in the Data statistics and Data processing sections.*

Response to Reviewer 3 (Christine Ferguson) Minor revision

Dear Dr. Ferguson,

Thank you for taking the time to read and get an in-depth understanding of our manuscript! Your comments and suggestions encouraged us to resubmit the manuscript after making the necessary corrections. We are fully aware that the presented workflow is a prototype but we hope that it can demonstrate that interoperable data and metadata can easily be transformed into a rich metadata descriptor. We have made changes to the manuscript reflecting all comments we received and have created an installable version of the R shiny app as a golem package, as suggested be the first reviewer. We believe that these additions contribute to the reproducibility of your submission. You can find our replies to your comments below.

Kind regards,
Mariya Dimitrova
Lead author on behalf of all authors

Review and feedback:

Competing interests:
I declare that I have no competing interests

Comments to author:
The article describes the generation of a publishing workflow to generate data papers using metadata and identifiers gleaned from ENA data records (and any associated identifiers that appear in ArrayExpress or BioSamples). This provides authors with an automated and time-saving way to generate an 'omics data paper' to describe their previously deposited ENA datasets (which are used to populate the following sections: abstract, methods used to acquire the dataset, data resources, supplementary table containing imported BioSamples) . The workflow requires only a few article sections to be populated with the authors free text (Introduction/ reason for the study.; experimental design of the study, quantitative and qualitative description of the dataset, caveats, usage rights).

Positives:
The focus here is on generating research publications using 'omic' data and metadata. This is important as it is an example of data that is an early output of the research cycle, but often not all shared in the resulting publication output(s).
In the discussion the authors cite similar initiatives that transform data metadata into data articles and are clear about how their publishing workflow contributes to the field.
The article reports open source code in the form of the R shiny app, providing the potential for others to reproduce the workflow or to resume the JATS XML file outputs to generate their own 'omics' data papers.

As with similar tools/workflows that transform data metadata into articles:

This rewards early data deposition by researchers (ie ahead of publication);
It rewards researcher use of metadata standards and identifiers and renders the metadata more interoperable;
The rigorous review of the datasets that are included for publication would serve to enhance quality of datasets and completeness of metadata records and ensures they will be reusable; also educates authors in the correct use of standards for describing their data;
The ease of putting together the publication and increased discovery plus potential credit, will serve to incentivise researchers to publish their data and thereby embrace FAIR data

This article contributes to the spirit of open science by clearly acknowledging its contribution to what appears to be a busy publishing workflow sector, and by making its R shiny app codebase open source for use by others. The language used and clarity is good. Reviewers with technical/developer expertise will be required to vet the specifics around metadata extraction workflow, app deployment and codebase.

I have some minor comments for clarification and otherwise support publication of this article.

1. In the discussion, the authors describe how their approach compares with other tools and workflows in the data and metadata publishing

landscape. That there is overlap of intention/approach could also potentially be mentioned earlier in the article.

Feedback*: We agree and have made relevant additions in the penultimate paragraph of the Introduction.*

2. In a similar vein, the authors explain that the workflow described can process ENA metadata but not yet metadata from Genbank or DDBJ. The 'prototype' nature of the workflow could also perhaps be made clearer to readers closer to the beginning of the paper.

Feedback: *We agree with this comment and have made changes throughout the manuscript to specify that the workflow is a prototype.*

Response to Reviewer 1 Click here to access/download;Personal Cover;response_to_reviewer_1.docx

Response to Reviewer 1 (Yvan Le Bras) Reject

Dear Dr. Le Bras,

Thank you for the extensive review and useful comments! Your thorough comments and suggestions guided us to improve our manuscript and the R shiny app and to resubmit. We also want to thank you for your positive words on the importance of developing this kind of workflows and the approach we have used.

Please find enclosed inline below our feedback and account of the respective changes in the manuscript.

Respectfully,
Mariya Dimitrova

Review and feedback:

Competing interests:
I think I can have competing interests as I am working on a similar development and submit a project with similar development for funding. This is one reason why I prefer not giving any recommandation.

Comments to author:
This is a hot topic for me and as I am working on a very similar development, I prefer to say I have potentiel competing interest and not give any recommandation to editors. That being said, I think of interest to still review your article and provide my comments. I must also apologize for my approximate english and maybe the manner I wrote my review, quite rapidly even if I take times to read the article and test the related source code.

I really want to thank authors for this very interesting article, I think there is a lot of points who are very important, as trying creating a manner to incite researchers to create more rich metadata associated to their data. I also really think the overall proposed approach can be a good one, but I have serious doubts regarding the proposed operationalization, notably due to the poor information actually found in ENA metadata.

Feedback: *The aim of the data paper workflow is exactly to correct the deficiencies of metadata at ENA (or other repositories) by enhancing the metadata descriptions in the data paper and not just to import and publish ENA metadata as they are. The link between the permanent identifiers of the data (ProjectID) and the data paper (DOI) ensures the dataset is far better described with extended and peer reviewed metadata and is potentially far more re-usable to others.*

I finally choose the "reject" recomandation as it seems to me that 1/ the described workflow is not related to omics, but "just" genomics,

Feedback: *We agree that the workflow was elaborated and tested for genomic data, although analogous workflows for other kinds of omics metadata are relatively easy to implement.*

Changes in the manuscript: *We have amended the manuscript to reflect that the workflow is prototyped for genomic metadata, whereas the conceptualisation and template could be applied to omics data in a future work.*

2/ I think the described automated workflow can't really help to create "high-quality metadata records" or "encourages authors to improve the quality of their metadata to achieve a truly FAIR data wolrd".

Feedback*: The workflow is not just a plain conversion of the existing metadata but gives the authors the possibility to amend, edit and review the data paper manuscript once it is imported in the ARPHa Writing Tool (AWT) and further in the Biodiversity Data Journal (BDJ). Moreover, the authors can also create a data paper manually in AWT, by filling in the relevant sections of the data paper template where the link to the described data should be inserted as well.*

We agree that "high-quality" is an ambiguous term and have changed the manuscript to explain that we mean "more extensive, or extended" metadata. As previously stated, the omics data paper enables more thorough descriptions of metadata and also improves the visibility and findability of datasets. This is achieved through links between the dataset and the publication, which is indexed by

various research platforms and even social media. This raises the visibility and findability, thus the probability to comment on or reuse the dataset and related omics project to another level. The peer-review and data auditing processes bestow more credibility to the dataset, which is what we meant by "high-quality". Data auditing before publication is complementary to data curation performed by the data repositories and can help to uncover missing essential metadata records which ENA or another repository have let pass through their "curation filter". Finally, the described workflow does not claim to automatically improve metadata but rather to facilitate authors to describe their datasets more thoroughly with the help of the provided omics data paper template.

Changes in the manuscript: *We have made changes in several parts of the manuscript reflecting the above comment. We indicated that the workflow and omics data publishing in general aim to complement the existing practice of data deposition to recognised repositories, as demonstrated by the newly added Figure 1.*

Article

I really want to thank authors for this very interesting article, I think there is a lot of points who are very important, as trying creating a manner to incite researchers to create more rich metadata associated to their data. I also really think the overall proposed approach can be a good one, but I have serious doubts regarding the proposed operationalization, notably due to the poor information actually found in ENA metadata.

Feedback: *We agree with the reviewer's statement about the "poor information actually found in ENA metadata". Please see the first paragraph in the Feedback above where we justify our approach to improve the metadata quality through data papers.*

General comment

- "Omics data paper" seems to me not an appropriate manner to call it as it focus on genomics / genomics related data, and standards. In my opinion, such an omics data paper will propose a way to integrate at least genomics, proteomics and metabolomics data.

  Feedback: *We agree and have changed the term for the automated workflow described in the manuscript to "genomic". However, the*

concept and template are still applicable to all omics metadata. Authors can write manuscripts without the help of the conversion workflow in the ARPHA Writing Tool if they want to describe a non-genomic dataset.

- Did you look at ISAcommons and related ISAtools ? It seems to me this initiative and tools of interest to consider reaching goals you mention to create “”””real”””” (at least genomics/proteomics/metabolomics) omics data paper with rich metadata considering notably sampling methods or data processing.

  Feedback: *We are aware about the ISAcommons and ISAtools and recognise that they have similar aims to our project.*

  Changes in the manuscript: *We have included a discussion on ISAcommons and ISAtools in the* *3rd paragraph of the Introduction* *of the revised manuscript.*

- To incite data producers to better structure their data through rich metadata addition; I don’t think acting at the level of such genomics standard will be very efficient. Maybe it can be more useful to propose this at a “higher” (more federative) level as ISAcommons related tools and standards or others standards like EML, as they are already used by scientists adding relatively rich metadata.

  Feedback: *Use of community accepted standards, ISA included, and omics data paper publishing are complimentary. We do not aim to replace this important standard with another one, but we rather aim to create a publishing mechanism which stimulates authors to create extended metadata descriptors. In addition, Pensoft has already developed a workflow for import of EML metadata into manuscripts and successfully uses it in its routine publishing practice since 2010. The current project aims to replicate that workflow but for genomic data and more widely for other omics data in future.*

Abstract

- Looking at the abstract, it seems to me relatively a lack of relation between the Background and the Findings.

Changes in the manuscript: *We have revised all sections of the abstract to address the comment; those changes have been synchronised with the changes made to the rest of the manuscript.*

Introduction

- On "major infrastructures to handle higher-order biodiversity data" listed, DataOne is not cited, it seems to me this is one, with more than one millions data files for 77Tb

  Changes in the manuscript: *We agree with this comment and have cited DataONE as one of such major infrastructures in the* *<u>second paragraph of the Introduction.</u>* *Actually, since several years, Pensoft has integrated its EML conversion workflow to create "biodiversity"and "ecological" data papers with DataOne, to complement the already existing integration with GBIF and LTER.*

- Authors mention "community-accepted metadata standards" and in relation "GBIF uses the Darwin Core Standard" who is more a data standard than a metadata standard, used to create Darwin Core Archive containing data files where Darwin core terms are used to fill the headers, when a limited version of EML, the EML GBIF profile, is used as a metadata standard

  Changes in the manuscript: *We agree and fully understand that DwC is a data standard while EML is a metadata standard and have corrected it in the text (<u>second paragraph of the Introduction).</u>*

Methods

- Authors mention the identification of "high-level needs of the omics communities". This means common fields, or minimum shared information?

  Feedback*: We mean the best practice to publish high-quality metadata and not a standard-based solution. Actually, Pensoft has implemented conversion and import workflows for both EML to create data paper manuscripts (see Chavan and Penev 2011,*https://doi.org/10.1186/1471-2105-12-S15-S2*) and Darwin Core to import primary biodiversity data into manuscripts (see Smith et al 2013,*https://doi.org/10.3897/BDJ.1.e995)

***Changes in the manuscript**: We have made changes in the Approach subsection of the Methods section to clarify the difference between EML and Darwin Core.*

- Testing the "RStudio cloud" based Shiny app, it seems to me that the example ENA "PRJDB2900" is maybe not a good one as it is really poor in terms of metadata, but maybe this point a major issue as metadata is often very poor on such kind of repository, and linked to this kind of standard. Notably a lack in "sampling method", ""only"" "sample_name", "strain", "dev_stage", "genotype" and "host_body_site", and nothing in "Environmental profile", "Geographic range", nothing in "Data Processing".

  ***Feedback**: You are correct that this points to a major issue but there is also the fact that not all sections of the omics data paper template are meant to be automatically filled in by the workflow. Many of them are meant to be filled in manually and the authors have the rights and the option to include this missing information in the relevant sections of the data paper manuscript.*

  ***Changes in the manuscript**: We have made changes to the manuscript to reflect that (in first paragraph of section* "Workflow for extracting relevant metadata from ENA XML files" and the second paragraph of "Integration of metadata extraction workflow with the ARPHA Writing Tool" section)

- Related to previous comment, authors mention the fact that "certain sections" "were made mandatory" but if content is as poor as the content seen on the example accession, it seems to me that this is not very useful.

  ***Feedback:** We agree that it is not entirely clear what is meant by "made mandatory".*

  ***Changes in the manuscript**: We have clarified that under "mandatory" we meant "mandatory in ARPHA" for the manuscript to proceed to review. We have clarified that in the second paragraph of the "Integration of metadata extraction workflow with the ARPHA Writing Tool" section.*

- Authors propose creation of a “long format table” from retrieved BioSamples metadata, I suppose because a CSV file is more comfortable for users without programming knowledge, but it seems to me this format with “blocks of lines” by BioSample who can have different number of lines from one block to another is not really easy to deal with and maybe can be at the origin of error.

  Feedback: *Long format tables in such universally used format as CSV can easily be transformed to wide format tables in many of the data processing softwares.*

Findings

- Totally agree with your sentence “The described dataset is at the core of the data paper, but the methodology required to obtain it is just as valuable as the data itself.
- Regarding Table 1, some questions come to my mind:
- How many records on ENA have information related to “environmental and geographic characteristics of the locations where their material was collected”?

  Feedback: *We have no information regarding the completeness of ENA metadata records across the different projects. It may well be that most of the datasets lack this kind of information. On the other side, this is exactly what the workflow offers, that is the authors are encouraged to supplement imported metadata with additional information, such as the abovementioned environmental and geographic characteristics during the process of completion of their manuscripts already in the ARPHA Writing Tool environment.*

- Same question for “Technologies used” / “ Sample processing” and “Data Processing”.

  Feedback: *Some ENA records do contain information about sequencing technologies which could go in the “Technologies used” section of the “Sample processing” section. For the rest you are correct that there are no ENA records but authors can fill those sections manually in the ARPHA Writing Tool, thus improving the description of their datasets deposited in ENA.*

- Linked to previous questions, is there a manner to update existing ENA records to add metadata information?

***Feedback**: The way metadata is updated at ENA is ENA's own policy, what we do is to enhance records in the data paper completed in the AWT. In a starting EU-funded project, coordinated by Pensoft, one of the tasks of the EMBL EBI as a partner is to provide means for easy feedback, annotation and improvement of ENA's metadata records.*

- Regarding "Biodiversity profile", "written by the authors" seems to be too bad regarding others Metadata standards and/or terms who can help filling that, notably EML. Moreover, controlled vocabulary can be used to help enrich metadata content, notably Gene Ontology. It appears to me of interest to consider proposing the users using terminological resources notably for taxonomic information.

  ***Feedback**: We agree that it is recommended to use existing standards and vocabularies, so we have added a short paragraph outlining this recommendation in the end of the section "Structure of the OMICS data paper". Authors can attach a supplementary EML file or create their own Linked data table[1], which could serve a similar purpose and still use ontologies to express biodiversity data.*

  The "biodiversity profile" has indeed to be complemented by the authors, however it follows the EML metadata profile used by GBIF and also by Pensoft to create "biodiversity data paper". The process is described in due detail in Chavan and Penev (2011, https://doi.org/10.1186/1471-2105-12-S15-S2) and Penev et al (2017, https://doi.org/10.3897/rio.3.e12431).

- Data resources //XREF_LINK// seems to me not dedicated to inform about a data resource, but more the use of a generic xref term. Isn't it?

  ***Changes in the manuscript**: We have specified that we refer to a //XREF_LINK which has attribute ENA-FASTQ-FILES so it is not any generic xref term but the one linking to the data resource.*

---

[1] *https://blog.pensoft.net/2020/04/24/how-to-get-data-from-research-articles-back-into-the-research-cycle-%D0%B0t-no-additional-costs/*

- Concerning “Data statistics”, again it would be better to avoid asking the user filling it by hand, is there any manner to propose a way to populate this section from existing metadata fields or other machine actionable resources?

  Feedback*: We give authors the freedom to represent their data statistics in the way they see most valuable and informative to the readers. During the peer-review process they can obtain feedback about the data statistics they have decided to include.*

- Concerning Usage rights, authors mention the fact that open access is default value, can you precise which license? To facilitate reuse of related information and OA initiatives, authors can also choose to only accept OA license, but here it seems this is not the case.

  Feedback*: We have inserted in the manuscript a link to Pensoft’s policy regarding data licenses and usage rights outlined in https://riojournal.com/article/12431/instance/3306534/. For data, Pensoft recommends the following data publishing licenses:*

  - *Open Data Commons Attribution License http://www.opendatacommons.org/licenses/by/1.0/*
  - *Creative Commons CC-Zero Waiver http://creativecommons.org/publicdomain/zero/1.0/*
  - *Open Data Commons Public Domain Dedication and License http://www.opendatacommons.org/licenses/pddl/1-0/*

  however it is the decision of the authors and a matter of the data repositories which license to use. The data paper itself is published under the Creative Commons Attribution LIcense (CC-BY 4.0), https://creativecommons.org/licenses/by/4.0/

- Authors mention that “some data paper sections do not have ENA metadata fields associated with them”, is some exact? Maybe many can be more appropriate isn’t it?

  Feedback*: We agree and have corrected it in the manuscript.*

- Authors mention “and the authors are encouraged to fill in their contents”. Are authors encouraged to fill in their contents in the

original ENA record? Or on related data paper fields ? And/or is there any feedback planned between data papers and ENA so ENA records can be updated from a data paper creation process?

***Feedback**: Authors are encouraged to provide complete metadata records during the submission process to ENA or afterwards if they want to correct them. They can also create extended metadata descriptions in the omics data paper.*

***Changes in the manuscript:** We have clarified this in the first paragraph of the Findings section. We are in contact with ENA and have proposed to them to link to the data papers describing their datasets.*

- Understanding Figure 1 was not so easy for me. The image quality of the Figure 1 is weak. ArrayExpress is not displayed explicitly on the Figure 1. Maybe the arrow between “ENA Sample” and “BioSamples” has to be dotted as the link is not always there?

  ***Changes in the manuscript**: We have improved the quality of the figure, which is now Figure 2 because we added a different first figure that demonstrates how data papers complement existing methods for metadata sharing.*

- Looking at the statement “thus enhancing manuscripts with high quality metadata”, can the authors explain what is “high quality metadata”? Did the authors use metrics to evaluate that, for example metrics related to metadata completeness or others? It seems to me high quality metadata can be related to rich and detailed metadata and semantically connected to meaningful resources for example, and I am not sure the presented system is in line with this.

  ***Feedback**: We agree that 'high-quality' is not well defined term and accept your interpretation that “high-quality” metadata means actually “rich and detailed metadata, semantically connected to meaningful resources”.*

  ***Changes in the manuscript**: We have changed the use of “high-quality” to “extended” or 'enhanced' metadata throughout the manuscript.*

- Authors “promote the reuse and interoperability of MIxS compliant metadata sourced from BioSamples”, is this sufficient, notably to publish a quality data paper?

  Feedback*: It may not be sufficient but it is certainly beneficial to promote them to the community.*

- I particularly appreciate the JATS transformation functionality

Discussion

- Authors mention “the average number of published data papers continues to grow (Fig. 3)”, but looking at the Figure 3, it seems to me that there is like a plateau since 2015 no? It will be interesting to look at such a graph with this number normalized regarding data sharing effort.

  Changes in the manuscript*: We agree with the comment and have corrected it in the text to highlight the data paper publishing uptake. We have also removed Figures 3 and 4, which we found to be a bit superfluous in the Discussion section.*

- In the “Comparison with other tools and workflows” sub section, authors mention EML in the first paragraph. Just to be sure the reader can’t think this paragraph is related to the EML metadata standard, but to the limited version used by GBIF, it seems to me of interest to specify “EML GBIF profile”.

  Changes in the manuscript*: Thank you for pointing it out, we have corrected it in the manuscript.*

- In the “Data papers for the field of omics: rationale and benefits” sub section first paragraph, this argue to me to look at others initiatives / standards than thus proposed by ENA, notably to go beyond the data, to publish tools, protocols and others Research products in data paper.
- Regarding “Data papers for the field of omics: rationale and benefits” first point, authors mention “the introduction of data scientists into the publishing process ensures that submitted data and metadata are FAIR”. It seems to me, unfortunately, that the

use of “ensure” is maybe not useable or this ensures a minimal level of FAIRness maybe.

***Changes in the manuscript**: we agree that the word 'ensures' is too strong but we definitely encourage and support authors with our workflow to FAIRizise their data to the maximum possible extent. We have corrected the manuscript to clarify that it “ensures to an extent”.*

- Double checking seems to be a big effort here for a limited impact no?

  ***Feedback**: The data auditing effort (QA/QC) is substantial indeed but proved to be valuable and very useful in improving the data and metadata quality and reuse.*

- Regarding “Data papers for the field of omics: rationale and benefits” second point, authors mention “we came across many datasets with missing or incorrectly formatted metadata fields”. Considering the manner researchers are filling ENA metadata, this is really not a surprise for me. That’s a reason why I think that maybe focusing on ENA metadata is not a so good idea unless the system allows to help filling retroactively ENA metadata fields.

  ***Feedback:** The retroactive filling of ENA metadata depends on ENA policies, what we do is just to improve the metadata in a third party environment through writing, peer review and publication, linked back to the original ENa metadata record.*

- “Annotaire tool” written instead of “Annotare tool” if I am not wrong.

  ***Changes in the manuscript:** We have corrected it in the text.*

- Regarding “Limitations and future outlooks” section, it seems to me that the described automated workflow works only with ENA metadata records is a huge limitation.

  ***Changes in the manuscript:** We agree and revised the manuscript to state that the automated conversion workflow is a working prototype based on ENA’s metadata profiles.*

- Regarding “Limitations and future outlooks” section, I am not sure that motivating the choice of ENA because their metadata structure was easiest to work with is a good point.

  Feedback: *We agree. Our motivation was that the links between ENA, BioSamples and ArrayExpress are more clear or in other words easy to “translate” into XPATH and HTTP requests. We think that it is fair to look for the least-complicatedly structured source of metadata when building a prototype workflow. We apply this “minimal viable product” concept because to us it is important to receive feedback and monitor the uptake of the workflow and then make changes to the future versions.*

- Regarding “Limitations and future outlooks” section and the “two-way link” statement, again it seems to me this argue towards the use of another kind of metadata standard, domain-oriented for example and/or another kind of data repository that can refer to ENA files.
-

  Feedback: Well, this is what we have at disposal. This metadata standard may not be ideal but this is what scientists and data repositories use.

OMICS data papers Shiny apps

Interesting points

- Easy and quick to use, with a nice display (choice of color palettes, simple UI)
- Ability to download the files in local with format choice
- A commented and legible code !
- Access to reference and general context from the app

Enhancements

- Turn it into a package of it (cf. {golem})
- Add an installation script (get all required packages). See this link for auto-install packages: https://stackoverflow.com/questions/4090169/elegant-way-to-check-for-missing-packages-and-install-them
- Test with the provided example:
  - use {shinycssloaders} while loading paper
  - avoid empty fields: fill them with a shiny::helpText("No content found") OR do not display them.

    - maybe a way to get to an index of references?
- Code itself:
    - split tasks in subroutines: this allows to use *apply() instead of for() which is more legible and somehow faster and makes bug detection better, rather than “Error on line 579 of app.R”
    - package namespacing (pkg::function) not always applied (e.g. app.R#234 vs app.R#236)
    - use “document outline” if you use RStudio: allows to browse the code quickly and efficiently
- Add a way to browse the downloaded document
    - check for {shinyTree}. A suggestion could be:
    - make a hierarchy of displayed <div> elements (for instance, in Newick format for “Value of the dataset” and its subitems: {Value of the dataset, {Scientific value, Societal value}})
    - when user click on a level of the tree, redirect him to the <div> with attribute ‘id’ matching the clicked level
- Add a Help to explain:
    - how the downloaded document will be structured
    - some definitions and basic ENA concepts (same example: what are the “scientific value” and “societal value”)

Changes in the manuscript*: Thank you for the useful suggestions! We have created a golem package: https://github.com/pensoft/omicsdatapaper. It allows running the R shiny app after local installation of the package. Instructions for installation and running are available on github: https://github.com/pensoft/omicsdatapaper/blob/main/README.md.*
The package imports all required packages and we’ve fixed package namespacing. We’ve revised the code and have replaced the use of for() loops with lapply() where appropriate. We have also added additional documentation / help about the omics data paper structure. It is accessible by clicking the “Read more” button in “Omics data paper structure”. The previous R shiny app code is still available on GitHub and we’ve added a script to it to load all required packages.

Response to Reviewer 2 Click here to access/download;Personal Cover;response_to_reviewer_2.docx

Response to Reviewer 2 (Philippe Rocca-Serra) Major revision

Dear Dr. Rocca-Serra,

Thank you for taking the time to read and get an in-depth understanding of our manuscript! Your comments and suggestions encouraged us to resubmit the manuscript after making the necessary corrections. We have specified that the workflow is a prototype, hence it currently works with genomic metadata only. We indicate that the workflow and omics data publishing in general do not aim to replace the existing practice of data deposition to recognised repositories, but aim to complement it. We have made changes to the manuscript reflecting all comments we received and have created an installable version of the R shiny app as a golem package, as suggested be the first reviewer. We believe that these additions contribute to the reproducibility of your submission. You can find our replies to your comments below.

Kind regards,
Mariya Dimitrova
Lead author on behalf of all authors

Review and feedback:

Competing interests:
Lead on the Datascriptor project mentioned in the manuscripts

Comments to author:
I thank the authors for a well written and well presented manuscript.

main comments:
1. slight over-claiming when it comes to 'omics'
What the authors have documented as "Omics Data Papers" would benefit from being highlighted as 'sequencing based omics data sets'.
since the targeted repositories are restricted to sequencing based omics and the workflows essentially interrogates insdc resources, thus covering Transcriptomics, Genomics, metagenomics, but leaving out proteomics and metabolomics.
This should be made more explicit.

While the introduction section includes 'proteomics and metabolomics', the repositories hosting such data seems to be ignored in the work presented in this manuscript. Could the others clarify the reasons for restricting to INSDC resources?

Feedback: *We fully agree with your comment. When it comes to other omics datasets (e.g. proteomic and metabolomic), they can be described in an omics data paper, created in the "traditional" way: by filling in the relevant sections of the omics data paper template in Pensoft's ARPHA Writing Tool.*

Changes in the manuscript: *We have revised the respective sections of the manuscript accordingly to show that the workflow is a prototype and only applies to genomic data from ENA. We have also clarified in the text that omics data papers can be authored in 2 different ways: manually (by filling in the template) and semi-automatically (prototyped by the genomic data workflow + additional manual effort).*

2. INSDC deposition comes first. What is the gain of the 'omics data paper' compared to referencing an INSDC accession number using the RRID mechanism in a standard article?

Feedback: *There are several advantages of publishing omics data papers: obtaining credit for the dataset, enabling early stage researchers and technicians to get recognition for their work even if it does not evolve into a traditional publication, improving metadata descriptions within the narrative, etc. Of course one can write a traditional article and simply reference the dataset identifier (an INSDC accession or other) but the data paper does not aim to describe a complete research study but only the data generation part of it. We see the data as a separate entity which can be described on its own, in addition to a traditional publication (see for detail Chavan & Penev 2011,* https://doi.org/10.1186/1471-2105-12-S15-S2*).*

3. automatic text generation from metadata harvested from public repositories. It would be nice if the authors could expand on the component. In particular, to show how a "Publication of data papers improves metadata quality".
Some data models used by ENA/SRA has a number of known shortcomings (e.g. impossibility to use ontology annotation, lack of objects to capture Study Design predictor variables, compression of Source-Sample graph).

It would be of interest to know if the authors have considered generating nanopublications from their workflow? If so, could it be documented and discussed in the manuscript?

Feedback: *Publication of data papers improves metadata quality as it provides opportunity for enhanced metadata descriptions. By following our template, authors are encouraged to describe their datasets in a very detailed manner. Most importantly, data papers are peer-reviewed and the datasets undergo data auditing by a professional data auditor at Pensoft. These practices aim at providing feedback to guide authors to improve their methodologies and/or the dataset descriptions themselves. Nanopublications are a research area in which we invest significant effort but the results will be published in a separate article.*

Abstract:
Objective: clarify the origin of the import, ie "we created a workflow for streamlined import of omics metadata directly from public repositories into a data paper manuscript".

Changes in the manuscript: *We agree with this comment and have corrected the abstract.*

Introduction:
What does the "FAIRsharing of data" mean?

Changes in the manuscript: *We have corrected it to "sharing to FAIR data"*

Discussion:
point 3:
The authors posit that providing data article in addition to the dataset themselves enhance the ability to discover. In that section, it is unclear if the extended metadata provided in an omics data article supersedes or complements the data record held in the INSDC repository. Could the authors clarify if the ultimate goal is to update the INSDC metadata records ? If not, what is the mechanism provided by. 'omics data paper' to provide a standard interface to these new records? In particular, are these records "visible" to search engines (.e.g with schema.org mark up)?

Feedback: *The omics data paper complements the data record held in the INSDC repository. We are not aiming to replace such records but to*

improve their metadata descriptions through data paper publication. Please see the newly added Figure 1 which visualises this idea. Data paper publishing adds another layer of credibility to a dataset, since it includes peer-review. Our goal is not to update the INSDC metadata records although we would be open to collaborate if there is interest from their side. Data papers link to the original data records. The new interface we refer to is the narrative, which is yet another way to observe a dataset. It is more human-readable but since Pensoft publishes all its articles in JATS XML, it is also machine actionable, so a lot of the metadata can be understood by computers. We do not use schema.org markup yet.

Finally, when it comes to better visibility, publications are indexed by various search engines and academic research recommender systems, which means that they are more discoverable than the original datasets, stored in repositories. In addition, publications are often promoted in social media, which can additionally boost their visibility.

point 4:
Dryad, Figshare, Zenodo are generalist repositories which can mint DOI to various kind of digital objects, including datasets. Can the authors comment on the benefits of using a data paper over depositing to Zenodo or Figshare or Dryad?

Feedback: *Data paper publishing does not aim to provide another mechanism for dataset deposition but aims at improving the dataset description. Deposition to Dryad, Figshare and Zenodo, in addition to INSDC repositories, does not improve the dataset description through an enhanced metadata record (narrative) published in a way that provides scientific record,m credit, recognition and citation for all actors involved in data gathering, deposition and management.*

additional commenst:
versioning of schema, checklists. It would be nice to have the authors discuss that point as it has proven to be a tricky issue. How to keep in synch with ENA checklist as they evolve?

Feedback*: We will adapt the workflow accordingly if the ENA metadata schema changes. This is a part of the maintenance of the current workflow and its ability to work with the latest version of ENA.*

controled vocabularies and annotation. ENA is known to store variable values as string or numerical valuess but any semantic markup is somehow lost.
Can the authors comment on that point especially in the context of FAIR data and FAIR evaluation.

Feedback*: Unfortunately this is a common practice in other repositories probably due to technical difficulties to maintain the semantic markup. In Pensoft articles, data papers included, we implement upfront semantic markups for several terms (Especially taxon names). In the future, we plan to provide annotation of published texts to any domain specific ontology/vocabulary through the Pensoft Annotator tool.*

Table1: Data statistics section. SRA/ENA schemas cover "Assembly" and "Analysis". Have the authors considered pulling information from these documents?

Feedback: *We aimed to provide a more general template suited for other kinds of omics datasets besides genomics so we have not included Assembly and Analysis but the authors are encouraged to fill in details from these sections in the Data statistics and Data processing sections.*

Click here to access/download;Personal Cover;response_to_reviewer_3.docx

Response to Reviewer 3 (Christine Ferguson) Minor revision

Dear Dr. Ferguson,

Thank you for taking the time to read and get an in-depth understanding of our manuscript! Your comments and suggestions encouraged us to resubmit the manuscript after making the necessary corrections. We are fully aware that the presented workflow is a prototype but we hope that it can demonstrate that interoperable data and metadata can easily be transformed into a rich metadata descriptor. We have made changes to the manuscript reflecting all comments we received and have created an installable version of the R shiny app as a golem package, as suggested be the first reviewer. We believe that these additions contribute to the reproducibility of your submission. You can find our replies to your comments below.

Kind regards,
Mariya Dimitrova
Lead author on behalf of all authors

Review and feedback:

Competing interests:
I declare that I have no competing interests

Comments to author:
The article describes the generation of a publishing workflow to generate data papers using metadata and identifiers gleaned from ENA data records (and any associated identifiers that appear in ArrayExpress or BioSamples). This provides authors with an automated and time-saving way to generate an 'omics data paper' to describe their previously deposited ENA datasets (which are used to populate the following sections: abstract, methods used to acquire the dataset, data resources, supplementary table containing imported BioSamples) . The workflow requires only a few article sections to be populated with the authors free text (Introduction/ reason for the study.; experimental design of the

study, quantitative and qualitative description of the dataset, caveats, usage rights).

Positives:
The focus here is on generating research publications using 'omic' data and metadata. This is important as it is an example of data that is an early output of the research cycle, but often not all shared in the resulting publication output(s).
In the discussion the authors cite similar initiatives that transform data metadata into data articles and are clear about how their publishing workflow contributes to the field.
The article reports open source code in the form of the R shiny app, providing the potential for others to reproduce the workflow or to resume the JATS XML file outputs to generate their own 'omics' data papers.

As with similar tools/workflows that transform data metadata into articles:

This rewards early data deposition by researchers (ie ahead of publication);
It rewards researcher use of metadata standards and identifiers and renders the metadata more interoperable;
The rigorous review of the datasets that are included for publication would serve to enhance quality of datasets and completeness of metadata records and ensures they will be reusable; also educates authors in the correct use of standards for describing their data;
The ease of putting together the publication and increased discovery plus potential credit, will serve to incentivise researchers to publish their data and thereby embrace FAIR data

This article contributes to the spirit of open science by clearly acknowledging its contribution to what appears to be a busy publishing workflow sector, and by making its R shiny app codebase open source for use by others. The language used and clarity is good. Reviewers with technical/developer expertise will be required to vet the specifics around metadata extraction workflow, app deployment and codebase.

I have some minor comments for clarification and otherwise support publication of this article.

1. In the discussion, the authors describe how their approach compares with other tools and workflows in the data and metadata publishing landscape. That there is overlap of intention/approach could also potentially be mentioned earlier in the article.

***Feedback**: We agree and have made relevant additions in the penultimate paragraph of the Introduction.*

2. In a similar vein, the authors explain that the workflow described can process ENA metadata but not yet metadata from Genbank or DDBJ. The 'prototype' nature of the workflow could also perhaps be made clearer to readers closer to the beginning of the paper.

***Feedback:** We agree with this comment and have made changes throughout the manuscript to specify that the workflow is a prototype.*
