# Supplementary material for: A streamlined workflow for conversion, peer review, and publication of genomics metadata as omics data papers
Source: Gigascience. 2021 May 13;10(5):giab034. doi: 10.1093/gigascience/giab034 (PMC8117446; doi:10.1093/gigascience/giab034)
Supplement: giab034_GIGA-D-20-00352_Revision_1 [file giab034_giga-d-20-00352_revision_1.pdf]

GigaScience

A streamlined workflow for conversion, peer review and publication of genomics metadata as Omics Data Papers

--Manuscript Draft--

**Manuscript Number:**	GIGA-D-20-00352R1
**Full Title:**	A streamlined workflow for conversion, peer review and publication of genomics metadata as Omics Data Papers
**Article Type:**	Technical Note
**Funding Information:**	H2020 Marie Skłodowska-Curie Actions (764840) — Ms Mariya Dimitrova<br>Pensoft Publishers — Mr Georgi Zhelezov, Mr Seyhan Demirov
**Abstract:**	Background<br>Data papers have emerged as a powerful instrument for open data publishing, obtaining credit, and establishing priority for datasets generated in scientific experiments. Academic publishing improves data and metadata quality through peer-review and increases the impact of datasets by enhancing their visibility, accessibility, and re-usability.<br>Objective<br>We aimed to establish a new type of article structure and template for omics studies: the omics data paper. To improve data interoperability and further incentivise researchers to publish well-described data sets, we created a prototype workflow for streamlined import of genomics metadata from the European Nucleotide Archive directly into a data paper manuscript.<br>Methods<br>An omics data paper template was designed by defining key article sections which encourage the description of omics datasets and methodologies. A metadata import workflow, based on REpresentational State Transfer services and Xpath, was prototyped to extract information from the European Nucleotide Archive, ArrayExpress and BioSamples databases.<br>Findings<br>The template and workflow for automatic import of standard-compliant metadata into an omics data paper manuscript provide a mechanism for enhancing existing metadata through publishing.<br>Conclusion<br>The omics data paper structure and workflow for import of genomics metadata help to bring genomic and other omics datasets into the spotlight. Promoting enhanced metadata descriptions and enforcing manuscript peer review and data auditing of the underlying datasets brings additional quality to datasets. We hope that streamlined metadata re-use for scholarly publishing encourages authors to create enhanced metadata descriptions in the form of data papers to improve both the quality of their metadata and its findability and accessibility
**Corresponding Author:**	Mariya Dimitrova<br>Pensoft Publishers<br>Sofia, BULGARIA
**Corresponding Author Secondary Information:**	
**Corresponding Author's Institution:**	Pensoft Publishers
**Corresponding Author's Secondary Institution:**	
**First Author:**	Mariya Dimitrova
**First Author Secondary Information:**	
**Order of Authors:**	Mariya Dimitrova

Powered by Editorial Manager® and ProduXion Manager® from Aries Systems Corporation

	Raïssa Meyer
	Pier Luigi Buttigieg
	Teodor Georgiev
	Georgi Zhelezov
	Seyhan Demirov
	Vincent Smith
	Lyubomir Penev
**Order of Authors Secondary Information:**	
**Response to Reviewers:**	Dear Editor and Reviewers,<br><br>Thank you for taking the time and effort to review our manuscript again! We highly appreciate your constructive feedback in this and the previous review rounds. Your thoughtful comments helped us make some important changes.<br><br>You advised us to submit the R shiny app to https://bio.tools and https://SciCrunch.org databases to receive RRID (Research Resource Identification Initiative ID) and biotoolsID identifiers and we have done so. The identifiers are: biotools:omics-data-paper-shinyapp-golem for biotools and SCR_019809 for SciCrunch.org. We have updated the manuscript with these identifiers in the "Availability of supporting source code and requirements" section on page 28.<br><br>Below you will find an explanation of all other modifications we made to the manuscript, as well as detailed responses to your comments. You can read our response also as a document, which is attached to our resubmission.<br><br>Question 1: Do data paper undergo peer review?<br>if so, what is the acceptance rate?<br>Scientists claims that they are over burden with data management tasks, from data deposition to manuscript writing.<br>Could the authors indicate how much work a data paper represents in contrast to a classic scientific article?<br><br><br>Response:<br><br>Similarly to all other manuscript types in Pensoft journals, all data papers undergo a rigorous peer review (see https://bdj.pensoft.net/about#DataReviewGuidelines). In addition, prior to peer review they are also subjected to data auditing by a data auditor at Pensoft who makes sure that data is accessible and its quality is of high standard according to a data quality checklist (https://bdj.pensoft.net/about#DataQualityChecklistandRecommendations). The submitted manuscript contains information about the peer review and data audit of the genomics data papers. We have indicated Pensoft's data auditing and peer-review processes in the "Omics data papers and underlying datasets undergo peer-review and data auditing" subsection of the Discussion section on page 23.<br><br>The acceptance rate of the Biodiversity Data Journal varies between 70-80%.<br><br>In terms of the effort that data papers require from authors, it really depends on the nature of the data and the author's preference for including a certain number of data statistics and meta-analyses to describe their data. Data papers do not require authors to include an analysis of the dataset but they can do so if they wish, as long as the data paper does not become too long-drawn-out. The key aspect of a data paper is the detailed description of the data following the community accepted standards and FAIRness and quality of the data itself, therefore authors' main efforts should go towards making sure that the described datasets are consistent throughout, well-described in terms of methodology and openly available and accessible. The goal of the data paper is to make the dataset fully reusable so that other researchers can repeat the experiment or collate the published data with other datasets to generate

new hypotheses and research results.

The benefits of publishing data papers are listed extensively in the section “Data papers for the field of omics: rationale and benefits” (page 22) of the manuscript. In the first version of the manuscript, we had included a figure (Please see the attached cover letter for Fig. 1) which shows a clear increasing trend in popularity and use of data papers as a useful method of data publishing and a powerful instrument for improving research integrity and data reuse. For example the Biodiversity Data Journal alone, shows the following numbers in publication of data papers: 2018 (9), 2019 (31). For 2020 (not pictured in the figure), there were 68 data papers, which indicates an yearly increase of more than 100 % in the last year alone.

Question 2: Following creation of the populated backbone, how much write-up are authors expected to do to reach minimal content and quality requirements?
I understand the authors somehow tackle this issue on pages 22-26 but I am still concerned about the economics and sustainability of the approach.
The authors indicate that OMIC data paper are audited, which means curation time is expanded. This is an expensive task to accomplish and requires domain experts.
It would be interesting to cover some of these aspects in the discussion, all the more so finding reviewers is becoming harder and harder (which would be also require finding data experts).

Response:

Data auditing is indeed an expensive task but Pensoft strives towards publishing high quality datasets and has fully committed to this practice, provided by in-house data auditors. The data auditors are biologists with extensive experience with data handling, storing and processing. They ensure that the datasets follow some basic guidelines to ensure their FAIRness and consistency. They provide authors with tips on how to make their data FAIR, if it is not FAIR yet. Data auditors do not reproduce data generation and analysis if there is such. This would be the role of peer-reviewers. Finally, data auditors are permanently employed and unlike peer-reviewers do not have to be sought out on a case by case basis, which significantly reduces the waiting time. Therefore, we cannot really compare the process of finding reviewers with the process of finding data auditors. We have included a paragraph in the manuscript about how the system works (“Omics data papers and underlying datasets undergo peer-review and data auditing” subsection on page 23), with the relevant citations of the respective data quality guidelines of the Biodiversity Data Journal,. Perhaps, the intricacies of data paper publishing, including finding data auditors, can be explored in more detail in a separate publication but here we wanted to focus on genomic data papers and the workflow. More information about the data auditing process at Pensoft can be found in this blog (also cited in the discussion part of the paper):
https://www.eurekalert.org/pub_releases/2019-10/pp-aif101819.php

Question 3: Is there a specific mechanism used in Pensoft OMIC Data Paper to reference database accession numbers so those can be easily identified and extracted?

Response:

Database accession numbers have to be referenced in the Data Resources section of the omics data paper (Please see the attached cover letter for Fig. 2). The Resource identifier field, where such an accession number should be entered, is a required field of the template, meaning that it cannot be submitted without being filled in. If using the omics data paper conversion workflow, this field is automatically populated with an ENA sequence identifier.

After publication, all articles in BDJ are publicly available also as JATS XML and submitted as such for archiving and display to PubMedCentral, in addition to the traditional PDF and HTML formats. Therefore, all identifiers and links to external databases are marked up in the XML version of the article and can then be indexed by Web crawlers, including Pensoft’s own RDFization scripts which transform XML articles to Resource Description Framework (RDF) to ensure machine readability of the text

Powered by Editorial Manager® and ProduXion Manager® from Aries Systems Corporation

(Penev et al., 2019).

Question 4: Have the authors also considered an automated action to update a database (e.g. ENA) record with a DOI associated with a Pensoft `OMIC data paper`? This comes back to the issue of producing an entirely new artifact, possibly one containing 90 % of the information stored in a SRA XML document but presented in a JATS documents.
Were EMBL-EBI records to be given DOIs, would it impact the OMIC Data Paper model ?

Response: We have considered this possibility as an excellent way to link data and literature both ways. There were some preliminary discussions with ENA to at least provide a back linking from the published data paper to the relevant resource at ENA and we expect to achieve that during the starting EU-funded BiCIKL project, coordinated by Pensoft. During that project, EMBL-EBI should provide an interface to edit/improve the metadata of already submitted datasets from annotations to these, data papers included, posted post factum.

Pensoft has a proven experience in this kind of workflow with GBIF, For example, the discrepancies in data already indexed in GBIF found by our data auditors are sent to the authors with a clear demand to correct the data in the GBIf indexed dataset before the manuscript will be forwarded to peer review in the journal.

If EMBL-EBI records are given (DataCite) DOIs, which of no doubt would be an excellent practice, this would not create any issues for the workflow because they would be just different identifier records than the data papers. This is because the original dataset is a separate entity than the data paper in which it is described.

Question 5: Dealing with large datasets.

Some studies may contain hundreds, thousands of samples. The interaction with the AWT indicates that the OMIC data article would contain a long list of biomaterials, and associated resources.

Have the authors considered aspect of stacking / compressing information or have they considered automated sentence generation to assist authors in expanding the text for an OMIC data paper ?

Response:

We have thought about large datasets and this is why we attach the BioSamples checklist as a supplementary file and not as a table within the narrative itself. When it comes to sentence generation, this is not really needed because we do not require separate description of each and every sample. Authors can aggregate their description of large datasets to create a concise and clear manuscript.

Actually, the sample size does not directly affect the size of the data paper, because the data are stored outside the journal's infrastructure, which hosts the narrative of the data paper. Large data sets would require a bit more detailed text description and explanations, however this is a normal practice in the manuscript authoring and publishing process.

Question 6. License on the data

Shouldn't there be a filled in section in the OMIC data paper to specific the nature of the license under which data and resources are made available?
this is possible to auto-generated from public repositories and, if authors create a manuscript from scratch, they should be offered options.
This is distinct from the license attached to the manuscript itself or to the metadata.

Response: There is already such a section, called "Usage rights", where authors can specify the license of the data. It is implemented both in Pensoft's ARPHA template

and in the R shiny app (Please See the attached cover letter for Fig. 3).

We have not implemented a dropdown menu or other kind of entry form with limited options for licenses because there might be a case in which different datasets, described in the data paper, have different (open-source) licenses so it would be best if the authors specify that manually. As stated in the author guidelines of BDJ, only datasets with open-source licenses can be published as part of data paper publications in Pensoft's journals (Please see the attached cover letter for Fig. 4).

----
minor corrections:

page 5, "MIxS consists of three checklists, <comma inserted> each containing several packages for the description of various environments where genomic material could be sampled from [22]."

- >overall, check for similar missing elements of punctuation.

Response:
We have corrected this mistake and have checked for other punctuation mistakes throughout the manuscript.

page 6: "A more comprehensive approach towards omics metadata mobilisation is undertaken
by the ISA Commons community [28], who ",

-> s/who/which/

Response:

We have corrected this in the manuscript.

page 6: "(1) data publishing through international trusted data repositories, such as INSDC [7], GBIF [9], and others, a.. "

-> drop "and others" to simplify the sentence.

Response:

We have corrected this in the manuscript.

page 7: "Furthermore, it ensures a scientific record, crediting and acknowledgement for the data creators and scientists in the form of citable scholarly articles."

rephrase or complete, eg: "Furthermore, it ensures that a citable scholarly scientific record, crediting and acknowledging the daat creators.... is created'.

Response:

We have rephrased this sentence to make it more clear while preserving the meaning. The new sentence is: "Furthermore, it creates a citable scientific record, enabling the crediting and acknowledgement of the data creators and researchers."

page 7: "As more and more researchers want to deposit and share their datasets, standards, infrastructures, and workflows become central to delivering FAIR data.

rephrase, eg: "As more and more researchers want to deposit and share their datasets, new tools and new approaches are needed to deliver FAIR data.

Response:

	We have rephrased this sentence according to your suggestion.<br><br>page 8: "to describe a prototyped workflow"<br>-> s/prototyped/prototype/<br><br>Response:<br><br>We have corrected this sentence in the manuscript.<br><br>page 9: "We created a template, "<br>-> "We created a dedicated data article template, "<br><br>Response:<br><br>We have corrected this sentence in the manuscript to “We created a dedicated data paper template”...<br><br>page 23, section 1: "is an meticulous approach to", s/an/a/<br><br>Response:<br><br>We have corrected this mistake in the manuscript.<br><br>page 24, section 2: "Throughout our testing phase", add a comma after 'phase'<br><br>Response:<br><br>We have corrected this mistake in the manuscript.
**Additional Information:**	
**Question**	**Response**
Are you submitting this manuscript to a special series or article collection?	No
**Experimental design and statistics**<br><br>Full details of the experimental design and statistical methods used should be given in the Methods section, as detailed in our Minimum Standards Reporting Checklist. Information essential to interpreting the data presented should be made available in the figure legends.<br><br>Have you included all the information requested in your manuscript?	Yes
**Resources**<br><br>A description of all resources used, including antibodies, cell lines, animals and software tools, with enough information to allow them to be uniquely	Yes

identified, should be included in the Methods section. Authors are strongly encouraged to cite Research Resource Identifiers (RRIDs) for antibodies, model organisms and tools, where possible.<br><br>Have you included the information requested as detailed in our Minimum Standards Reporting Checklist?	
**Availability of data and materials**<br><br>All datasets and code on which the conclusions of the paper rely must be either included in your submission or deposited in publicly available repositories (where available and ethically appropriate), referencing such data using a unique identifier in the references and in the "Availability of Data and Materials" section of your manuscript.<br><br>Have you have met the above requirement as detailed in our Minimum Standards Reporting Checklist?	Yes

Powered by Editorial Manager® and ProduXion Manager® from Aries Systems Corporation

Manuscript

Click here to access/download;Manuscript;Dimitrova_et_al_Omics_Data_Pa

A streamlined workflow for conversion, peer review and publication of genomics metadata as Omics Data Papers

Mariya Dimitrova[1*], Raïssa Meyer[2], Pier Luigi Buttigieg[3], Teodor Georgiev[4], Georgi Zhelezov[5], Seyhan Demirov[6], Vincent Smith[7], Lyubomir Penev[8]

[1] Pensoft Publishers, Prof. Georgi Zlatarski Street 12, 1700 Sofia, Bulgaria;
Institute of Information and Communication Technologies, Bulgarian Academy of Sciences, Acad. G. Bonchev St., Block 25A, 1113 Sofia, Bulgaria
Correspondence address: Pensoft Publishers, Prof. Georgi Zlatarski Street 12, 1700 Sofia, Bulgaria
Email: m.dimitrova@pensoft.net
https://orcid.org/0000-0002-8083-6048
*Corresponding author

[2] Alfred-Wegener-Institut, Helmholtz-Zentrum für Polar- und Meeresforschung, Bremerhaven, Germany
Correspondence address: Alfred-Wegener-Institut, Helmholtz-Zentrum für Polar- und Meeresforschung, Bremerhaven, Germany
Email: raissa.meyer@awi.de
https://orcid.org/0000-0002-2996-719X

[3] Alfred-Wegener-Institut, Helmholtz-Zentrum für Polar- und Meeresforschung, Bremerhaven, Germany
Correspondence address: Alfred-Wegener-Institut, Helmholtz-Zentrum für Polar- und Meeresforschung, Bremerhaven, Germany
Email: pier.buttigieg@awi.de
https://orcid.org/0000-0002-4366-3088

[4] Pensoft Publishers, Prof. Georgi Zlatarski Street 12, 1700 Sofia, Bulgaria
Correspondence address: Pensoft Publishers, Prof. Georgi Zlatarski Street 12, 1700 Sofia, Bulgaria
Email: t.georgiev@pensoft.net
https://orcid.org/0000-0001-8558-6845

[5] Pensoft Publishers, Prof. Georgi Zlatarski Street 12, 1700 Sofia, Bulgaria
Correspondence address: Pensoft Publishers, Prof. Georgi Zlatarski Street 12, 1700 Sofia, Bulgaria
Email: g.zhelezov@pensoft.net

[6] Pensoft Publishers, Prof. Georgi Zlatarski Street 12, 1700 Sofia, Bulgaria
Correspondence address: Pensoft Publishers, Prof. Georgi Zlatarski Street 12, 1700 Sofia, Bulgaria
Email: programmer@pensoft.net

[7] The Natural History Museum, London, United Kingdom
Correspondence address: The Natural History Museum, London, United Kingdom
Email: vince@vsmith.info

https://orcid.org/0000-0001-5297-7452

[8] Pensoft Publishers, Prof. Georgi Zlatarski Street 12, 1700 Sofia, Bulgaria;
Institute of Biodiversity and Ecosystem Research, Bulgarian Academy of Sciences, 2 Gagarin Street, 1113 Sofia, Bulgaria
Correspondence address: Pensoft Publishers, Prof. Georgi Zlatarski Street 12, 1700 Sofia, Bulgaria
Email: l.penev@pensoft.net
https://orcid.org/0000-0002-2186-5033

Abstract

Background

Data papers have emerged as a powerful instrument for open data publishing, obtaining credit, and establishing priority for datasets generated in scientific experiments. Academic publishing improves data and metadata quality through peer-review and increases the impact of datasets by enhancing their visibility, accessibility, and re-usability.

Objective

We aimed to establish a new type of article structure and template for omics studies: the omics data paper. To improve data interoperability and further incentivise researchers to publish well-described datasets, we created a prototype workflow for streamlined import of genomics metadata from the European Nucleotide Archive directly into a data paper manuscript.

Methods

An omics data paper template was designed by defining key article sections which encourage the description of omics datasets and methodologies. A metadata import workflow, based on REpresentational State Transfer services and Xpath, was prototyped to extract information from the European Nucleotide Archive, ArrayExpress and BioSamples databases.

Findings

The template and workflow for automatic import of standard-compliant metadata into an omics data paper manuscript provide a mechanism for enhancing existing metadata through publishing.

Conclusion

The omics data paper structure and workflow for import of genomics metadata help to bring genomic and other omics datasets into the spotlight. Promoting enhanced metadata descriptions and enforcing manuscript peer review and data auditing of the underlying datasets brings additional quality to datasets. We hope that streamlined metadata re-use for scholarly publishing encourages authors to create enhanced metadata descriptions in the form of data papers to improve both the quality of their metadata and its findability and accessibility.

Keywords

data, data paper, omics, genomics, metadata, workflow, standards, FAIR principles, MIxS, MINSEQE

1. Introduction

The term “omics” refers to the study of biological systems through the examination of different elements of the molecular basis of life. Many fields of molecular biology, thus, derive their name from the suffix “omics” (e.g. genomics, transcriptomics, metabolomics). The genome is examined through the analysis of gene (DNA) sequences, the transcriptome is the collection of all mRNA molecules in an organism, and the metabolome is the collection of all metabolites and intermediate substrates participating in the metabolic pathways. Omic studies are generating large quantities of deeply minable data with increasing scale and complexity [1, 2]. Further, omics technologies and approaches have revolutionised biodiversity science [3, 4, 5].

Independently from the recent advances in omics technologies and data generation, however, the published omics biodiversity data and its accompanying, standardised metadata, are still neither harmonised nor interoperable [6]. Existing infrastructures in omics data science focus on the sequence or molecular data generated from omics studies. Since 1988, the databases of the International Nucleotide Sequence Database Collaboration (INSDC) [7, 8, 9] have provided a trusted archive for these data. In parallel, major infrastructures to handle higher-order biodiversity data (e.g. occurrences linked to taxa, specimen records) have emerged and include the Global Biodiversity Information Facility (GBIF) [10], the Integrated Digitized Biocollections (iDigBio) [11], the Distributed System of Scientific Collections (DiSSCo) [12], the Ocean Biogeographic Information System (OBIS) [13], the Global Genome Biodiversity Network (GGBN) [14], DataONE [15] and others. Some of these infrastructures support data repositories which follow community-accepted metadata standards. GBIF uses the Ecological Metadata Language (EML) standard for describing ecological datasets in XML files [16], whereas biodiversity data is recorded by following the Darwin Core Standard (DwC) [17, 18]. Likewise, the GGBN have developed their own GGBN Data Standard, which interoperates with DwC and the Access to Biological Collections Data (ABCD) schema for primary biodiversity data [19, 20, 21]. The INSDC cooperates with community standards initiatives such as the Genomic Standards Consortium (GSC) to implement their Minimum Information about any (x) Sequence (MIxS) checklists for genomic, metagenomic and environmental metadata descriptors, and with the Global Microbial Identifier (GMI) group for pathogen sequence metadata [22, 23]. MIxS consists of three checklists, each containing several packages for the description of various environments where genomic material could be sampled from [23]. Other international data repositories

such as EBI EMBL's ArrayExpress [24] and the BioSamples [25] database implement standards such as Minimum Information about a high-throughput nucleotide SEQuencing Experiment (MINSEQE) and Minimum Information About a Microarray Experiment (MIAME) and various MIxS environmental checklists [26]. Databases such as the Genomic Observatories Metadatabase (GeOMe) [27] offer integrative solutions for the data management of genomic, geographical and ecological metadata by providing mechanisms to create standard-compliant templates tailored to specific use cases and linking metadata to dataset records via stable identifiers [28].

A more comprehensive approach towards omics metadata mobilisation is undertaken by the ISA Commons community [29], which uses the "extensible, cross-domain format" ISA-Tab for organising metadata [30]. This format focuses on three major components of any scientific research: "Investigation", "Study" and "Assay" to help structure the underlying study and assay specific metadata records [30]. Software for creating and validating ISA-Tab files has also been developed as part of the ISA Tools framework [30]. This framework aims to complement existing omics standards to improve the description of research outputs in the field of omics. Several omics data repositories, such as EMBL-EBL's Metabolights [31] and *GigaScience*'s GigaDB [32], have utilised the ISA model and serialisations to integrate experimental metadata.

There are different ways scientists can publish their data in a FAIR (Findable, Accessible, Interoperable and Re-usable) [33, 34] manner, however, all can be attributed to two main routes: (1) data publishing through international trusted data repositories, such as INSDC [7] and GBIF [10], and (2) scholarly data publishing in the form of data papers or as data underpinning a research article [35, 36, 37, 38, 39, 40].

While the first route focuses on data aggregation, standardisation and re-use, the second one augments the quality and reusability of data and metadata through peer reviewing and data auditing in the scholarly publishing process. Scholarly data publishing provides an opportunity to enhance the original metadata in the data paper narrative and to link it to the original dataset via stable identifiers, thus improving the reproducibility and findability of the data [35]. Furthermore, it creates a citable scientific record, enabling the crediting and acknowledgement of the data creators and researchers. Academic publishing involves dissemination of research through additional channels, such as journal distribution networks, and creates increased opportunities for open science collaboration [35].

While standards and infrastructures are crucial to the advancement of data sharing and reuse within the field of omics, we argue that incentivising authors to publish their data in the form of peer reviewed journal articles (data papers) creates the driving force towards a truly FAIR data world (Fig. 1).

Fig. 1. The different layers of FAIRness of data and metadata. Describing data and metadata in a data paper publication helps to enhance their FAIRness through provision of better visibility and accessibility.

As more and more researchers want to deposit and share their datasets, new tools and new approaches are needed to deliver FAIR data. Following the example set by Chavan and Penev [35], who introduced data papers in biodiversity science, we have established a concept for an omics data paper - a type of scholarly paper in which

data, generated in genomic or other omic experiments, is described with extended and peer reviewed metadata, and linked to the corresponding dataset(s) deposited in an INSDC database or other archive. To further incentivise authors to publish omics data papers and to demonstrate the importance of high-quality metadata, we propose a prototype of a streamlined workflow for conversion of European Nucleotide Archive (ENA) genomic metadata directly into a data paper manuscript. We build upon previous work by Pensoft, namely workflows for automatic import of EML metadata from GBIF, DataONE and LTER [41] as well as Food Safety Knowledge Markup Language (FSK-ML) metadata [42] into data paper manuscripts.

The aim of the present paper is to conceptualise the omics data paper, to create a specific article template for it, and to describe a prototype workflow for automated import of genomic metadata into an omics data paper manuscript. This workflow also accommodates the peer review and publication processes associated with the manuscript.

2. Methods

Approach

We took the following steps to approach the goal of establishing an omics data paper template and workflow:

1. Identify the high-level needs of the omics communities to better describe their datasets
2. Review existing standards, infrastructures [43] and datasets, as well as the existing data paper formats for describing (gen)omic data [44, 45, 46].

3. Synthesise the technical solutions and incorporate further functional needs to create the structure of the new type of data paper.

We created a dedicated data paper template, defining article sections and subsections to map the article narrative to metadata associated with the dataset(s) described in an omics data paper.

Workflow for extracting relevant metadata from ENA XML files

We developed a workflow for automatic import of metadata into omics data paper manuscripts based on ENA's metadata structure, as well as the ArrayExpress [24] and BioSamples [25] databases. The workflow uses REST API requests and Xpath to retrieve segments of information from XML files from ENA, ArrayExpress and BioSamples [47]. It then imports them into our proposed data paper manuscript structure, filling in the relevant subsections.

For demonstration, testing and reproducibility purposes, this workflow was implemented in a R Shiny app [48, 49, 50] which visualises metadata extracted from ENA inside the relevant sections of the proposed manuscript template within the application interface. The application also enables import of metadata into a valid JATS XML [51] document. Download of the HTML and XML versions of the metadata, as well as supporting supplementary material is also enabled via reactive buttons in the user interface.

The R Shiny app was transformed into an installable R package using the golem framework [52], ensuring that it can be installed and run on any computer with R and RStudio. The package can be installed and run with just three R commands, which

are documented in its Github repository along with all code, released under the Apache 2.0 license [53]. The R version at the time of developing the R Shiny app was R version 4.0.0 (2020-04-24) (Arbor Day) [50].

In addition, the R Shiny app can be run without installation as an interactive web app [54] deployed in an RStudio cloud environment [56] and hosted on a Shinyapps.io server [48, 49]. The code behind the interactive web app is openly available on Github [55] under the Apache 2.0 license.

Integration of metadata extraction workflow with the ARPHA Writing Tool

After testing the metadata extraction and import workflow in the R shiny app, it was realised as a production-grade workflow integrated via Web service with the Pensoft's ARPHA Writing Tool (AWT) [40]. The AWT is a web platform for collaborative authoring, reviewing and publishing of manuscripts, developed and used by Pensoft in their publication process. It supports the creation of manuscripts by manual entry of text into different templates corresponding to separate article types, such as "Research Article", "Software Description", "Data Paper", etc. In addition, there are existing implemented workflows for import of metadata from files or web resources into the templates of some article types (e.g. "Data Paper" or "FSKX (Food Safety Knowledge) Paper") [40, 41]. After acceptance, the data papers consisting of peer-reviewed, corrected and extended metadata descriptors, are published under the Creative Commons CC-BY 4.0 license which allows free and unlimited distribution and re-use given that the original source is credited. The datasets described in the data paper are made available under a license determined by the authors which is specified in a dedicated "Usage rights" section of the data paper. Thus, we address one of the

key aspects of the FAIR principles (R1.1) specifying that "(meta)data are released with a clear and accessible data usage license" to achieve data reusability [33].

Similarly, we established a new publication type, "OMICS Data Paper", and a manuscript template for it [see Additional file 1], following the proposed data paper structure. Essential sections of the omics data paper template were made mandatory in AWT such as the "Methods" section and the "Data resources" section. This means that the system requires the authors to fill them in before they can submit the manuscript for review.

We then replicated the genomic metadata import workflow from the R Shiny app inside the ARPHA Writing Tool. The workflow was designed to automatically populate some of the fields from the omics data paper manuscript template. It must be noted that not all fields from the template would be automatically filled in with metadata records by the workflow because ENA metadata records only cover a limited amount of information. For instance, sections such as "Environmental profile" and "Societal value" would not be populated and the users would have to manually fill them in with information, if they wish to keep these sections in the data paper.

An important component of the design and implementation of the omics data papers is the BioSamples Supplementary Table. ENA metadata records that contain links to associated BioSamples metadata (MIxS checklists) [23, 25] are retrieved by the automatic import workflow, and are transformed into a narrow format table, which will be attached to the manuscript as a comma-separated value (CSV) file. We restrict editing of Supplementary Tables imported from BioSamples to prevent metadata loss and tampering. Authors can only change the MIxS checklists related to their

manuscript if they re-upload them to the long-term, trusted source repository: BioSamples. Synchronisation with BioSamples from the manuscript in the ARPHA Writing Tool is enabled through a button labelled “Re-import from BioSamples”.

3. Findings

Structure of the OMICS data paper

The omics data paper describes datasets generated in omics research. The described dataset is at the core of the data paper, but the methodology required to obtain it is just as valuable as the data itself. To guide authors in the authoring process and to better inform the readers about the contents of the proposed data paper, we designed a detailed manuscript template. Table 1 outlines each section and associated subsections of the template to be used either for manual population in the AWT, or to match the metadata records extracted by the workflow to populate certain sections and subsections of the template. Many data paper sections do not have ENA metadata fields associated with them and the authors are encouraged to fill in their contents in the data paper manuscript as well as to update the original ENA record accordingly if possible.

**Article section**	**Purpose**	**ENA metadata source field**
**Abstract**	Summary of the value of the study, the experimental design and the dataset itself.	**Study/Project XML:** //abstract

Introduction<br>- Value of the dataset<br>- Scientific value<br>- Societal value	Outline of the reason for the study. Authors should put into perspective its value for the scientific and broader communities. Often sequencing studies are part of large-scale genome sequencing projects and this article section allows authors to explain their role in them.	*Written by the authors*
Methods<br>- **Sampling**<br>- Environmental profile<br>- Geographic range<br>- Technologies used<br>- Sample processing<br>- **Technologies used**<br>- Data processing	This section is split into 3 major parts to describe how the physical material was collected, processed and transformed into a dataset.<br>The “Sampling” section allows authors to outline the environmental and geographic characteristics of the locations where their material was collected. Sampling metadata imported from ENA fills in the “Sampling” section but the “Environmental profile” and “Geographic range” subsections remain to be filled in by the author manually. Authors are encouraged to share as much detail as they can (e.g. geographic coordinates, habitats, seasonal information, etc.). The sampling methods should be described in the “Technologies used” subsection.<br>“Sample processing” should explain the laboratory procedures involved in the transition of the physical sample into its digital footprint. Finally,	**ArrayExpress XML> Protocol XMLs:**<br>protocol/type<br>protocol/text<br>protocol/hardware<br>protocol/software<br><br>And<br><br>**Experiment XMLs:**<br>//EXPERIMENT/DESIGN/LIBRARY_DESCRIPTOR/LIBRARY_STRATEGY<br><br>And<br><br>**Experiment XMLs:**<br>//EXPERIMENT/PLATFORM (-> Sample processing/Technologies used)<br><br>And<br><br>**Sample XMLs:**<br><br>//SAMPLE/DESCRIPTION<br>//SAMPLE/SAMPLE_ATTRIBUTES/SAMPLE_ATTRIBUTE

	the “Data Processing” subsection should mention the steps taken to transform the raw dataset into the one which was published (e.g. normalisation steps). None of the subsections are compulsory and the authors can write the Methods in a form outside these topics but our template provides a detailed best practices structure to follow.	
Biodiversity profile<br>- Target<br>- Taxonomic range<br>- Functional range<br>- Traits	This section describes the experimental design of the study. The target refers to the molecular target being studied (i.e. DNA, RNA, protein). The taxonomic range refers to the taxonomy of the studied organism(s) or the taxonomic composition of a metagenomic sample. The authors are encouraged to use a common taxonomy but they can also provide their own during the authoring process in AWT. Authors can specify a particular range of biological functions which was the subject of their study (e.g. metabolic functions), as well as specific traits (e.g. pathogenicity) if relevant to the study.	*Written by the authors*
**Data resources**	This is the section which contains a link to the dataset(s) (preferably to its permanent resolvable identifier, such as a DOI), as well as any accession numbers and data formats.	**Study/Project XML:** //XREF_LINK/ID[../DB='ENA-FASTQ-FILES']

Data statistics	Quantitative and qualitative description of the dataset. (e.g. read depth, coverage, base ratios). This section helps readers to quickly evaluate the dataset by gauging some of its characteristics without having analysed the dataset themselves. Some of the data statistics can be represented as charts and/or short tables.	*Written by the authors*
Caveats and limitations	A section to discuss what could be improved in the experiment, what future steps could be taken and what to consider when re-using the published data.	*Written by the authors*
Usage rights	Rights and licenses to use the data. The data paper is open access by default Authors can read more about Pensoft's recommended data publishing licenses in [39]	*Written by the authors*
**Supplementary table**	Contains imported MIxS checklists for the imported BioSamples. The checklists are in long format. The table can be downloaded as a separate comma-separated value (CSV) file after publication.	**Sample XMLs:** //SAMPLE/IDENTIFIERS/EXTERNAL_ID[@namespace="BioSample"]<br><br>And<br><br>**BioSample XMLs:** //Property[@class]

Table 1. OMICS data paper sections, their purpose and ENA metadata fields from which they are populated, if such fields exist. The names of manuscript sections which could be automatically populated by the workflow are marked in bold in the first column. Values in the third column refer to the fields in ENA's XML files which contain the information used to automatically fill in the relevant section of the template. We

have pointed to the type of XML (marked in bold) as well as the Xpath used to extract the information.

The template focuses on the value of the data, the methods used to generate it and the qualitative and quantitative characteristics of the dataset. We have included a section to describe the biological entities which are the focus of the research: “Biodiversity profile”. In addition to filing in the relevant subsections of this section, authors can attach a supplementary EML file or an Appendix table [57] to describe the different dimensions of the research target. Such Appendix tables can be used to record and link taxonomic, genomic, ecology, image and other types of data using community agreed vocabularies and ontologies. A spreadsheet template and instructions have been published as part of the Author’s Guidelines of the *Biodiversity Data Journal* (BDJ) [58].

Genomics metadata extraction workflow

Omics data papers can be created via two separate routes: 1) manually, by filling in all sections from the omics data paper template relevant to the research experiment inside the ARPHA Writing Tool and 2) semi-automatically, by using the genomics metadata extraction workflow with ENA metadata records and later manually enhancing the extracted metadata by filling in missing information inside the ARPHA Writing Tool. Here we outline the second route.

Metadata describing the datasets was utilised to facilitate creation and authoring of the data paper manuscript. By following ENA’s metadata model [43], including its links to the ArrayExpress [24] and BioSamples [25] databases, we designed a workflow

which orchestrates the extraction of relevant metadata from the various ENA XML files (Fig. 2). The Study XML and the Project XML are the starting points in the proposed workflow as they integrate all other types of data and metadata available in ENA for a given scientific study. Each metadata object in the ENA metadata model is associated with a unique identifier, which can be used to retrieve its corresponding XML file via the ENA API [43].

Fig. 2 Metadata extraction workflow from ENA, ArrayExpress and BioSamples

As outlined in our proposed workflow (Fig. 2), the Study or Project accession number is used to obtain a XML file which contains the accession numbers for all associated Experiment and Sample metadata objects, and in some cases ArrayExpress and BioSamples metadata objects.

ArrayExpress is a database storing data and metadata from functional genomic microarray or sequencing experiments [24]. ArrayExpress' own submission platform Annotare and curators ensure that metadata from all sequencing experiments follow the Minimum Information About a Sequencing Experiment (MINSEQE) standard [24, 26].

Raw data from sequencing experiments submitted to ArrayExpress are also automatically deposited in ENA [47] as part of a Study metadata object, linked to Experiment and Sample objects [59]. Provenance of metadata imported from ArrayExpress can be established through a unique ArrayExpress accession number

in the ENA Study XML. We integrated the extraction of curated, MINSEQE compliant metadata from ArrayExpress into the workflow, thus enhancing manuscripts with additional metadata about experimental design and methodologies.

Another database within EMBL-EBI's infrastructure is BioSamples, a database which "stores and supplies descriptions and metadata about biological samples" [25]. Metadata descriptors in BioSamples records follow the MIxS standard [26]. Depending on the type of sample, submission to BioSamples requires different MIxS checklists to be filled in, after which they are publicly available in the form of XML files [25]. Unique identifiers link Sample XMLs from ENA with their associated BioSamples XML records. Thus, we are able to extract BioSamples information for any samples from a given ENA Study or Project. BioSamples records are imported into a table which is attached to the manuscript as a Supplementary CSV file. This supplementary table is a mandatory component of a manuscript, when accompanying BioSamples records are available, and cannot be removed by the authors. In cases when the authors spot a mistake in their submitted metadata, they are encouraged to change it within the BioSamples database. Upload of standalone BioSamples MIxS checklists is not permitted in the ARPHA Writing Tool, so that authors perform their corrections in the original metadata repository. After that, they can automatically retrieve them from BioSamples and import them into the manuscript with the click of a button. Thus, we promote the reuse and interoperability of MIxS compliant metadata sourced from BioSamples.

We implemented the template and workflow into Pensoft's ARPHA Writing Tool [40], enabling import of the extracted ENA metadata records into the omics data paper

template (Table 1). Fig. 3 shows a diagram demonstrating the import functionality from the perspective of the user.

Fig. 3. Automatic metadata import from ENA, ArrayExpress and BioSamples, facilitates the creation of omics data paper manuscripts inside the ARPHA Writing Tool.

R shiny app - deployment and reproducibility

The template and workflow were first prototyped in a R shiny app [54], the code for which is open source and available on Github under Apache 2.0 license [55, 56], as outlined in the Methodology section of this paper. The R shiny app is a web application emulating the functionality of the metadata import workflow in the ARPHA Writing Tool. The application runs in a virtual R environment [48, 56] and is deployed and hosted on the web via Shinyapps.io [49], configured to allow up to 50 concurrent connections. The interface of the application features a text field for input of ENA Study or Project ID and an 'Convert' button controlling the import of metadata and conversion to manuscript. Three buttons to download the outputs appear after the 'Convert' button is clicked. The generated manuscript narrative, along with a data frame containing the BioSamples MIxS checklist, are visualised in the R shiny app interface. The narrative can be downloaded as a HTML file by clicking the 'Download HTML' button, whereas the BioSamples checklist can be downloaded as a CSV file by clicking the 'Download Supplementary Material' button. This CSV file is identical to the one generated as a supplementary file by the ARPHA Writing Tool.

The R shiny app has one additional functionality, which is not present in the workflow implemented in the ARPHA Writing Tool: it transforms the imported metadata into a

Journal Article Tag Suite (JATS) XML file [51], which can be downloaded by clicking the 'Download XML' button. We validated the XML against the latest JATS DTD version with the JATS4R validator [60]. The JATS XML is structured according to the Pensoft omics data paper template so that most article section nodes are defined with the sec tag and an attribute sec-type is used to define the exact section name (e.g. the Methods section is marked in the XML as <sec sec-type="Methods">). A basic "skeleton" file of the JATS XML file is available in the Github repository containing the code of the interactive web app [55].

Despite being tailored to the Pensoft omics data paper template, JATS XML files generated via the R shiny app can be used by other publishers or individuals to generate their own omics data paper manuscripts. Together with ENA's documentation about programmatic access to its resources [47], the codebase enables reproducibility of our workflow and creates the potential for it to be deployed by other journals or publishers.

4. Discussion

The data and metadata publishing landscape

The concept of data papers is not new; in fact, they have been in existence for more than two decades already. One of the first journals to implement this concept was Ecological Society of America's Ecological Archives [61, 62]. In 2011, Chavan and Penev envisioned metadata as a resource for authoring data papers for primary biodiversity data and identified a lack of clear guidelines and good practices for authoring metadata (the "how") and the incentives for authors to do so (the "why") [35]. They proposed data papers as a "mechanism to incentivise data publishing in

biodiversity science" and introduced them to the biodiversity community through Pensoft's journals. To further simplify data paper authoring, Pensoft pioneered an integrated workflow for automatic metadata-to-manuscript conversion of primary biodiversity datasets published through GBIF's Integrated Publishing Toolkit (IPT) [35, 38, 40, 41, 63].

This streamlined metadata conversion workflow was first introduced in several of Pensoft's biodiversity journals and then in journals by other publishers, such as Nature's *Scientific Data*, *PLOS ONE*, *BMC Ecology* and many others [64]. Since 2011, nearly 300 data papers have been published in Pensoft's journals and there is a steady uptake of this type of publication not only among Pensoft's journals but among journals of other publishers too [65]. Data papers are no longer an abstract idea but have already been practically implemented in multiple journals in different disciplines.

Since 2011, Pensoft has developed other integrative ways to streamline metadata authoring and data paper publication by integrating different workflows into its collaborative online authoring tool, the ARPHA Writing Tool (AWT) and associated *Biodiversity Data Journal* [66]. For instance, metadata files following the GBIF EML profile used in the IPT can be directly converted and imported into manuscripts in AWT "at the click of a button", then edited in the tool and submitted to the *Biodiversity Data Journal* [63, 67]. This workflow closely resembles the workflow described in this paper but it is focused on ecological data. The EML workflow accepts a single specimen record identifier and imports information about that record from several infrastructures (GBIF, Barcode of Life Data Systems (BOLD), iDigBio, or PlutoF) into manuscripts [67]. It also enables conversion of an EML-formatted file into a biodiversity data paper

[67], a functionality not covered by the present workflow, which only performs API requests.

Generation of extended metadata descriptors has been the focus of other tools, such as the Metadata Shiny Automated Resources and Knowledge (MetaShARK) [68] and Datascriptor [69], which is still under development. MetaShARK aims to facilitate assembly of ecology metadata by providing a user-friendly workflow for metadata packaging [68]. Unlike the workflow described here, it is more focused on primary metadata generation than metadata sharing and reuse [68]. Our workflow uses already generated metadata and provides a template for their extension to create an extended metadata description converted to narrative. Datascriptor is more closely related to our workflow because it aims to transform metadata, generated by following community standards, into a data article [69]. To do so, the developers have envisioned the generation of a JATS XML [69], which is what we have implemented in our R shiny app demonstrating the workflow for import of metadata into omics data paper manuscript.

Data papers for the field of omics: rationale and benefits

Generation of omic data and metadata is one of the very first outputs of the research cycle, but not all of this is shared via research publications. Even when these data are published, the focus is usually on the interpretation of the data, rather than metadata quality or the FAIR properties of the dataset. Deposition of raw omic data, such as sequencing data, mass spectrometry (MS) proteomic data and RNA-sequencing data, into centralised databases has become a routine practice for studies involving omic experiments [70]. ENA provides the necessary infrastructure to share sequencing data

in a structured format and enables machine-readability and interoperability through the use of identifiers, consistent schema models and APIs [43]. Describing that data, including its limitations and opportunities, inside a human readable narrative will further improve its interpretation and reusability, and increase its impact. With our proposed omics data paper and the automatic import prototype workflow, we encapsulate all metadata about a study into a single piece of narrative, thus completing the scientific process.

Authoring omics data papers, despite being aided by the automated workflow, requires additional effort and time largely because ENA records do not contain all metadata needed to assemble a thorough genomics study description. As the quality, breadth, and depth of metadata records in the sourced repositories improves, that additional effort will decrease. The prototype workflow merely demonstrates the possibility of interoperable metadata sharing and integration inside the publishing process. Here we outline some of the benefits which make the process of creating such manuscripts worthwhile, as well as on how data interoperability contributes to the FAIR data and metadata publishing landscape.

1. *Omics data papers and underlying datasets undergo peer-review and data auditing*

   Prior to peer-review of submitted omics data paper manuscripts, all underlying datasets go through mandatory data auditing, cleaning and quality checks to assure that they meet the journal standards for publication [71]. This is done by a data auditor, whose role is to technically evaluate the submitted datasets for

compliance to a data quality checklist [71] and to provide authors with a detailed report, including recommendations for improving the dataset. Only after the authors change the dataset according to the recommendations, it can be approved for peer-review. Peer reviewers review not only the narrative but also the datasets according to the Data Review Guidelines [73]. The introduction of data scientists into the publishing process ensures to an extent that submitted data and metadata are FAIR and consistent. This double checking - first of the datasets by the data auditors and then of the whole manuscript by the reviewers - is a meticulous approach to enhancing the quality of datasets and to the best of our knowledge has not been adopted by any other publisher so far.

2. *Publication of data papers improves metadata quality*

Authoring metadata is a necessary step to publish omics data into an open repository; however, there is considerable variability when it comes to the quality of the published metadata [6]. The workflow allows metadata authors, metadata standard creators and data repository managers to evaluate the quality of metadata files deposited to INSDC databases. Throughout our testing phase we came across many datasets with missing or incorrectly formatted metadata fields. A recent observation by members of the Genomics Standards Consortium found that missing or incomplete metadata records from SARS-CoV-2 genomic and metagenomic studies are of frequent occurrence in INSDC databases and other repositories: such deficits of high-quality, community-standard metadata, have become apparent during the COVID-19 healthcare crisis as global scientific efforts have been directed at generating and analysing

data related to the novel coronavirus and data sharing and re-use have become crucial [6]. Currently, there is no workflow in place to feed information about such missing or incorrect records back to the INSDC databases but we are optimistic about future integrative efforts that would help to streamline metadata between the INSDC repositories and other repositories or workflows. For example, such a feedback mechanism is one of the key deliverables of the EU funded project BiCIKL starting in May 2021.

Some data repositories enforce in-house data or metadata curation practices. Curation of metadata, currently implemented by ArrayExpress via their Annotare tool [74, 75], is an adequate method for high-quality metadata publishing, based on standards. Most important, however, is that metadata authors learn to adopt and correctly use the existing standards in the process of describing their data. By directly observing the role of their metadata in creating the manuscript, they are made aware of its value and should be incentivised to improve the quality and quantity of the metadata they provide. After importing metadata into their omics data paper manuscript, authors would need to manually correct and fill in the missing information, which defies the main purpose of the workflow: to make the data better described through extended and detailed metadata in the form of peer-reviewed, widely accessible and citable data papers.

3. *High-quality metadata enables data-driven discovery*

Metadata which follows community accepted standards is vital for data-driven discoveries as it provides the necessary context to characterise the dataset it describes. Omics data papers not only improve the quality of the metadata but also constitute an enhanced metadata record themselves.

After publication all data papers are publicly available also as JATS XML and submitted as such for archiving and display to PubMed Central [39], in addition to the traditional PDF and HTML formats. Therefore, all identifiers and links to external databases are marked up in the XML version of the article and can then be indexed by Web crawlers, including Pensoft's own RDFization scripts which transform XML articles to Resource Description Framework (RDF) to ensure machine readability of the text [76]. Thus, the omics dataset metadata is indexed in several locations and becomes more findable and accessible.

The improved visibility of the omics datasets can open new possibilities for re-use of the datasets. For instance, in pharmaceutical science, old compounds are commonly researched as part of the development of new drugs because they could harbour unexplored biological activities [77]. By giving further visibility to omic datasets through their publication in an omics data paper, indexed in journal-focused databases and search engines, and by enhancing metadata through publication, we stimulate scientific research and data-driven discovery.

4. *Data papers help to establish priority*

Publishing data papers at early stages of the research process can provide an important benefit for authors: the opportunity to get the first scientific record for their effort in assembling a dataset and obtain feedback from the research community. It is well known that many authors are hesitant to publish datasets which they have not yet analysed or used for supporting any research findings for fear of someone else using the data and getting ‘scooped’. By publishing a data paper, the authors are guaranteed that the described data can be re-used in accordance with the Open Science principles, following all community accepted ethical norms for citation, priority and generating new knowledge through joint publications based on shared data.

5. *Publishing omics data papers is a way to obtain credit for one’s work*

Science crediting further incentivises researchers to publish omics data papers because their work impact can be measured in a way familiar to authors of traditional research papers, adding to their researcher impact metrics. In addition, the data managers and scientists who generate the data are not always among the authors of traditional research articles, which focus on the data analysis and outcomes. Thus, data paper publishing can be a way for all actors involved in the process of gathering, curating and managing the data - be they early stage researchers, technicians or data scientists - to obtain credit for their valuable work.

Limitations and future outlooks

The automated workflow prototype for importing omics metadata into data paper manuscripts currently works only with ENA metadata records. While INSDC metadata is exchanged across all three databases in the consortium (ENA, GenBank and DDBJ) [7], it would be beneficial if users could import metadata from any of the three data repositories via their associated identifier. The reason for the current limitation is the requirement for additional integrations, produced by the variation of APIs and the differing metadata schemas even between ENA, GenBank and DDBJ repositories which hold identical data and are synchronised. We decided to integrate the prototype workflow with ENA as the first showcase of this novel method of creation of data paper manuscripts because of the more straightforward links between ENA, BioSamples and ArrayExpress compared to GenBank or DDBJ.

Currently, the streamlined metadata import workflow for the omics data paper is focused mostly on genomic data. In the future, we plan to expand the workflow to include other repositories and data types, such as metagenomics data and operational taxonomic units (OTU) tables. This addition will integrate new data science solutions for efficiently and interoperably exchanging and storing sparse and high dimensional contingency tables along with their associated sample and taxonomic metadata (e.g. the BIOM format [78]). Thus, we support the development away from the fragmentation of data and towards a single quantum of information to exchange, containing interoperable, accessible, and transparent information. Making use of this advancement, future workflows for omics data paper creation may also support BIOM files for data provision, as outlined in an unpublished dissertation by Raïssa Meyer (2020).

Integrations between existing infrastructures and data-driven initiatives are key to the FAIRness of data and metadata. The streamlined workflow for import of metadata from ENA, ArrayExpress and BioSample is another step in this direction. However, to make metadata truly FAIR, there should be a two-way link between the original data and metadata repository (e.g. ENA) and the enhanced metadata record (e.g. the omics data paper).

5. Conclusions

In conclusion, the new omics data paper, implemented in Pensoft's publishing process provides a mechanism for incentivising omics data sharing and reuse through scholarly publishing. In addition, the workflow for import of metadata into manuscripts encourages and incentivises authors to enhance data quality and completeness. The workflow also demonstrates the importance of linking data from different infrastructures using stable identifiers and thus sets an example for future integrations with other metadata and data repositories.

Availability of supporting source code and requirements

- Project name: Omics Data Paper Generator
- Project home page: https://github.com/pensoft/omicsdatapaper
- Operating system(s): Platform independent
- Programming language: R
- Other requirements: R version 4.0.0 (2020-04-24) (Arbor Day), R Studio
- License: Apache 2.0
- RRID: SCR_019809

- biotoolsID: biotools:omics-data-paper-shinyapp-golem

Data Availability

A snapshot of the code is also available in the *GigaScience* GigaDB repository [79].

List of abbreviations

ABCD standard: Access to Biological Collections Data standard; AWT: ARPHA Writing Tool; BOLD: Barcode of Life Data Systems; CSV file: comma-separated value file; DiSSCo: Distributed System of Scientific Collections; DwC: Darwin Core Standard; ENA: European Nucleotide Archive; FAIR data: Findable, Accessible, Interoperable and Re-usable data; FSK-M: Food Safety Knowledge Markup Language; GBIF: Global Biodiversity Information Facility; GeOMe: Genomic Observatories Metadatabase; GGBN: Global Genome Biodiversity Network; GMI: Global Microbial Identifier; GSC: Genomics Standards Consortium; iDigBio: Integrated Digitized Biocollections; INSDC: International Nucleotide Sequence Database Collaboration; IPT: Integrated Publishing Toolkit; JATS: Journal Article Tag Suite; LTER: Long Term Ecological Research Network; MetaShARK: Metadata Shiny Automated Resources and Knowledge; MIAME: Minimum Information About a Microarray Experiment; MINSEQE: Minimum Information about a high-throughput nucleotide SEQuencing Experiment; MIxS: Minimum Information about any (x) Sequence; MS: mass spectrometry; OBIS: Ocean Biogeographic Information System; OTU: operational taxonomic units

Competing interests

MD is a PhD student at Pensoft through the EU funded project IGNITE and one of her key tasks during the PhD project is to develop methods for publication, dissemination and re-use of biodiversity-related genomics data. TG, GZ, SD and LP are employed by Pensoft and LP also holds a professorship position in the Bulgarian Academy of Sciences. Pensoft's Biodiversity Data Journal has implemented the genomics data paper workflow in its routine editorial practices.

Funding

This research has received funding from the European Union's Horizon 2020 research and innovation programme under the Marie Sklodowska-Curie grant agreement IGNITE (No 764840) and from Pensoft Publishers.

Supplementary data

Additional File 1

This is an HTML file demonstrating the structure of the omics data paper template in the Arpha Writing Tool.

- html file

References

1. Hey T, Trefethen A, The Data Deluge: An e-Science Perspective. In: Berman, F, Fox, G C and Hey, A J G, eds. *Grid Computing - Making the Global Infrastructure a Reality*. Wiley and Sons; 2003:809-824.
2. Perez-Riverol Y, Zorin A, Dass G, Vu M, Xu P, Glont M, Vizcaíno J, Jarnuczak A, Petryszak R, Ping P, Hermjakob H. Quantifying the impact of public omics data. *Nat Commun*. 2019;10(1).
3. Darwin Tree Of Life. 2020. https://www.darwintreeoflife.org/. Accessed 8 June 2020.
4. Earth BioGenome Project. 2020. https://www.earthbiogenome.org/. Accessed 8 June 2020.
5. Fondation Tara Océan. 2020. https://oceans.taraexpeditions.org/en/. Accessed 8 June 2020.
6. Schriml L, Chuvochina M, Davies N, Eloe-Fadrosh E, Finn R, Hugenholtz P, Hunter C, Hurwitz B, Kyrpides N, Meyer F, Mizrachi I, Sansone S, Sutton G, Tighe S, Walls R. COVID-19 pandemic reveals the peril of ignoring metadata standards*. Scientific Data*. 2020;7(1); https://doi.org/10.1038/s41597-020-0524-5
7. Karsch-Mizrachi I, Takagi T, Cochrane G. The international nucleotide sequence database collaboration. *Nucleic Acids Res*. 2017;46(D1), pp.D48-D51.
8. Stevens H. Globalizing Genomics: The Origins of the International Nucleotide Sequence Database Collaboration. *J Hist Biol.* 2018*;*51, 657–69; https://doi.org/10.1007/s10739-017-9490-y
9. Thessen A, Patterson D. Data issues in the life sciences. *ZooKeys*. 2011;150, pp.15-51.
10. GBIF: The Global Biodiversity Information Facility. 2020. What is GBIF?. https://www.gbif.org/what-is-gbif. Accessed 25 June 2020.
11. iDigBio. 2020. https://www.idigbio.org/. Accessed 25 June 2020.
12. DiSSCo. 2020. https://www.dissco.eu/. Accessed 25 June 2020.
13. OBIS. 2020. www.iobis.org. Accessed 25 June 2020.
14. Droege G, Barker K, Astrin J, Bartels P, Butler C, Cantrill D, Coddington J, Forest F, Gemeinholzer B, Hobern D, Mackenzie-Dodds J, Ó Tuama É, Petersen G, Sanjur O, Schindel D, Seberg O. The Global Genome Biodiversity Network (GGBN) Data Portal. *Nucleic Acids Res*. 2013;42(D1), pp.D607-D612.
15. DataONE. 2020. https://www.dataone.org. Accessed 24 November 2020.
16. GBIF Data standards. 2020. https://www.gbif.org/standards. Accessed 24 November 2020.

17. Wieczorek J, Bloom D, Guralnick R, Blum S, Döring M, Giovanni R, Robertson T, Vieglais D. Darwin Core: An Evolving Community-Developed Biodiversity Data Standard. *PLoS ONE*. . 2012; 7(1), p.e29715. https://doi.org/10.1371/journal.pone.0029715
18. What Is Darwin Core, And Why Does It Matter?. 2020. Gbif.org. https://www.gbif.org/darwin-core. Accessed 15 July 2020.
19. Droege G, Barker K, Seberg O, Coddington J, Benson E, Berendsohn W, Bunk B, Butler C, Cawsey E, Deck J, Döring M, Flemons P, Gemeinholzer B, Güntsch A, Hollowell T, Kelbert P, Kostadinov I, Kottmann R, Lawlor R, Lyal C, Mackenzie-Dodds J, Meyer C, Mulcahy D, Nussbeck S, O'Tuama É, Orrell T, Petersen G, Robertson T, Söhngen C, Whitacre J, Wieczorek J, Yilmaz P, Zetzsche H, Zhang Y, Zhou X. The Global Genome Biodiversity Network (GGBN) Data Standard specification. *Database*. 2016; p.baw125.
20. Holetschek J, Dröge G, Güntsch A, Berendsohn W 2012. The ABCD of primary biodiversity data access. *Plant Biosystems*. 2012; 146(4), pp.771-779.
21. Field D, Amaral-Zettler L, Cochrane G, Cole J, Dawyndt P, Garrity G, Gilbert J, Glöckner F, Hirschman L, Karsch-Mizrachi I, Klenk H, Knight R, Kottmann R, Kyrpides N, Meyer F, San Gil I, Sansone S, Schriml L, Sterk P, Tatusova T, Ussery D, White O, Wooley J. The Genomic Standards Consortium. *PLoS Biology*. 2011; 9(6), p.e1001088.
22. Forside - Global Microbial Identifier. 2020. http://www.globalmicrobialidentifier.org/. Accessed 15 July 2020.
23. Yilmaz P, Kottmann R, Field D, Knight R, Cole J, Amaral-Zettler L, et al. Minimum information about a marker gene sequence (MIMARKS) and minimum information about any (x) sequence (MIxS) specifications. *Nature Biotechnology*. 2011;29(5), pp.415-420.
24. Athar A, Füllgrabe A, George N, Iqbal H, Huerta L, Ali A, et al. ArrayExpress update – from bulk to single-cell expression data. *Nucleic Acids Res. 2018;*47(D1), pp.D711-D715.
25. Biosamples. 2020. https://www.ebi.ac.uk/biosamples/. Accessed 21 May 2020.
26. FGED: MINSEQE. 2020. http://fged.org/projects/minseqe/. Accessed 25 June 2020.
27. GEOME. 2020. https://geome-db.org/. Accessed 24 November 2020.
28. Deck J, Gaither M R, Ewing R, Bird C E, Davies N, et al. The Genomic Observatories Metadatabase (GeOMe): A new repository for field and sampling event metadata associated with genetic samples. *PLOS Biology*. 2017;15(8): e2002925. https://doi.org/10.1371/journal.pbio.2002925
29. Sansone S A, Rocca-Serra P, Field D, et al. Toward interoperable bioscience data. *Nat Genet.* 2012;44, 121–126. https://doi.org/10.1038/ng.1054
30. Rocca-Serra P, Brandizi M, Maguire E, Sklyar N, Taylor C, Begley K, et al. ISA software suite: supporting standards-compliant experimental annotation and enabling curation at the community level. *Bioinformatics.* 2010;*26*(18), 2354–2356. https://doi.org/10.1093/bioinformatics/btq415

31. Haug K, Cochrane K, Nainala V C, Williams M, Chang J, Jayaseelan K V, O'Donovan, C. MetaboLights: a resource evolving in response to the needs of its scientific community. *Nucleic Acids Res*. 2019; https://doi.org/10.1093/nar/gkz1019
32. Sneddon T, Si Zhe X, Edmunds S, Li P, Goodman L, Hunter C. GigaDB: promoting data dissemination and reproducibility. *Database*. 2014; https://doi.org/10.1093/database/bau018
33. FORCE11. 2016. https://www.force11.org/group/fairgroup/fairprinciples. Accessed 12 June 2020.
34. Sansone S, McQuilton P, Rocca-Serra P, et al. FAIRsharing as a community approach to standards, repositories and policies. *Nat Biotechnol*. 2019;37, 358–367. https://doi.org/10.1038/s41587-019-0080-8
35. Chavan V, Penev L. The data paper: a mechanism to incentivize data publishing in biodiversity science. *BMC Bioinformatics*.2011;12(S15). https://doi.org/10.1186/1471-2105-12-S15-S2
36. Earth System Science Data. 2020. http://www.earth-syst-sci-data.net/. Accessed 7 July 2020.
37. Five years of Scientific Data. *Sci Data.* 2019;6, 72. https://doi.org/10.1038/s41597-019-0065-y
38. Penev L, Mietchen D, Chavan V, Hagedorn G, Remsen D, Smith V, Shotton D. Pensoft Data Publishing Policies and Guidelines for Biodiversity Data. Pensoft Publishers. 2011; http://www.pensoft.net/J_FILES/Pensoft_Data_Publishing_Policies_and_Guidelines.pdf.
39. Penev L, Mietchen D, Chavan V, Hagedorn G, Smith V, Shotton D, Ó Tuama É, Senderov V, Georgiev T, Stoev P, Groom Q, Remsen D, Edmunds S. Strategies and guidelines for scholarly publishing of biodiversity data. *Research Ideas and Outcomes*. 2017;3, p.e12431.
40. Penev L, Georgiev T, Geshev P, Demirov S, Senderov V, Kuzmova I, Kostadinova I, Peneva S, Stoev P. ARPHA-BioDiv: A toolbox for scholarly publication and dissemination of biodiversity data based on the ARPHA Publishing Platform. *Research Ideas and Outcomes.* 2017;3: e13088. https://doi.org/10.3897/rio.3.e13088
41. Pensoft blog. 2020. *How To Import Data Papers From GBIF, Dataone And LTER Metadata*. https://blog.pensoft.net/2016/05/18/how-to-import-data-papers-from-gbif-data-one-and-lter-metadata/. Accessed 24 November 2020.
42. Filter M, Candela L, Guillier L, Nauta M, Georgiev T, Stoev P, Penev L. Open Science meets Food Modelling: Introducing the Food Modelling Journal (FMJ). *Food Modelling Journal.* 2019;1: e46561. https://doi.org/10.3897/fmj.1.46561

43. The ENA Metadata Model. 2020. https://ena-docs.readthedocs.io/en/latest/submit/general-guide/metadata.htm. Accessed 5 May 2020.
44. Carpenter E, Matasci N, Ayyampalayam S, Wu S, Sun J, Yu J, et al. Access to RNA-sequencing data from 1,173 plant species: The 1000 Plant transcriptomes initiative (1KP). *GigaScience.* 2019; 8(10).
45. Filho J, Jorge S, Kremer F, de Oliveira N, Campos V, da Silva Pinto L, et al. Complete genome sequence of native Bacillus cereus strains isolated from intestinal tract of the crab Ucides sp. *Data in Brief*. 2018;16, pp.381-385.
46. Zhou Y, Xiao S, Lin G, Chen D, Cen W, Xue T, et al. Chromosome genome assembly and annotation of the yellowbelly pufferfish with PacBio and Hi-C sequencing data. *Scientific Data*. 2019; 6(1).
47. Programmatic Access To ENA Data. 2020. https://www.ebi.ac.uk/ena/browse/programmatic-access. Accessed 21 November 2020.
48. Winston Chang, Joe Cheng, JJ Allaire, Yihui Xie, Jonathan McPherson (2020). shiny: Web Application Framework for R. R package version 1.5.0. http://shiny.rstudio.com
49. RStudio, PBC (2020). Shinyapps.io. Boston, Massachusetts, USA: RStudio, PBC.
50. R Core Team (2020). R: A language and environment for statistical computing. R Foundation for Statistical Computing, Vienna, Austria. URL https://www.R-project.org/.
51. National Information Standards Organization, 2019. ANSI/NISO Z39.96-2019, JATS: Journal Article Tag Suite, Version 1.2 | NISO Website. Niso.org. https://www.niso.org/publications/z3996-2019-jats. Accessed 10 August 2020.
52. Fay C, Guyader V, Rochette S, Girard C (2020). golem: A Framework for Robust Shiny Applications. R package version 0.3.0. https://github.com/ThinkR-open/golem
53. Dimitrova M (2020). omicsdatapaper: OMICS data paper R shiny app as golem. GitHub. https://github.com/pensoft/omicsdatapaper.
54. Dimitrova M (2020). *Omics Data Paper Generator*. https://mdmtrv.shinyapps.io/Omics_data_paper/.
55. Dimitrova M (2020). Pensoft/Omics-Data-Paper-Shinyapp. https://github.com/pensoft/omics-data-paper-shinyapp.
56. RStudio, PBC (2020). Rstudio.Cloud. Boston, Massachusetts, USA: RStudio, PBC.
57. Penev L, Dimitrova M, Kostadinova I, Georgiev T, Agosti D, Poelen J. 2020. *How To Get Data From Research Articles Back Into The Research Cycle At No Additional Costs?*. https://blog.pensoft.net/2020/04/24/how-to-get-data-from-research-articles-back-into-the-research-cycle-%D0%B0t-no-additional-costs/. Accessed 24 November 2020.
58. Biodiversity Data Journal Data Publishing Guidelines: Linked data table for primary biodiversity data. 2020.

https://bdj.pensoft.net/about#Linkeddatatableforprimarybiodiversitydata. Accessed 24 November 2020.
59. Arrayexpress - Data Access Policy. 2020. https://www.ebi.ac.uk/arrayexpress/help/data_availability.html. Accessed 24 November 2020
60. JATS4R Validator. 2020.. https://validator.jats4r.org/. Accessed 18 August 2020.
61. ESA's Ecological Archives. 2020. http://www.esapubs.org/archive/default.htm. Accessed 5 November 2020.
62. Smith M. Data Papers in the Network Era. In: *Charleston Library Conference*. *Against the Grain Press, LLC*. 2011; http://dx.doi.org/10.5703/1288284314871.
63. Robertson T, Döring M, Guralnick R, Bloom D, Wieczorek J, Braak K, et al. The GBIF Integrated Publishing Toolkit: Facilitating the Efficient Publishing of Biodiversity Data on the Internet. *PLoS ONE*. 2014; 9(8), p.e102623.
64. Data Papers. 2020. https://www.gbif.org/data-papers. Accessed 5 May 2020.
65. Schöpfel J, Farace D, Prost H, Zane A. Data papers as a new form of knowledge organization in the field of research data. *12ème Colloque international d'ISKO-France :Données et mégadonnées ouvertes en SHS : de nouveaux enjeux pour l'état et l'organisation des connaissances ?, ISKO* France, Oct 2019, Montpellier, France. halshs-02284548

66. Smith V, Georgiev T, Stoev P, Biserkov J, Miller J, Livermore L, et al. Beyond dead trees: integrating the scientific process in the Biodiversity Data Journal. *Biodiversity Data Journal*. 2013;1, p.e995. https://doi.org/10.3897/BDJ.1.e995
67. Senderov V, Georgiev T, Penev L. Online direct import of specimen records into manuscripts and automatic creation of data papers from biological databases. *Research Ideas and Outcomes*. 2016;2, p.e10617
68. Arnaud E (2020). MetaShARK-v2. https://github.com/earnaud/MetaShARK-v2.
69. Sansone S A, Rocca-Serra P, Izzo M. 2020. https://datascriptor.org/. Accessed 19 August 2020.
70. Martens L, Vizcaíno J. A Golden Age for Working with Public Proteomics Data. *Trends in Biochemi*cal Sciences. 2017;42(5), pp.333-341.
71. Biodiversity Data Journal Data Quality Checklist And Recommendations. 2020. https://bdj.pensoft.net/about#DataQualityChecklistandRecommendations. Accessed 18 August 2020.
72. All Is FAIR In Biodiversity Research: Mandatory Data Audit At Pensoft's Journals. 2019. https://www.eurekalert.org/pub_releases/2019-10/pp-aif101819.php. Accessed 22 January 2021.
73. Biodiversity Data Journal Data Review Guidelines. 2020. https://bdj.pensoft.net/about#DataReviewGuidelines. Accessed 22 January 2021.
74. Kolesnikov N, et al. ArrayExpress update-simplifying data submissions. *Nucleic Acids Res*. 2015; doi:10.1093/nar/gku1057.
75. Arrayexpress/Annotare 2.0. 2012. https://github.com/arrayexpress/annotare2
76. Penev L, Dimitrova M, Senderov V, Zhelezov G, Georgiev T, Stoev P, Simov K. OpenBiodiv: A Knowledge Graph for Literature-Extracted Linked Open Data in Biodiversity Science. *Publications*. 2019; 7(2):38. https://doi.org/10.3390/publications7020038
77. Xue H, Li J, Xie H, Wang Y. Review of Drug Repositioning Approaches and Resources. *International Journal of Biological Sciences*. 2018;14(10), pp.1232-1244.
78. McDonald D, Clemente J, Kuczynski J, Rideout J, Stombaugh J, Wendel D, et al. The Biological Observation Matrix (BIOM) format or: how I learned to stop worrying and love the ome-ome. *GigaScience*. 2012;1(1).

79. Dimitrova M; Meyer R; Buttigieg PL; Georgiev T; Zhelezov G; Demirov S; Smith V; Penev L (2021): Supporting data for "A streamlined workflow for conversion, peer review and publication of genomics metadata as Omics Data Papers" GigaScience Database. http://dx.doi.org/10.5524/100889

Figure 1

Click here to access/download;Figure;figure_1.pdf

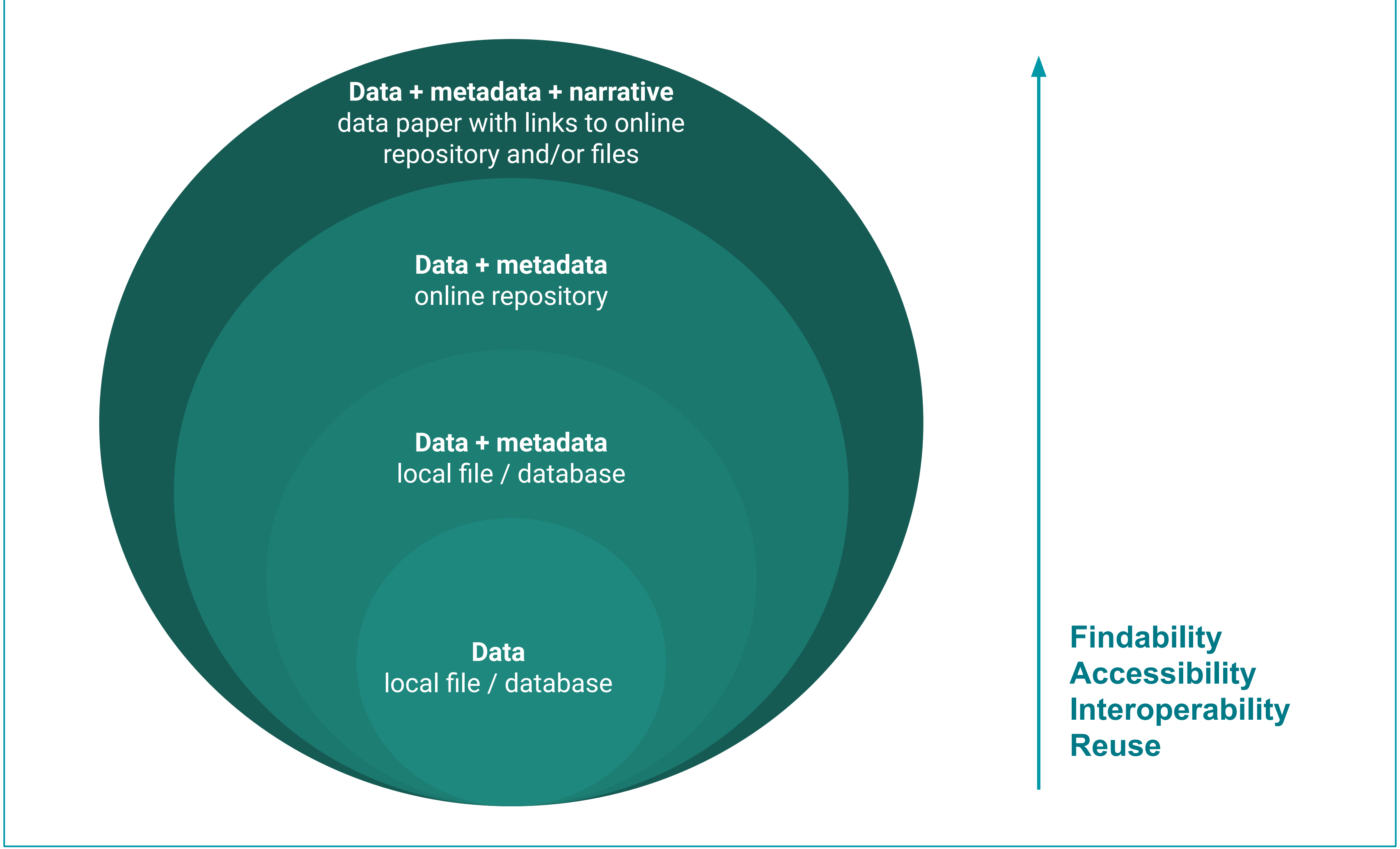

Figure 2

Click here to access/download;Figure;figure_2.pdf

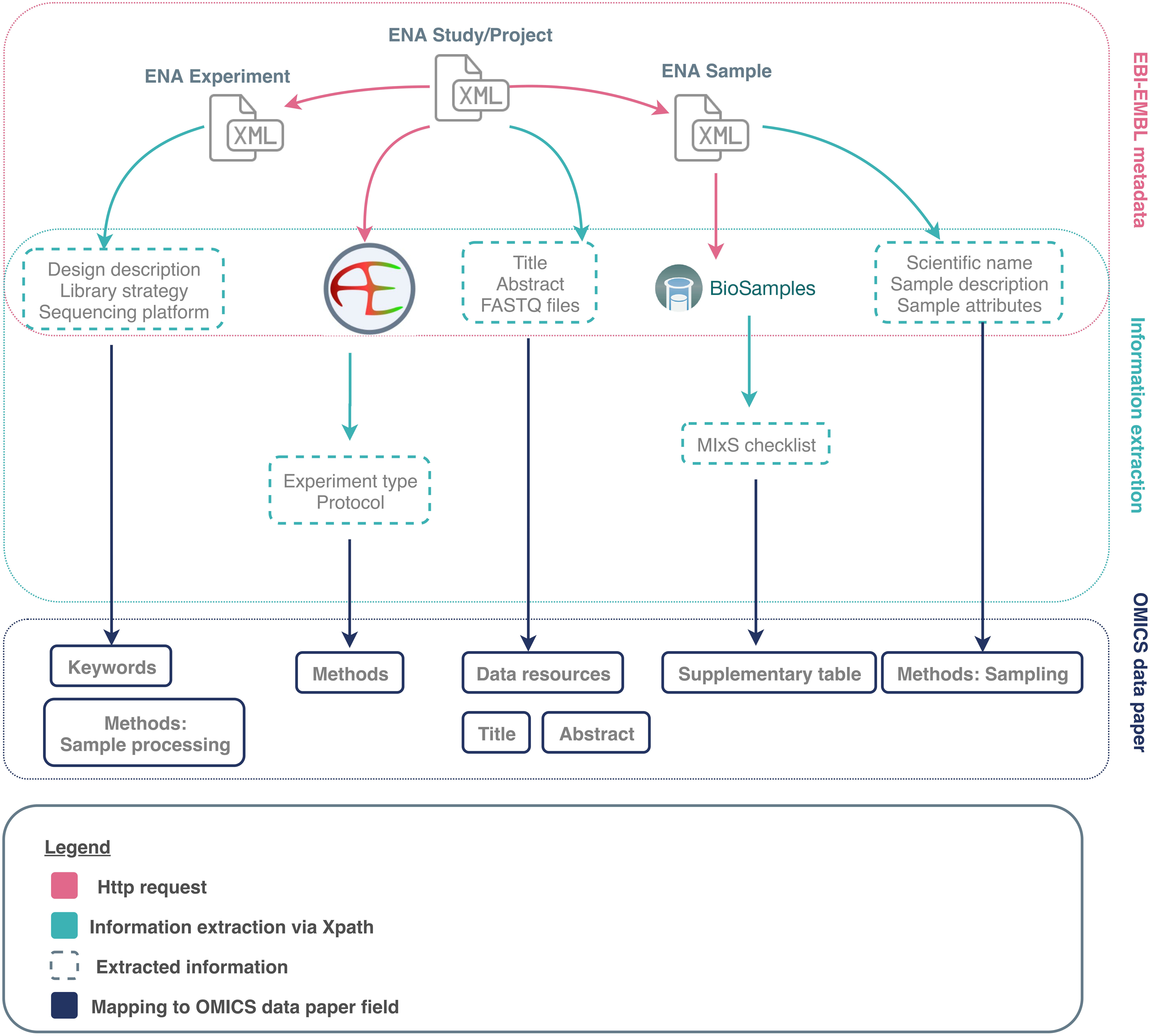

Figure 3

Click here to access/download;Figure;figure_3.pdf

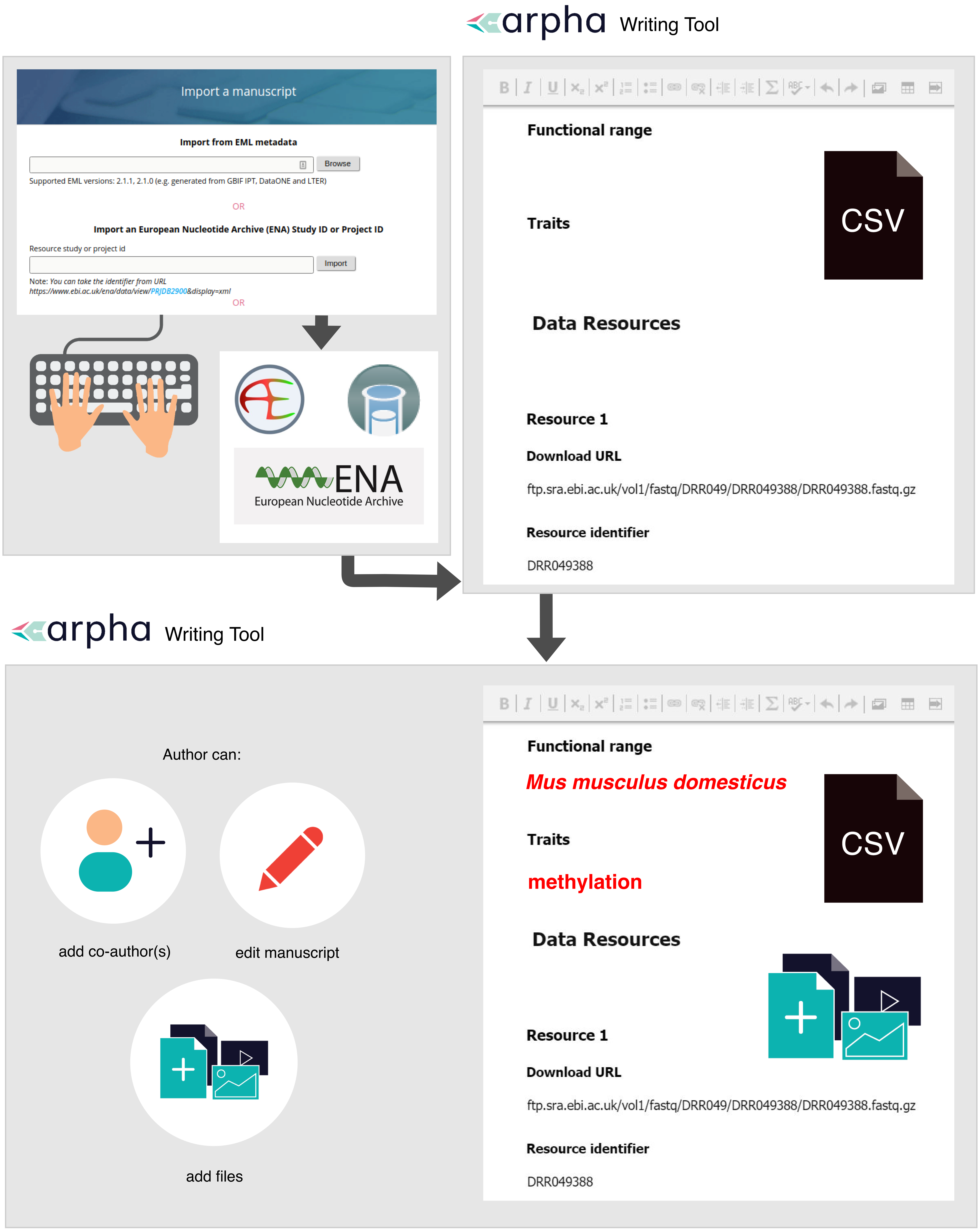

Manuscript

Click here to access/download
Supplementary Material
Additional_file_1.html

Supplemental XML example

Click here to access/download
Supplementary Material
Additional_file_1(1).html

Click here to access/download;Personal Cover;Response_to_editor_and_reviewers.pdf

Dear Editor and Reviewers,

Thank you for taking the time and effort to review our manuscript again! We highly appreciate your constructive feedback in this and the previous review rounds. Your thoughtful comments helped us make some important changes.

You advised us to submit the R shiny app to https://bio.tools and https://SciCrunch.org databases to receive RRID (Research Resource Identification Initiative ID) and biotoolsID identifiers and we have done so. The identifiers are: biotools:omics-data-paper-shinyapp-golem for biotools and SCR_019809 for SciCrunch.org. We have updated the manuscript with these identifiers in the "Availability of supporting source code and requirements" section on page 28.

Below you will find an explanation of all other modifications we made to the manuscript, as well as detailed responses to your comments.

Question 1: Do data paper undergo peer review?
if so, what is the acceptance rate?
Scientists claims that they are over burden with data management tasks, from data deposition to manuscript writing.
Could the authors indicate how much work a data paper represents in contrast to a classic scientific article?

Response:

Similarly to all other manuscript types in Pensoft journals, all data papers undergo a rigorous peer review (see https://bdj.pensoft.net/about#DataReviewGuidelines). In addition, prior to peer review they are also subjected to data auditing by a data auditor at Pensoft who makes sure that data is accessible and its quality is of high standard according to a data quality checklist (https://bdj.pensoft.net/about#DataQualityChecklistandRecommendations). The submitted manuscript contains information about the peer review and data audit of the genomics data papers. We have indicated Pensoft's data auditing and peer-review processes in the "Omics data papers and underlying datasets undergo peer-review and data auditing" subsection of the Discussion section on page 23.

The acceptance rate of the Biodiversity Data Journal varies between 70-80%.

In terms of the effort that data papers require from authors, it really depends on the nature of the data and the author's preference for including a certain number of data statistics and meta-analyses to describe their data. Data papers do not require authors to include an analysis of the dataset but they can do so if they wish, as long

as the data paper does not become too long-drawn-out. The key aspect of a data paper is the detailed description of the data following the community accepted standards and FAIRness and quality of the data itself, therefore authors' main efforts should go towards making sure that the described datasets are consistent throughout, well-described in terms of methodology and openly available and accessible. The goal of the data paper is to make the dataset fully reusable so that other researchers can repeat the experiment or collate the published data with other datasets to generate new hypotheses and research results.

The benefits of publishing data papers are listed extensively in the section "Data papers for the field of omics: rationale and benefits" (page 22) of the manuscript. In the first version of the manuscript, we had included a figure (enclosed below as a part of our response to the comment) which shows a clear increasing trend in popularity and use of data papers as a useful method of data publishing and a powerful instrument for improving research integrity and data reuse. For example the Biodiversity Data Journal alone, shows the following numbers in publication of data papers: 2018 (9), 2019 (31). For 2020 (not pictured in the figure), there were 68 data papers, which indicates an yearly increase of more than 100 % in the last year alone.

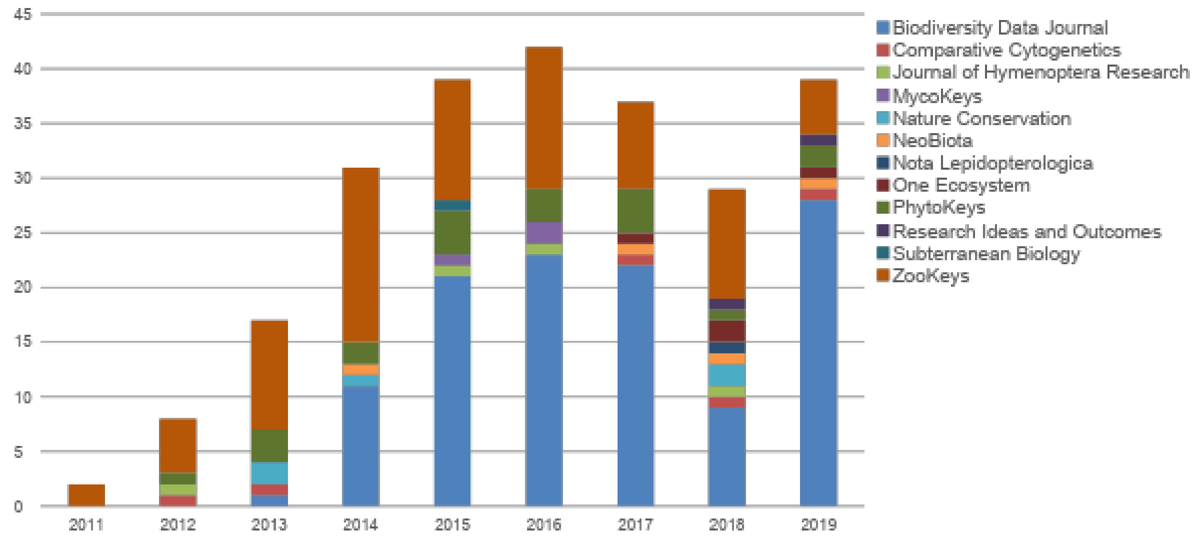

Fig. 1 Number of data papers published in Pensoft's journals (2011-2019)

Question 2: Following creation of the populated backbone, how much write-up are authors expected to do to reach minimal content and quality requirements?
I understand the authors somehow tackle this issue on pages 22-26 but I am still concerned about the economics and sustainability of the approach.
The authors indicate that OMIC data paper are audited, which means curation time is expanded. This is an expensive task to accomplish and requires domain experts.

It would be interesting to cover some of these aspects in the discussion, all the more so finding reviewers is becoming harder and harder (which would be also require finding data experts).

Response:

Data auditing is indeed an expensive task but Pensoft strives towards publishing high quality datasets and has fully committed to this practice, provided by **in-house data auditors**. The data auditors are biologists with extensive experience with data handling, storing and processing. They ensure that the datasets follow some basic guidelines to ensure their FAIRness and consistency. They provide authors with tips on how to make their data FAIR, if it is not FAIR yet. Data auditors do not reproduce data generation and analysis if there is such. This would be the role of peer-reviewers. Finally, data auditors are permanently employed and unlike peer-reviewers do not have to be sought out on a case by case basis, which significantly reduces the waiting time. Therefore, we cannot really compare the process of finding reviewers with the process of finding data auditors. We have included a paragraph in the manuscript about how the system works ("Omics data papers and underlying datasets undergo peer-review and data auditing" subsection on page 23), with the relevant citations of the respective data quality guidelines of the Biodiversity Data Journal[1,2]. Perhaps, the intricacies of data paper publishing, including finding data auditors, can be explored in more detail in a separate publication but here we wanted to focus on genomic data papers and the workflow. More information about the data auditing process at Pensoft can be found in this blog (also cited in the discussion part of the paper): https://www.eurekalert.org/pub_releases/2019-10/pp-aif101819.php

Question 3: Is there a specific mechanism used in Pensoft OMIC Data Paper to reference database accession numbers so those can be easily identified and extracted?

Response:

Database accession numbers have to be referenced in the Data Resources section of the omics data paper (Fig. 2). The Resource identifier field, where such an accession number should be entered, is a required field of the template, meaning that it cannot be submitted without being filled in. If using the omics data paper conversion workflow, this field is automatically populated with an ENA sequence identifier.

---

[1] https://bdj.pensoft.net/about#DataQualityChecklistandRecommendations
[2] https://bdj.pensoft.net/about#DataReviewGuidelines

After publication, all articles in BDJ are publicly available also as JATS XML and submitted as such for archiving and display to PubMedCentral, in addition to the traditional PDF and HTML formats. Therefore, all identifiers and links to external databases are marked up in the XML version of the article and can then be indexed by Web crawlers, including Pensoft's own RDFization scripts which transform XML articles to Resource Description Framework (RDF) to ensure machine readability of the text ([Penev et al., 2019](#)).

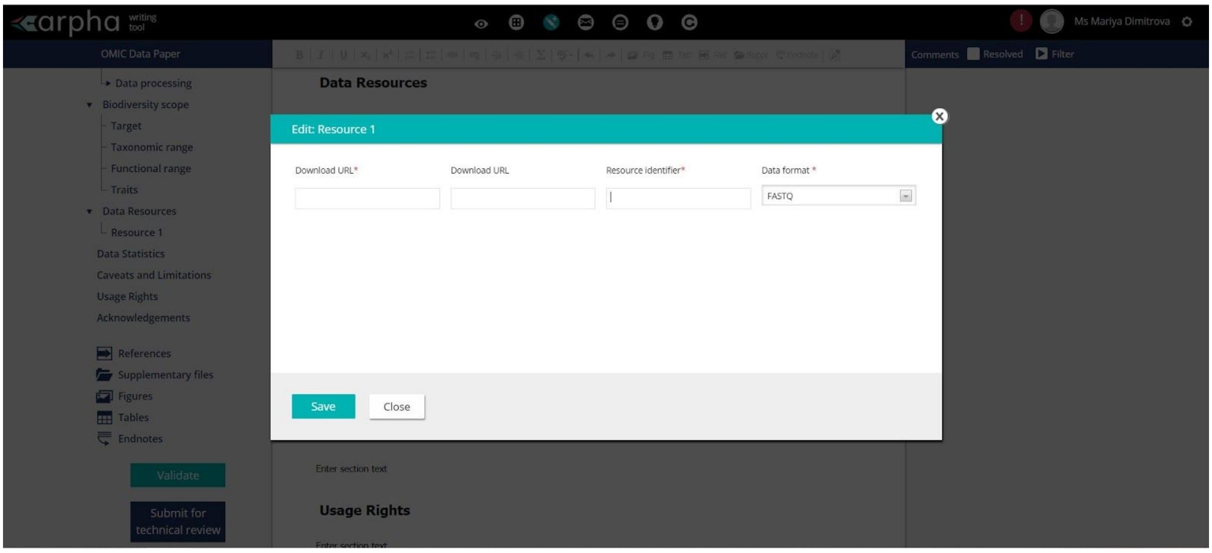

Fig. 2 Editing the "Data Resources" section in the Arpha Writing Tool - a screenshot

Question 4: Have the authors also considered an automated action to update a database (e.g. ENA) record with a DOI associated with a Pensoft `OMIC data paper`? This comes back to the issue of producing an entirely new artifact, possibly one containing 90 % of the information stored in a SRA XML document but presented in a JATS documents.
Were EMBL-EBI records to be given DOIs, would it impact the OMIC Data Paper model ?

Response: We have considered this possibility as an excellent way to link data and literature both ways. There were some preliminary discussions with ENA to at least provide a back linking from the published data paper to the relevant resource at ENA and we expect to achieve that during the starting EU-funded BiCIKL project, coordinated by Pensoft. During that project, EMBL-EBI should provide an interface to edit/improve the metadata of already submitted datasets from annotations to these, data papers included, posted *post factum*.

Pensoft has a proven experience in this kind of workflow with GBIF, For example, the discrepancies in data already indexed in GBIF found by our data auditors are sent to the authors with a clear demand to correct the data in the GBIf indexed dataset before the manuscript will be forwarded to peer review in the journal.

If EMBL-EBI records are given (DataCite) DOIs, which of no doubt would be an excellent practice, this would not create any issues for the workflow because they would be just different identifier records than the data papers. This is because the original dataset is a separate entity than the data paper in which it is described.

Question 5: Dealing with large datasets.

Some studies may contain hundreds, thousands of samples. The interaction with the AWT indicates that the OMIC data article would contain a long list of biomaterials, and associated resources.

Have the authors considered aspect of stacking / compressing information or have they considered automated sentence generation to assist authors in expanding the text for an OMIC data paper ?

Response:

We have thought about large datasets and this is why we attach the BioSamples checklist as a supplementary file and not as a table within the narrative itself. When it comes to sentence generation, this is not really needed because we do not require separate description of each and every sample. Authors can aggregate their description of large datasets to create a concise and clear manuscript.

Actually, the sample size does not directly affect the size of the data paper, because the data are stored outside the journal's infrastructure, which hosts the narrative of the data paper. Large data sets would require a bit more detailed text description and explanations, however this is a normal practice in the manuscript authoring and publishing process.

Question 6. License on the data

Shouldn't there be a filled in section in the OMIC data paper to specific the nature of the license under which data and resources are made available?
this is possible to auto-generated from public repositories and, if authors create a manuscript from scratch, they should be offered options.
This is distinct from the license attached to the manuscript itself or to the metadata.

Response: There is already such a section, called "Usage rights", where authors can specify the license of the data. It is implemented both in Pensoft's ARPHA template and in the R shiny app (Fig. 3).

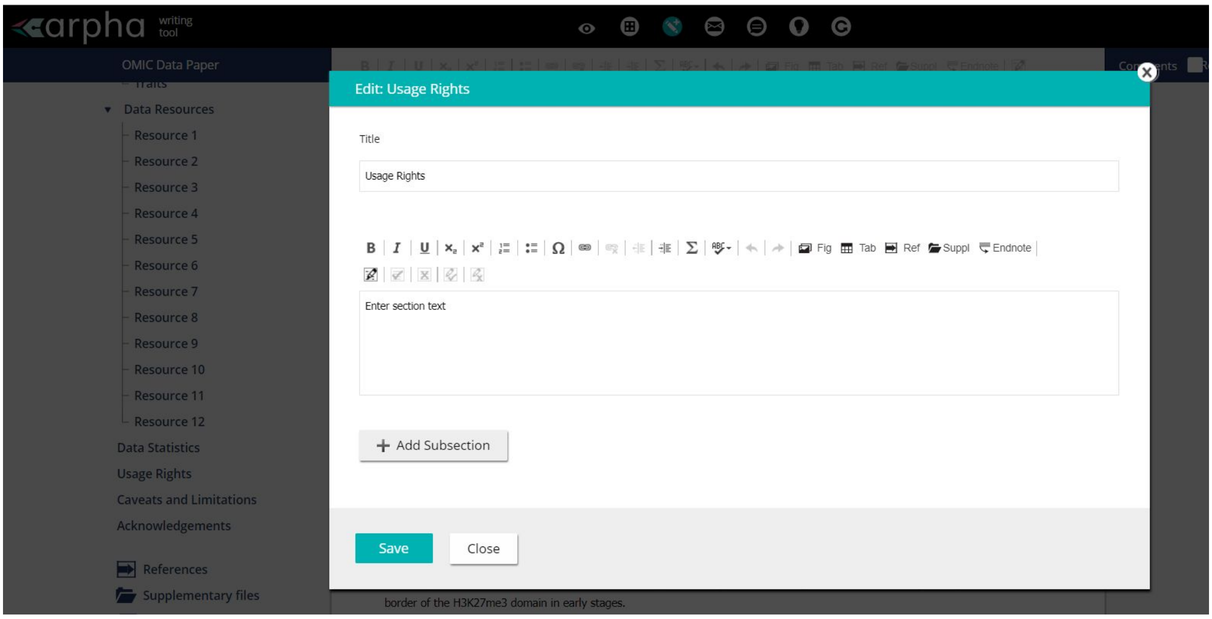

Fig. 3 “Usage rights” section in ARPHA Writing Tool.

We have not implemented a dropdown menu or other kind of entry form with limited options for licenses because there might be a case in which different datasets, described in the data paper, have different (open-source) licenses so it would be best if the authors specify that manually. As stated in the author guidelines of BDJ, only datasets with open-source licenses can be published as part of data paper publications in Pensoft’s journals (Fig. 4).

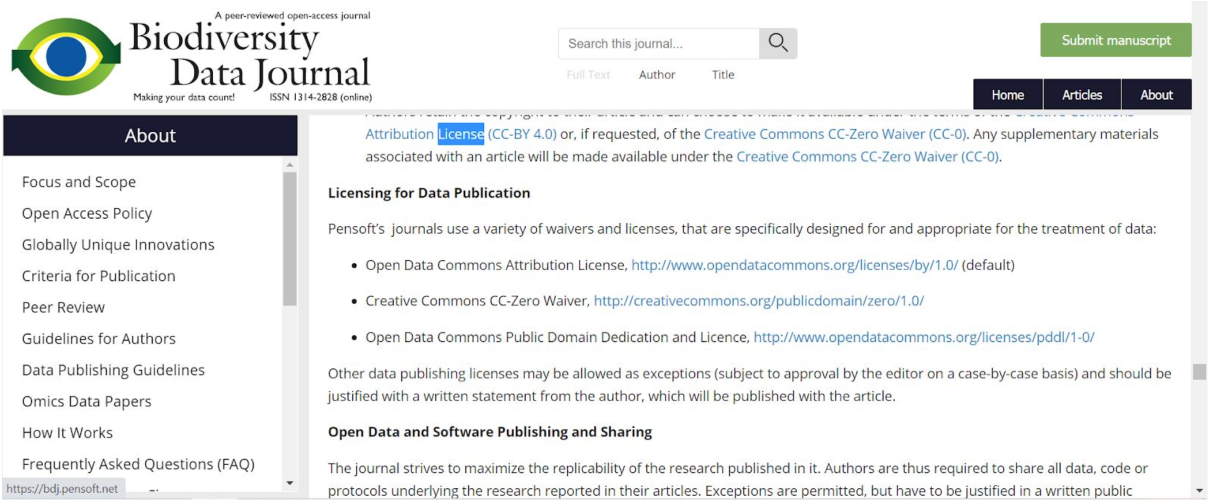

Fig. 4 Data licensing guidelines as part of the Pensoft Author Guidelines

----

minor corrections:

page 5, "MIxS consists of three checklists, <comma inserted> each containing several packages for the description of various environments where

genomic material could be sampled from [22]."

- >overall, check for similar missing elements of punctuation.

Response:
We have corrected this mistake and have checked for other punctuation mistakes throughout the manuscript.

page 6: "A more comprehensive approach towards omics metadata mobilisation is undertaken
by the ISA Commons community [28], who ",

-> s/who/which/

Response:

We have corrected this in the manuscript.

page 6: "(1) data publishing through international trusted data repositories, such as INSDC [7], GBIF [9], and others, a.. "

-> drop "and others" to simplify the sentence.

Response:

We have corrected this in the manuscript.

page 7: "Furthermore, it ensures a scientific record, crediting and acknowledgement for the data creators and scientists in the form of citable scholarly articles."

rephrase or complete, eg: "Furthermore, it ensures that a citable scholarly scientific record, crediting and acknowledging the daat creators.... is created'.

Response:

We have rephrased this sentence to make it more clear while preserving the meaning. The new sentence is: “Furthermore, it creates a citable scientific record, enabling the crediting and acknowledgement of the data creators and researchers.”

page 7: "As more and more researchers want to deposit and share their datasets, standards, infrastructures, and workflows become central to delivering FAIR data.

rephrase, eg: "As more and more researchers want to deposit and share their datasets, new tools and new approaches are needed to deliver FAIR data.

Response:

We have rephrased this sentence according to your suggestion.

page 8: "to describe a prototyped workflow"
-> s/prototyped/prototype/

Response:

We have corrected this sentence in the manuscript.

page 9: "We created a template, "
-> "We created a dedicated data article template, "

Response:

We have corrected this sentence in the manuscript to “We created a dedicated data paper template”...

page 23, section 1: "is an meticulous approach to", s/an/a/

Response:

We have corrected this mistake in the manuscript.

page 24, section 2: "Throughout our testing phase", add a comma after 'phase'

Response:

We have corrected this mistake in the manuscript.
