# Supplementary material for: A streamlined workflow for conversion, peer review, and publication of genomics metadata as omics data papers
Source: Gigascience. 2021 May 13;10(5):giab034. doi: 10.1093/gigascience/giab034 (PMC8117446; doi:10.1093/gigascience/giab034)
Supplement: giab034_Supplemental_Files [file giab034_supplemental_files.zip › Additional_file_1(1).html]

ARPHA Writing Tool


  


OMIC Data Paper

OMICS DATA PAPER TEMPLATE

- Article metadata

  - Title
  - Abstract
  - Keywords
  - Funder
  - Funding program
  - Grant title
  - Hosting institution
  - Ethics and security
  - Author contributions
  - Conflicts of interest
- Add section

  - Subsection
- Introduction

  - Value of the dataset
  - Subsection
- Methods

  - Sampling
  - Sample processing
  - Data processing
  - Subsection
- Biodiversity scope

  - Target
  - Taxonomic range
  - Functional range
  - Traits
  - Subsection
- Data Statistics

  - Subsection
- Caveats and Limitations

  - Subsection
- Usage Rights

  - Subsection
- Data Resources

  - Resource
- Add section

  - Subsection
- Acknowledgements
- References
- Supplementary files
- Figures
- Tables
- Endnotes

|  |  |
| --- | --- |
| Journal : OMICS DATA PAPER TEMPLATE |  |

TODO: Additional information about the template

# OMICS DATA PAPER TEMPLATE

First Author
‡

‡ Affiliation, City, Country

|  |  |
| --- | --- |
| Corresponding author: First Author (corresponding@author.email)  © 2015 First Author.  Citation:  Author F (2015) Article Title. Journal Title volume: pages. doi: doi\_number |  |

# Abstract

Lorem ipsum dolor sit amet, consectetur adipiscing elit. Nullam vel dolor tincidunt, fermentum velit a, luctus est. Aliquam erat volutpat. Vestibulum ante ipsum primis in faucibus orci luctus et ultrices posuere cubilia Curae; In hac habitasse platea dictumst. Morbi dolor nibh, ultricies sit amet nisl quis, suscipit porttitor lacus. Curabitur eu mauris ac mauris blandit dapibus. Cras vitae arcu nec lectus volutpat consectetur. Integer condimentum eget libero nec ultrices.

# Keywords

Curabitur, mauris, blandit, dapibus

# Add section

Lorem ipsum dolor sit amet, consectetur adipiscing elit. Nullam vel dolor tincidunt, fermentum velit a, luctus est. Aliquam erat volutpat. Vestibulum ante ipsum primis in faucibus orci luctus et ultrices posuere cubilia Curae; In hac habitasse platea dictumst. Morbi dolor nibh, ultricies sit amet nisl quis, suscipit porttitor lacus. Curabitur eu mauris ac mauris blandit dapibus. Cras vitae arcu nec lectus volutpat consectetur. Integer condimentum eget libero nec ultrices.

# Subsection

Lorem ipsum dolor sit amet, consectetur adipiscing elit. Nullam vel dolor tincidunt, fermentum velit a, luctus est. Aliquam erat volutpat. Vestibulum ante ipsum primis in faucibus orci luctus et ultrices posuere cubilia Curae; In hac habitasse platea dictumst. Morbi dolor nibh, ultricies sit amet nisl quis, suscipit porttitor lacus. Curabitur eu mauris ac mauris blandit dapibus. Cras vitae arcu nec lectus volutpat consectetur. Integer condimentum eget libero nec ultrices.

# Introduction

Lorem ipsum dolor sit amet, consectetur adipiscing elit. Nullam vel dolor tincidunt, fermentum velit a, luctus est. Aliquam erat volutpat. Vestibulum ante ipsum primis in faucibus orci luctus et ultrices posuere cubilia Curae; In hac habitasse platea dictumst. Morbi dolor nibh, ultricies sit amet nisl quis, suscipit porttitor lacus. Curabitur eu mauris ac mauris blandit dapibus. Cras vitae arcu nec lectus volutpat consectetur. Integer condimentum eget libero nec ultrices.

# Value of the dataset

Lorem ipsum dolor sit amet, consectetur adipiscing elit. Nullam vel dolor tincidunt, fermentum velit a, luctus est. Aliquam erat volutpat. Vestibulum ante ipsum primis in faucibus orci luctus et ultrices posuere cubilia Curae; In hac habitasse platea dictumst. Morbi dolor nibh, ultricies sit amet nisl quis, suscipit porttitor lacus. Curabitur eu mauris ac mauris blandit dapibus. Cras vitae arcu nec lectus volutpat consectetur. Integer condimentum eget libero nec ultrices.

# Subsection

Lorem ipsum dolor sit amet, consectetur adipiscing elit. Nullam vel dolor tincidunt, fermentum velit a, luctus est. Aliquam erat volutpat. Vestibulum ante ipsum primis in faucibus orci luctus et ultrices posuere cubilia Curae; In hac habitasse platea dictumst. Morbi dolor nibh, ultricies sit amet nisl quis, suscipit porttitor lacus. Curabitur eu mauris ac mauris blandit dapibus. Cras vitae arcu nec lectus volutpat consectetur. Integer condimentum eget libero nec ultrices.

# Methods

Lorem ipsum dolor sit amet, consectetur adipiscing elit. Nullam vel dolor tincidunt, fermentum velit a, luctus est. Aliquam erat volutpat. Vestibulum ante ipsum primis in faucibus orci luctus et ultrices posuere cubilia Curae; In hac habitasse platea dictumst. Morbi dolor nibh, ultricies sit amet nisl quis, suscipit porttitor lacus. Curabitur eu mauris ac mauris blandit dapibus. Cras vitae arcu nec lectus volutpat consectetur. Integer condimentum eget libero nec ultrices.

# Sampling

Lorem ipsum dolor sit amet, consectetur adipiscing elit. Nullam vel dolor tincidunt, fermentum velit a, luctus est. Aliquam erat volutpat. Vestibulum ante ipsum primis in faucibus orci luctus et ultrices posuere cubilia Curae; In hac habitasse platea dictumst. Morbi dolor nibh, ultricies sit amet nisl quis, suscipit porttitor lacus. Curabitur eu mauris ac mauris blandit dapibus. Cras vitae arcu nec lectus volutpat consectetur. Integer condimentum eget libero nec ultrices.

# Sample processing

Lorem ipsum dolor sit amet, consectetur adipiscing elit. Nullam vel dolor tincidunt, fermentum velit a, luctus est. Aliquam erat volutpat. Vestibulum ante ipsum primis in faucibus orci luctus et ultrices posuere cubilia Curae; In hac habitasse platea dictumst. Morbi dolor nibh, ultricies sit amet nisl quis, suscipit porttitor lacus. Curabitur eu mauris ac mauris blandit dapibus. Cras vitae arcu nec lectus volutpat consectetur. Integer condimentum eget libero nec ultrices.

# Data processing

Lorem ipsum dolor sit amet, consectetur adipiscing elit. Nullam vel dolor tincidunt, fermentum velit a, luctus est. Aliquam erat volutpat. Vestibulum ante ipsum primis in faucibus orci luctus et ultrices posuere cubilia Curae; In hac habitasse platea dictumst. Morbi dolor nibh, ultricies sit amet nisl quis, suscipit porttitor lacus. Curabitur eu mauris ac mauris blandit dapibus. Cras vitae arcu nec lectus volutpat consectetur. Integer condimentum eget libero nec ultrices.

# Subsection

Lorem ipsum dolor sit amet, consectetur adipiscing elit. Nullam vel dolor tincidunt, fermentum velit a, luctus est. Aliquam erat volutpat. Vestibulum ante ipsum primis in faucibus orci luctus et ultrices posuere cubilia Curae; In hac habitasse platea dictumst. Morbi dolor nibh, ultricies sit amet nisl quis, suscipit porttitor lacus. Curabitur eu mauris ac mauris blandit dapibus. Cras vitae arcu nec lectus volutpat consectetur. Integer condimentum eget libero nec ultrices.

# Biodiversity scope

Lorem ipsum dolor sit amet, consectetur adipiscing elit. Nullam vel dolor tincidunt, fermentum velit a, luctus est. Aliquam erat volutpat. Vestibulum ante ipsum primis in faucibus orci luctus et ultrices posuere cubilia Curae; In hac habitasse platea dictumst. Morbi dolor nibh, ultricies sit amet nisl quis, suscipit porttitor lacus. Curabitur eu mauris ac mauris blandit dapibus. Cras vitae arcu nec lectus volutpat consectetur. Integer condimentum eget libero nec ultrices.

# Target

Lorem ipsum dolor sit amet, consectetur adipiscing elit. Nullam vel dolor tincidunt, fermentum velit a, luctus est. Aliquam erat volutpat. Vestibulum ante ipsum primis in faucibus orci luctus et ultrices posuere cubilia Curae; In hac habitasse platea dictumst. Morbi dolor nibh, ultricies sit amet nisl quis, suscipit porttitor lacus. Curabitur eu mauris ac mauris blandit dapibus. Cras vitae arcu nec lectus volutpat consectetur. Integer condimentum eget libero nec ultrices.

# Taxonomic range

Lorem ipsum dolor sit amet, consectetur adipiscing elit. Nullam vel dolor tincidunt, fermentum velit a, luctus est. Aliquam erat volutpat. Vestibulum ante ipsum primis in faucibus orci luctus et ultrices posuere cubilia Curae; In hac habitasse platea dictumst. Morbi dolor nibh, ultricies sit amet nisl quis, suscipit porttitor lacus. Curabitur eu mauris ac mauris blandit dapibus. Cras vitae arcu nec lectus volutpat consectetur. Integer condimentum eget libero nec ultrices.

# Functional range

Lorem ipsum dolor sit amet, consectetur adipiscing elit. Nullam vel dolor tincidunt, fermentum velit a, luctus est. Aliquam erat volutpat. Vestibulum ante ipsum primis in faucibus orci luctus et ultrices posuere cubilia Curae; In hac habitasse platea dictumst. Morbi dolor nibh, ultricies sit amet nisl quis, suscipit porttitor lacus. Curabitur eu mauris ac mauris blandit dapibus. Cras vitae arcu nec lectus volutpat consectetur. Integer condimentum eget libero nec ultrices.

# Traits

Lorem ipsum dolor sit amet, consectetur adipiscing elit. Nullam vel dolor tincidunt, fermentum velit a, luctus est. Aliquam erat volutpat. Vestibulum ante ipsum primis in faucibus orci luctus et ultrices posuere cubilia Curae; In hac habitasse platea dictumst. Morbi dolor nibh, ultricies sit amet nisl quis, suscipit porttitor lacus. Curabitur eu mauris ac mauris blandit dapibus. Cras vitae arcu nec lectus volutpat consectetur. Integer condimentum eget libero nec ultrices.

# Subsection

Lorem ipsum dolor sit amet, consectetur adipiscing elit. Nullam vel dolor tincidunt, fermentum velit a, luctus est. Aliquam erat volutpat. Vestibulum ante ipsum primis in faucibus orci luctus et ultrices posuere cubilia Curae; In hac habitasse platea dictumst. Morbi dolor nibh, ultricies sit amet nisl quis, suscipit porttitor lacus. Curabitur eu mauris ac mauris blandit dapibus. Cras vitae arcu nec lectus volutpat consectetur. Integer condimentum eget libero nec ultrices.

# Data Statistics

Lorem ipsum dolor sit amet, consectetur adipiscing elit. Nullam vel dolor tincidunt, fermentum velit a, luctus est. Aliquam erat volutpat. Vestibulum ante ipsum primis in faucibus orci luctus et ultrices posuere cubilia Curae; In hac habitasse platea dictumst. Morbi dolor nibh, ultricies sit amet nisl quis, suscipit porttitor lacus. Curabitur eu mauris ac mauris blandit dapibus. Cras vitae arcu nec lectus volutpat consectetur. Integer condimentum eget libero nec ultrices.

# Subsection

Lorem ipsum dolor sit amet, consectetur adipiscing elit. Nullam vel dolor tincidunt, fermentum velit a, luctus est. Aliquam erat volutpat. Vestibulum ante ipsum primis in faucibus orci luctus et ultrices posuere cubilia Curae; In hac habitasse platea dictumst. Morbi dolor nibh, ultricies sit amet nisl quis, suscipit porttitor lacus. Curabitur eu mauris ac mauris blandit dapibus. Cras vitae arcu nec lectus volutpat consectetur. Integer condimentum eget libero nec ultrices.

# Caveats and Limitations

Lorem ipsum dolor sit amet, consectetur adipiscing elit. Nullam vel dolor tincidunt, fermentum velit a, luctus est. Aliquam erat volutpat. Vestibulum ante ipsum primis in faucibus orci luctus et ultrices posuere cubilia Curae; In hac habitasse platea dictumst. Morbi dolor nibh, ultricies sit amet nisl quis, suscipit porttitor lacus. Curabitur eu mauris ac mauris blandit dapibus. Cras vitae arcu nec lectus volutpat consectetur. Integer condimentum eget libero nec ultrices.

# Subsection

Lorem ipsum dolor sit amet, consectetur adipiscing elit. Nullam vel dolor tincidunt, fermentum velit a, luctus est. Aliquam erat volutpat. Vestibulum ante ipsum primis in faucibus orci luctus et ultrices posuere cubilia Curae; In hac habitasse platea dictumst. Morbi dolor nibh, ultricies sit amet nisl quis, suscipit porttitor lacus. Curabitur eu mauris ac mauris blandit dapibus. Cras vitae arcu nec lectus volutpat consectetur. Integer condimentum eget libero nec ultrices.

# Usage Rights

Lorem ipsum dolor sit amet, consectetur adipiscing elit. Nullam vel dolor tincidunt, fermentum velit a, luctus est. Aliquam erat volutpat. Vestibulum ante ipsum primis in faucibus orci luctus et ultrices posuere cubilia Curae; In hac habitasse platea dictumst. Morbi dolor nibh, ultricies sit amet nisl quis, suscipit porttitor lacus. Curabitur eu mauris ac mauris blandit dapibus. Cras vitae arcu nec lectus volutpat consectetur. Integer condimentum eget libero nec ultrices.

# Subsection

Lorem ipsum dolor sit amet, consectetur adipiscing elit. Nullam vel dolor tincidunt, fermentum velit a, luctus est. Aliquam erat volutpat. Vestibulum ante ipsum primis in faucibus orci luctus et ultrices posuere cubilia Curae; In hac habitasse platea dictumst. Morbi dolor nibh, ultricies sit amet nisl quis, suscipit porttitor lacus. Curabitur eu mauris ac mauris blandit dapibus. Cras vitae arcu nec lectus volutpat consectetur. Integer condimentum eget libero nec ultrices.

# Data Resources

Lorem ipsum dolor sit amet, consectetur adipiscing elit. Nullam vel dolor tincidunt, fermentum velit a, luctus est. Aliquam erat volutpat. Vestibulum ante ipsum primis in faucibus orci luctus et ultrices posuere cubilia Curae; In hac habitasse platea dictumst. Morbi dolor nibh, ultricies sit amet nisl quis, suscipit porttitor lacus. Curabitur eu mauris ac mauris blandit dapibus. Cras vitae arcu nec lectus volutpat consectetur. Integer condimentum eget libero nec ultrices.

# Resource

Lorem ipsum dolor sit amet, consectetur adipiscing elit. Nullam vel dolor tincidunt, fermentum velit a, luctus est. Aliquam erat volutpat. Vestibulum ante ipsum primis in faucibus orci luctus et ultrices posuere cubilia Curae; In hac habitasse platea dictumst. Morbi dolor nibh, ultricies sit amet nisl quis, suscipit porttitor lacus. Curabitur eu mauris ac mauris blandit dapibus. Cras vitae arcu nec lectus volutpat consectetur. Integer condimentum eget libero nec ultrices.

# Add section

Lorem ipsum dolor sit amet, consectetur adipiscing elit. Nullam vel dolor tincidunt, fermentum velit a, luctus est. Aliquam erat volutpat. Vestibulum ante ipsum primis in faucibus orci luctus et ultrices posuere cubilia Curae; In hac habitasse platea dictumst. Morbi dolor nibh, ultricies sit amet nisl quis, suscipit porttitor lacus. Curabitur eu mauris ac mauris blandit dapibus. Cras vitae arcu nec lectus volutpat consectetur. Integer condimentum eget libero nec ultrices.

# Subsection

Lorem ipsum dolor sit amet, consectetur adipiscing elit. Nullam vel dolor tincidunt, fermentum velit a, luctus est. Aliquam erat volutpat. Vestibulum ante ipsum primis in faucibus orci luctus et ultrices posuere cubilia Curae; In hac habitasse platea dictumst. Morbi dolor nibh, ultricies sit amet nisl quis, suscipit porttitor lacus. Curabitur eu mauris ac mauris blandit dapibus. Cras vitae arcu nec lectus volutpat consectetur. Integer condimentum eget libero nec ultrices.

# Acknowledgements

Lorem ipsum dolor sit amet, consectetur adipiscing elit. Nullam vel dolor tincidunt, fermentum velit a, luctus est. Aliquam erat volutpat. Vestibulum ante ipsum primis in faucibus orci luctus et ultrices posuere cubilia Curae; In hac habitasse platea dictumst. Morbi dolor nibh, ultricies sit amet nisl quis, suscipit porttitor lacus. Curabitur eu mauris ac mauris blandit dapibus. Cras vitae arcu nec lectus volutpat consectetur. Integer condimentum eget libero nec ultrices.

# Endnotes

Lorem ipsum dolor sit amet, consectetur adipiscing elit. Nullam vel dolor tincidunt, fermentum velit a, luctus est. Aliquam erat volutpat. Vestibulum ante ipsum primis in faucibus orci luctus et ultrices posuere cubilia Curae; In hac habitasse platea dictumst. Morbi dolor nibh, ultricies sit amet nisl quis, suscipit porttitor lacus. Curabitur eu mauris ac mauris blandit dapibus. Cras vitae arcu nec lectus volutpat consectetur. Integer condimentum eget libero nec ultrices.

# References

- Smith V, Georgiev T, Stoev P, Biserkov J, Miller J, Livermore L, Baker E, Mietchen D, Couvreur T, Mueller G, Dikow T, Helgen K, Frank J, Agosti D, Roberts D, Penev L (2013) Beyond dead trees: integrating the scientific process in the Biodiversity Data Journal. Biodiversity Data Journal 1: e995. doi: 10.3897/BDJ.1.e995
- Roettger S, Schulz M, Bartelheimer W, Ertl T (2001) Automotive Soiling Simulation Based On Massive Particle Tracing. The Eurographics Association 3: 125. DOI: 10.2312/VISSYM/VISSYM01/309-318

Compose Message

Subject \*

Recipients \*

Message \*

Editor toolbarsBasic Styles BoldKeyboard shortcut Ctrl+B ItalicKeyboard shortcut Ctrl+I UnderlineKeyboard shortcut Ctrl+U Subscript Superscript Insert/Remove Numbered List Insert/Remove Bulleted List Insert Special Character LinkKeyboard shortcut Ctrl+L Unlink Decrease Indent Increase Indent Spell Check As You Type UndoKeyboard shortcut Ctrl+Z RedoKeyboard shortcut Ctrl+Y

Send

Close
