# Supplementary material for: A streamlined workflow for conversion, peer review, and publication of genomics metadata as omics data papers
Source: Gigascience. 2021 May 13;10(5):giab034. doi: 10.1093/gigascience/giab034 (PMC8117446; doi:10.1093/gigascience/giab034)
Supplement: giab034_Response_to_Reviewer_Comments_Original_Submission [file giab034_response_to_reviewer_comments_original_submission.pdf]

Author's Response To Reviewer Comments

Close

Dear Editor and Reviewers,

Thank you for taking the time and effort to review our manuscript again! We highly appreciate your constructive feedback in this and the previous review rounds. Your thoughtful comments helped us make some important changes.

You advised us to submit the R shiny app to https://bio.tools and https://SciCrunch.org databases to receive RRID (Research Resource Identification Initiative ID) and biotoolsID identifiers and we have done so. The identifiers are: biotools:omics-data-paper-shinyapp-golem for biotools and SCR_019809 for SciCrunch.org. We have updated the manuscript with these identifiers in the "Availability of supporting source code and requirements" section on page 28.

Below you will find an explanation of all other modifications we made to the manuscript, as well as detailed responses to your comments. You can read our response also as a document, which is attached to our resubmission.

Question 1: Do data paper undergo peer review?
if so, what is the acceptance rate?
Scientists claims that they are over burden with data management tasks, from data deposition to manuscript writing.
Could the authors indicate how much work a data paper represents in contrast to a classic scientific article?

Response:

Similarly to all other manuscript types in Pensoft journals, all data papers undergo a rigorous peer review (see https://bdj.pensoft.net/about#DataReviewGuidelines). In addition, prior to peer review they are also subjected to data auditing by a data auditor at Pensoft who makes sure that data is accessible and its quality is of high standard according to a data quality checklist (https://bdj.pensoft.net/about#DataQualityChecklistandRecommendations). The submitted manuscript contains information about the peer review and data audit of the genomics data papers. We have indicated Pensoft's data auditing and peer-review processes in the "Omics data papers and underlying datasets undergo peer-review and data auditing" subsection of the Discussion section on page 23.

The acceptance rate of the Biodiversity Data Journal varies between 70-80%.

In terms of the effort that data papers require from authors, it really depends on the nature of the data and the author's preference for including a certain number of data statistics and meta-analyses to describe their data. Data papers do not require authors to include an analysis of the dataset but they can do so if they wish, as long as the data paper does not become too long-drawn-out. The key aspect of a data paper is the detailed description of the data following the community accepted standards and FAIRness and quality of the data itself, therefore authors' main efforts should go towards making sure that the described datasets are consistent throughout, well-described in terms of methodology and openly available and accessible. The goal of the data paper is to make the dataset fully reusable so that other researchers can repeat the experiment or collate the published data with other datasets to generate new hypotheses and research results.

The benefits of publishing data papers are listed extensively in the section "Data papers for the field of omics: rationale and benefits" (page 22) of the manuscript. In the first version of the manuscript, we had included a figure (Please see the attached cover letter for Fig. 1) which shows a clear increasing trend in popularity and use of data papers as a useful method of data publishing and a powerful instrument for improving research integrity and data reuse. For example the Biodiversity Data Journal

alone, shows the following numbers in publication of data papers: 2018 (9), 2019 (31). For 2020 (not pictured in the figure), there were 68 data papers, which indicates an yearly increase of more than 100 % in the last year alone.

Question 2: Following creation of the populated backbone, how much write-up are authors expected to do to reach minimal content and quality requirements?
I understand the authors somehow tackle this issue on pages 22-26 but I am still concerned about the economics and sustainability of the approach.
The authors indicate that OMIC data paper are audited, which means curation time is expanded. This is an expensive task to accomplish and requires domain experts.
It would be interesting to cover some of these aspects in the discussion, all the more so finding reviewers is becoming harder and harder (which would be also require finding data experts).

Response:

Data auditing is indeed an expensive task but Pensoft strives towards publishing high quality datasets and has fully committed to this practice, provided by in-house data auditors. The data auditors are biologists with extensive experience with data handling, storing and processing. They ensure that the datasets follow some basic guidelines to ensure their FAIRness and consistency. They provide authors with tips on how to make their data FAIR, if it is not FAIR yet. Data auditors do not reproduce data generation and analysis if there is such. This would be the role of peer-reviewers. Finally, data auditors are permanently employed and unlike peer-reviewers do not have to be sought out on a case by case basis, which significantly reduces the waiting time. Therefore, we cannot really compare the process of finding reviewers with the process of finding data auditors. We have included a paragraph in the manuscript about how the system works ("Omics data papers and underlying datasets undergo peer-review and data auditing" subsection on page 23), with the relevant citations of the respective data quality guidelines of the Biodiversity Data Journal,. Perhaps, the intricacies of data paper publishing, including finding data auditors, can be explored in more detail in a separate publication but here we wanted to focus on genomic data papers and the workflow. More information about the data auditing process at Pensoft can be found in this blog (also cited in the discussion part of the paper): https://www.eurekalert.org/pub_releases/2019-10/pp-aif101819.php

Question 3: Is there a specific mechanism used in Pensoft OMIC Data Paper to reference database accession numbers so those can be easily identified and extracted?

Response:

Database accession numbers have to be referenced in the Data Resources section of the omics data paper (Please see the attached cover letter for Fig. 2). The Resource identifier field, where such an accession number should be entered, is a required field of the template, meaning that it cannot be submitted without being filled in. If using the omics data paper conversion workflow, this field is automatically populated with an ENA sequence identifier.

After publication, all articles in BDJ are publicly available also as JATS XML and submitted as such for archiving and display to PubMedCentral, in addition to the traditional PDF and HTML formats. Therefore, all identifiers and links to external databases are marked up in the XML version of the article and can then be indexed by Web crawlers, including Pensoft's own RDFization scripts which transform XML articles to Resource Description Framework (RDF) to ensure machine readability of the text (Penev et al., 2019).

Question 4: Have the authors also considered an automated action to update a database (e.g. ENA) record with a DOI associated with a Pensoft `OMIC data paper`? This comes back to the issue of producing an entirely new artifact, possibly one containing 90 % of the information stored in a SRA XML document but presented in a JATS documents.
Were EMBL-EBI records to be given DOIs, would it impact the OMIC Data Paper model ?

Response: We have considered this possibility as an excellent way to link data and literature both ways. There were some preliminary discussions with ENA to at least provide a back linking from the published data paper to the relevant resource at ENA and we expect to achieve that during the starting EU-funded BiCIKL project, coordinated by Pensoft. During that project, EMBL-EBI should provide an interface to

edit/improve the metadata of already submitted datasets from annotations to these, data papers included, posted post factum.

Pensoft has a proven experience in this kind of workflow with GBIF, For example, the discrepancies in data already indexed in GBIF found by our data auditors are sent to the authors with a clear demand to correct the data in the GBIf indexed dataset before the manuscript will be forwarded to peer review in the journal.

If EMBL-EBI records are given (DataCite) DOIs, which of no doubt would be an excellent practice, this would not create any issues for the workflow because they would be just different identifier records than the data papers. This is because the original dataset is a separate entity than the data paper in which it is described.

Question 5: Dealing with large datasets.

Some studies may contain hundreds, thousands of samples. The interaction with the AWT indicates that the OMIC data article would contain a long list of biomaterials, and associated resources.

Have the authors considered aspect of stacking / compressing information or have they considered automated sentence generation to assist authors in expanding the text for an OMIC data paper ?

Response:

We have thought about large datasets and this is why we attach the BioSamples checklist as a supplementary file and not as a table within the narrative itself. When it comes to sentence generation, this is not really needed because we do not require separate description of each and every sample. Authors can aggregate their description of large datasets to create a concise and clear manuscript.

Actually, the sample size does not directly affect the size of the data paper, because the data are stored outside the journal's infrastructure, which hosts the narrative of the data paper. Large data sets would require a bit more detailed text description and explanations, however this is a normal practice in the manuscript authoring and publishing process.

Question 6. License on the data

Shouldn't there be a filled in section in the OMIC data paper to specific the nature of the license under which data and resources are made available?
this is possible to auto-generated from public repositories and, if authors create a manuscript from scratch, they should be offered options.
This is distinct from the license attached to the manuscript itself or to the metadata.

Response: There is already such a section, called "Usage rights", where authors can specify the license of the data. It is implemented both in Pensoft's ARPHA template and in the R shiny app (Please See the attached cover letter for Fig. 3).

We have not implemented a dropdown menu or other kind of entry form with limited options for licenses because there might be a case in which different datasets, described in the data paper, have different (open-source) licenses so it would be best if the authors specify that manually. As stated in the author guidelines of BDJ, only datasets with open-source licenses can be published as part of data paper publications in Pensoft's journals (Please see the attached cover letter for Fig. 4).

----
minor corrections:

page 5, "MIxS consists of three checklists, each
containing several packages for the description of various environments where
genomic material could be sampled from [22]."

- >overall, check for similar missing elements of punctuation.

Response:
We have corrected this mistake and have checked for other punctuation mistakes throughout the manuscript.

page 6: "A more comprehensive approach towards omics metadata mobilisation is undertaken by the ISA Commons community [28], who ",

-> s/who/which/

Response:

We have corrected this in the manuscript.

page 6: "(1) data publishing through international trusted data repositories, such as INSDC [7], GBIF [9], and others, a.. "

-> drop "and others" to simplify the sentence.

Response:

We have corrected this in the manuscript.

page 7: "Furthermore, it ensures a scientific record, crediting and acknowledgement for the data creators and scientists in the form of citable scholarly articles."

rephrase or complete, eg: "Furthermore, it ensures that a citable scholarly scientific record, crediting and acknowledging the daat creators.... is created'.

Response:

We have rephrased this sentence to make it more clear while preserving the meaning. The new sentence is: “Furthermore, it creates a citable scientific record, enabling the crediting and acknowledgement of the data creators and researchers.”

page 7: "As more and more researchers want to deposit and share their datasets, standards, infrastructures, and workflows become central to delivering FAIR data.

rephrase, eg: "As more and more researchers want to deposit and share their datasets, new tools and new approaches are needed to deliver FAIR data.

Response:

We have rephrased this sentence according to your suggestion.

page 8: "to describe a prototyped workflow"
-> s/prototyped/prototype/

Response:

We have corrected this sentence in the manuscript.

page 9: "We created a template, "
-> "We created a dedicated data article template, "

Response:

We have corrected this sentence in the manuscript to “We created a dedicated data paper template”...

page 23, section 1: "is an meticulous approach to", s/an/a/

Response:

We have corrected this mistake in the manuscript.

page 24, section 2: "Throughout our testing phase", add a comma after 'phase'

Response:

We have corrected this mistake in the manuscript.

Close
