# Supplementary material for: A streamlined workflow for conversion, peer review, and publication of genomics metadata as omics data papers
Source: Gigascience. 2021 May 13;10(5):giab034. doi: 10.1093/gigascience/giab034 (PMC8117446; doi:10.1093/gigascience/giab034)
Supplement: giab034_Reviewer_1_Report_Original_Submission — Philippe Rocca-Serra -- 1/4/2021 Reviewed [file giab034_reviewer_1_report_original_submission.pdf]

Reviewer Report

Title: A streamlined workflow for conversion, peer review and publication of genomics metadata as Omics Data Papers

Version: Original Submission **Date:** 1/4/2021

Reviewer name: Philippe Rocca-Serra

Reviewer Comments to Author:

I would first would like to apologise to the authors, the editors and reviewers for my very late review, for which I will blame the end of year "maelstrom".
I would like also to thank the authors for their efforts in accommodating the numerous requests from the 3 reviewers.
As a result, we feel the manuscript is very much improved In particular, the clarification of the underlying need of affording means to track dataset citation by piggy backing on the well established schorlarly publications tracking.
However, there are still in my views a number of outstanding questions:
1. Question: Do data paper undergo peer review?
if so, what is the acceptance rate?
Scientists claims that they are over burden with data management tasks, from data deposition to manuscript writing.
Could the authors indicate how much work a data paper represents in contrast to a classic scientific article?
2. Following creation of the populated backbone, how much write-up are authors expected to do to reach minimal content and quality requirements?
I understand the authors somehow tackle this issue on pages 22-26 but I am still concerned about the economics and sustainability of the approach.
The authors indicate that OMIC data paper are audited, which means curation time is expanded. This is an expensive task to accomplish and requires domain experts.
It would be interesting to cover some of these aspects in the discussion, all the more so finding reviewers is becoming harder and harder (which would be also require finding data experts).
3. is there a specific mechanism used in Pensoft OMIC Data Paper to reference database accession numbers so those can be easily identified and extracted?
4. Have the authors also considered an automated action to update a database (e.g. ENA) record with a DOI associated with a Pensoft `OMIC data paper`?
This comes back to the issue of producing an entirely new artifact, possibly one containing 90 % of the information stored in a SRA XML document but presented in a JATS documents.
Were EMBL-EBI records to be given DOIs, would it impact the OMIC Data Paper model ?
5. Dealing with large datasets.
Some studies may contain hundreds, thousands of samples. The interaction with the AWT indicates that

the OMIC data article would contain a long list of biomaterials, and associated resources.
Have the authors considered aspect of stacking / compressing information or have they considered automated sentence generation to assist authors in expanding the text for an OMIC data paper ?
6. License on the data:
Shouldn't there be a filled in section in the OMIC data paper to specific the nature of the license under which data and resources are made available?
this is possible to auto-generated from public repositories and, if authors create a manuscript from scratch, they should be offered options.
This is distinct from the license attached to the manuscript itself or to the metadata.

minor corrections:

----

page 5, "MIxS consists of three checklists, each
containing several packages for the description of various environments where
genomic material could be sampled from [22]."
- >overall, check for similar missing elements of punctuation.
page 6: "A more comprehensive approach towards omics metadata mobilisation is undertaken
by the ISA Commons community [28], who ",
-> s/who/which/
page 6: "(1) data publishing through international trusted data
repositories, such as INSDC [7], GBIF [9], and others, a.. "
-> drop "and others" to simplify the sentence.
page 7: "Furthermore, it ensures a scientific record, crediting and acknowledgement for the data creators and scientists in the form of citable scholarly articles."
rephrase or complete, eg: "Furthermore, it ensures that a citable scholarly scientific record, crediting and acknowledging the daat creators.... is created'.
page 7: "As more and more researchers want to deposit and share their datasets, standards, infrastructures, and workflows become central to delivering FAIR data.
rephrase, eg: "As more and more researchers want to deposit and share their datasets, new tools and new approaches are needed to deliver FAIR data.
page 8: "to describe a prototyped workflow"
-> s/prototyped/prototype/
page 9: "We created a template, "
-> "We created a dedicated data article template, "
page 23, section 1: "is an meticulous approach to", s/an/a/
page 24, section 2: "Throughout our testing phase", add a comma after 'phase'

Kind regards
Philippe

Level of Interest

Please indicate how interesting you found the manuscript: Choose an item.

Quality of Written English

Please indicate the quality of language in the manuscript: Choose an item.

Declaration of Competing Interests

Please complete a declaration of competing interests, considering the following questions:

- Have you in the past five years received reimbursements, fees, funding, or salary from an organisation that may in any way gain or lose financially from the publication of this manuscript, either now or in the future?
- Do you hold any stocks or shares in an organisation that may in any way gain or lose financially from the publication of this manuscript, either now or in the future?
- Do you hold or are you currently applying for any patents relating to the content of the manuscript?
- Have you received reimbursements, fees, funding, or salary from an organization that holds or has applied for patents relating to the content of the manuscript?
- Do you have any other financial competing interests?
- Do you have any non-financial competing interests in relation to this paper?

If you can answer no to all of the above, write 'I declare that I have no competing interests' below. If your reply is yes to any, please give details below.

I have possibly competing interests, which i list below: 1. Associate Editor of Nature Springer Scientific Data 2. Developer of ISA tools and Datascriptor , which are referenced in the manuscript

I agree to the open peer review policy of the journal. I understand that my name will be included on my report to the authors and, if the manuscript is accepted for publication, my named report including any attachments I upload will be posted on the website along with the authors' responses. I agree for my report to be made available under an Open Access Creative Commons CC-BY license (http://creativecommons.org/licenses/by/4.0/). I understand that any comments which I do not wish to be included in my named report can be included as confidential comments to the editors, which will not be published.

Choose an item.

To further support our reviewers, we have joined with Publons, where you can gain additional credit to further highlight your hard work (see: https://publons.com/journal/530/gigascience). On publication of this paper, your review will be automatically added to Publons, you can then choose whether or not to claim your Publons credit. I understand this statement.

Yes Choose an item.
